# Anticancer Meroterpenoids from *Centrapalus pauciflorus* leaves: Chromone- and 2,4-Chromadione-Monoterpene Derivatives

**DOI:** 10.1021/acsomega.3c03884

**Published:** 2023-08-16

**Authors:** Gordana Krstić, Muhammad Bello Saidu, Anita Barta, Máté Vágvölgyi, Hazhmat Ali, István Zupkó, Róbert Berkecz, Umar Shehu Gallah, Dóra Rédei, Judit Hohmann

**Affiliations:** † Department of Pharmacognosy, University of Szeged, Eötvös u. 6, 6720 Szeged, Hungary; ‡ University of Belgrade, Faculty of Chemistry, Studentski trg 12-16, 11158 Belgrade, Serbia; § Institute of Pharmacodynamics and Biopharmacy, University of Szeged, Eötvös u. 6, 6720 Szeged, Hungary; ∥ Institute of Pharmaceutical Analysis, University of Szeged, Somogyi u. 4, 6720 Szeged, Hungary; ⊥ Bioresource Department, National Research Institute for Chemical Technology (NARICT), Zaria 1052, Nigeria; ∇ ELKH-USZ Biologically Active Natural Products Research Group, University of Szeged, Eötvös u. 6, 6720 Szeged, Hungary

## Abstract

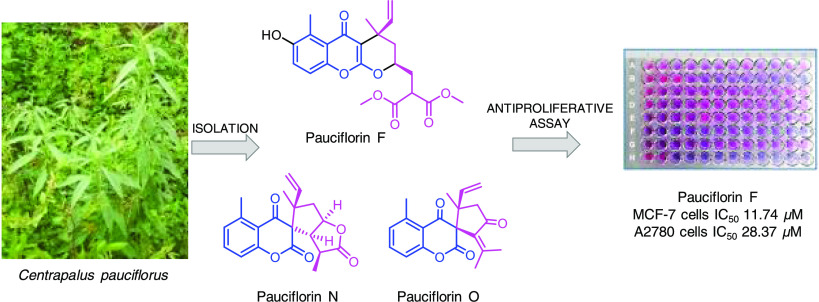

Eight previously undescribed chromones, named pauciflorins F–M and two 5-methyl-2,4-chromadione derivatives named as pauciflorins N and O, were isolated from the methanol extract of the leaves of *Centrapalus pauciflorus* (Willd.) H.Rob. together with the known (+)-spiro-ethuliacoumarin. The structures were determined via extensive spectroscopic analyses, including HRESIMS, 1D NMR (^1^H, ^13^C JMOD), and 2D NMR (HSQC, HMBC, ^1^H–^1^H COSY, and NOESY) experiments. Through an MTT assay, seven isolated compounds were tested for their antiproliferative properties against human adherent breast (MCF-7, MDA-MB-231), cervical (HeLa, SiHa), and ovarian (A2780) cancer cell lines. Pauciflorin F was effective against MCF-7 breast cancer cells, its activity (IC_50_ 5.78 μM) was comparable to that of the reference agent cisplatin (IC_50_ 5.78 μM).

## Introduction

Meroterpenoids are a group of terpenoid-containing hybrid natural products with unique structural architectures and impressive pharmacological properties. Meroterpenoids can be classified based on their nonterpenoid moiety into polyketide-terpenoids, shikimate-terpenoids, and alkaloid-terpenoids. These compounds are synthesized by a wide range of organisms, including bacteria, fungi, algae, plants, animals, and marine organisms.^1−4^ Notably, meroterpenoids derived from chromane/chromene can be condensed with hemi-, mono-, sesqui-, and diterpenoid units; their presence has been observed in various organisms, such as tunicates (*Botryllus*),^5^ brown macroalgae *(Sargassum, Cystoseira)*,^6^ and *Rhododendron* (Ericaceae),^7^*Sarcandra* (Chloranthaceae),^8^ and *Mimosa* (Fabaceae)^9^ plant species. In addition, the genera of the Asteraceae family have been discovered to produce uncommon monoterpenoid-coupled chromones, which typically coexist with structurally related monoterpenoid coumarins.^10−12^ These intriguing compounds have been identified in genera such as *Nassauvia*,^13^*Triptilion*,^14^ and *Polyachyrus* from the Nassauvieae tribe, *Gerbera*(11) and *Mutisia*(15) from the Mutisieae tribe, and *Bothriocline* genus^16^ from the Vernonieae tribe and reported to accumulate as both coumarin- and chromon-based meroterpenoids. The biosynthesis of these compounds involves the acetate–malonate pathway, where 5-methylcoumarins and 5-methylchromones serve as the main building blocks for chromone-, coumarin-based meroterpenoids, which is catalyzed by polyketide synthase enzymes.^17^ Chromane/chromene meroterpenoids have displayed cytotoxic activity against different tumor cell lines, as well as antioxidant and antimicrobial activities. Furthermore, they have been reported to inhibit various enzymes, including protein tyrosine phosphatase 1B (PTP1B), butyrylcholinesterase (BChE), β-site amyloid precursor protein cleavage enzyme 1 (BACE1), and protein farnesyl transferase (PFTase).^1,18^

Continuing our ongoing investigation on the metabolites of *Centrapalus pauciflorus* (Willd.) H.Rob.,^19,20^ this study reports the isolation and structure determination of eight chromone-meroterpenoids named pauciflorins F–M (**1**–**10**) and two 5-methyl-2,4-chromadione-meroterpenoids pauciflorins N and O (**10**–**11**), extracted from the leaves of *C*. *pauciflorus*. This plant, belonging to the Asteraceae family, is also known by synonymous names, such as *Centrapalus galamensis* Cass., *Conyza pauciflora* Willd., *Vernonia afromontana* R.E.Fr, and *Vernonia pauciflora* (Willd.) Less., etc. *C*. *pauciflorus* is native to tropical African countries, spanning from Cape verde and Senegal in West Africa to Somalia in East Africa and reaching down to Southern Africa, encompassing Zimbabwe and Mozambique.^21,22^ It is a mainly unbranched, annual plant that grows 3–5 m tall. In folklore medicine, its leaves are cooked in porridge or brewed as tea to alleviate chest pain. The plant is also used to relieve stomach pain.^20^

## Materials and Methods

### General Experimental Procedures

The optical rotations were determined using a JASCO P-2000 polarimeter (JASCO International Co. Ltd., Hachioji, Tokyo, Japan). NMR spectra were recorded on a Bruker Avance DRX 500 spectrometer at 500 MHz (^1^H) and 125 MHz (^13^C). The two-dimensional (2D) experiments were conducted using standard Bruker software. Gradient-enhanced versions were applied in correlation spectroscopy (^1^H–^1^H COSY), nuclear Overhauser effect spectroscopy (NOESY), heteronuclear single quantum coherence spectroscopy (HSQC), and heteronuclear multiple bond correlation (HMBC) experiments. The signals of the deuterated solvent were taken as references. High-resolution electrospray ionization–mass spectroscopy (HRESIMS) was measured using a Thermo Scientific Q-Exactive Plus Orbitrap mass spectrometer in positive ionization mode equipped with an electrospray ionization source. The data were acquired and processed using MassLynx software. Vacuum liquid chromatography (VLC) was made on silica gel (15 μm, Merck) (NP-VLC); LiChroprep RP-18 (40–63 μm, Merck) stationary phase was used for reversed-phase VLC (RP-VLC); open column chromatography (OCC) was conducted on polyamide (MP Biomedicals). Flash column chromatography (FCC) was performed using a CombiFlash Rf+ Lumen instrument with integrated ultraviolet (UV), UV–visible (UV–vis), and electrophoretic light scattering detection using a column (3.5 cm × 14 cm) filled with 40 g of reversed-phase (RP) silica C_18_. Thin-layer chromatography (TLC) monitored the OCC, VLC, and FCC separations and was carried out on silica gel 60 F_254_ plates (Merck). High-performance liquid chromatography (HPLC) was performed on Agilent, WUFENG, and WATERS HPLC instruments using normal-phased (NP) LiChrospher Si 60 (4 mm × 250 mm, 5 μm) and Luna (R) Silica (2) 100 (250 mm × 21.2 mm, 5 μm), as well as RP Kinetex C_18_ 100A (4.6 mm × 150 mm, 5 μm) and Agilent ZORBAX ODS C_18_ 100A (9.4 mm × 250 mm, 5 μm)] columns. The TLC plates were detected under a UV light at 254 nm by spraying with concentrated H_2_SO_4_, followed by heating. All solvents used for chromatography were analytical or HPLC grade (VWR Ltd., Hungary).

### Plant Material

The leaves of the plant were gathered in August 2018 in Zaria, Nigeria (11°7′19.758″N 7°43′23.1672″E) and were identified by Umar Shehu Gallah (National Research Institute for Chemical Technology, NARICT), Zaria, Nigeria. A voucher specimen was deposited in NARICT under the number Narict/Biores/321 and in the Herbarium of the Department of Pharmacognosy, University of Szeged, Szeged, Hungary, No. 897.

### Extraction and Isolation

The air-dried and powdered leaves of the plant (548 g) was extracted using percolation with methanol (45 L) at room temperature until all possible extract was obtained. The methanol extract was concentrated in a vacuum to yield 133 g of extract, representing 24.3% of the plant material. The extract was dissolved in 1 L MeOH–H_2_O (1:1) and subjected to solvent–solvent partition with CHCl_3_ (3 × 1 L) to yield the lipophilic phase. After concentration, the CHCl_3_ phase (65.81 g) was separated using OCC on polyamide (250 g), eluting with methanol–water (1:4, 2:3, 3:2, 4:1, and 5:0) mixtures as eluents. Five fractions were collected according to the eluents. The fraction obtained with MeOH–H_2_O (3:2) showed the highest antiproliferative activity against MCF-7, MDA-MB-231, HeLa, and A2780 cell lines with growth inhibition of 70.7%, 85.3%, 63.7%, and 68.2%, respectively at 30 μg/mL,^19^ and it was chosen for further chromatographic purification. VLC was conducted on that fraction (14 g) on silica gel using a gradient system of cyclohexane–EtOAc–EtOH (9:1:0, 8:2:0, 7:3:0, 50:20:1.5, 50:20:3, 50:20:6, 50:20:9, 50:20:12, 50:20:15, 5:2:2, 5:2:4, 5:2:6, and 5:2:8), which yielded fractions A–I. Fractions A–C obtained with elution of cyclohexane–EtOAc–EtOH (8:2:0, 50:20:1.5 and 50:20:3) were further chromatographed on NP- and RP-VLC as follows.

NP-VLC was conducted on fraction A using a gradient system of cyclohexane–EtOAc (98:2 to 80:20) as eluent. Two subfractions were obtained A/I and A/II. Subfraction A/I was purified further using RP-HPLC, affording nine fractions A/I/1–9. Further purification of fraction A/I/9 on NP-HPLC with *n*-hexane–EtOAc (95:5) as mobile phase furnished compound **7** (0.9 mg, R_t_ 7.70 min).

Fraction B was separated via RP-VLC using MeOH–H_2_O mixtures (from 4:6 to 9:1) as eluents, affording subfractions B/I–III. Subfraction B/II was subjected to NP-VLC with an *n*-hexane–CHCl_3_ gradient system (from 9:1 to 3:7), yielding subfractions B/II/1–2. Subfraction B/II/2 was further purified using NP-VLC with cyclohexane–EtOAc (from 98:2 to 80:20) mixtures as eluents, isolating four fractions denoted as B/II/2/a–d. The following RP-HPLC purification of B/II/2/c using MeOH–H_2_O (68:32) mixtures as mobile phase led to the isolation of compound **2** (15.2 mg, R_t_ 21.50 min). Subfraction B/III was subjected to NP-VLC using a cyclohexane–EtOAc gradient solvent system (from 98:2 to 80:20), affording subfractions B/III/1–2. The NP-HPLC separation of fraction B/III/1 with a mobile phase of *n*-hexane–EtOAc–MeOH (90:9:1) yielded subfractions B/III/1/a–c. RP-HPLC further purified subfraction B/III/1/b with MeOH–H_2_O (75:25) as mobile phase, isolating pure compound **6** (0.9 mg, Rt 17.87 min). Furthermore, the RP-HPLC of B/III/2 using MeOH–H_2_O (78:22) mixtures as mobile phase resulted in the isolation of compounds **5** (10.9 mg, R_t_ 12.80 min) and **4** (32.1 mg, R_t_ 15.20 min).

Fraction C was subjected to RP-FCC with MeOH–H_2_O (from 30:70 to 75:25) mixtures as eluents, isolating seven subfractions, namely C/I–VII. Subfraction C/III was further purified using NP-VLC with a mobile phase of *n*-hexane–CHCl_3_ (from 9:1 to 2:8) resulting subfractions C/III/1–5. Subfraction C/III/1 was subsequently subjected to another round of RP-VLC using MeOH–H_2_O (from 98:2 to 80:20) as eluent, isolating subfractions C/III/1/a–f. Subfraction C/III/1/f yielded compound **11** (2.0 mg) in NP-HPLC analysis using a mobile phase of *n*-hexane–EtOAc–MeOH (80:19:1). Further purification of subfraction C/III/1/f_4_ using RP-HPLC with a mobile phase of MeOH–H_2_O (75:25) isolated compound **10** (0.8 g, R_t_ 17.57 min). Subfraction C/III/5 was subjected to NP-HPLC using a mobile phase of *n*-hexane–EtOAc–MeOH (90:9:1) which generated five fractions denoted as C/III/5/a–e. Compound **8** (4.7 mg, R_t_ 12.60 min) was isolated in pure form from subfraction C/III/5/b via RP-HPLC using MeOH–H_2_O (72:28) as eluent. Furthermore, RP-HPLC analysis of subfraction C/III/5/e applying a solvent system of MeOH–H_2_O (72:28) resulted in the isolation of compounds **1** (12.3 mg, R_t_ 8.20 min) and **3** (0.9 mg, R_t_ 10.40 min).

#### Pauciflorin F (**1**)

Colorless oil; [α]_D_^27^ + 89.1 (*c* 0.1, CHCl_3_); ^1^H and ^13^C NMR data, see Tables 1 and 2; HRESIMS *m*/*z* 417.1538 [M + H]^+^ (calcd for C_22_H_25_O_8_^+^ 417.1544).

**Table 1:** ^1^H NMR Data of Compounds 1–8 [δ ppm (*J* = Hz), CDCl_3_, 500 MHz]

Position	1	2	3	4	5	6	7	8
6	-	7.06 d (7.9)	7.06 d (7.9)	7.06 d (7.5)	7.06 d (7.7)	7.05 d (8.0)	7.08 d (7.8)	-
7	7.07 d (8.9)	7.39 t (7.9)	7.38 t (7.9)	7.38 t (7.5)	7.39 t (7.7)	7.38 t (8.0)	7.40 t (7.8)	7.06 s
8	7.03 d (8.9)	7.14 d (7.9)	7.15 d (7.9)	7.16 d (7.5)	7.16 d (7.7)	7.17 d (8.0)	7.17 d (7.8)	7.06 s
9	2.74 s	2.82 s	2.83 s	2.83 s	2.83 s	2.83 s	2.83 s	2.76 s
1′a	5.10 d (17.5)	5.12 d (17.1)	5.11 d (17.6)	5.12 dd (17.7, 1.9)	5.12 d (17.7)	5.13 d (17.5)	5.14 t (17.4)	5.10 d (17.4)
1′b	5.07 d (10.7)	5.09 d (10.5)	5.08 d (10.8)	5.09 dd (10.6, 1.9)	5.09 d (11.0)	5.09 d (10.6)	5.11 d (10.5)	5.08 d (10.6)
2′	6.13 dd (17.5, 10.7)	6.16 dd (17.1, 10.5)	6.18 dd (17.6, 10.8)	6.19 ddd (17.7, 10.6, 1.9)	6.17 dd (17.7, 11.0)	6.18 dd (17.5, 10.6)	6.18 dd (17.4, 10.5)	6.14 dd (17.4, 10.6)
4′	1.84 dd (14.0, 11.7)	1.86 dd (14.0, 12.0)	1.84 dd (13.8, 11.7)	1.88 dd (14.0, 12.1)	1.85 dd (14.0, 11.8)	1.95 dd (14.2, 12.0)	2.02 dd (14.2, 12.1)	1.82 dd (14.0, 12.0)
	1.70 dd (14.0, 1.5)	1.72 dd (14.0, 1.7)	1.70 dd (13.8, 1.3)	1.70 m	1.70 dd (14.0, 1.5)	1.66 dd (14.2, 1.7)	1.70 dd (14.2, 1.9)	1.68 dd (14.0, 1.6)
5′	4.40 m	4.42 m	4.41 m	4.47 m	4.40 m	5.07 m	5.19 m	4.39 m
6′	2.34 m (2H)	2.35 m (2H)	2.28 m	2.15 m	2.09 m (2H)	5.35 brd (8.5)	6.83 dd (7.9, 1.4)	2.26 m
			1.75 ddd (14.3, 7.0, 4.5)	1.98 m		-	-	1.74 m
7′	3.82 dd (8.4, 6.1)	3.82 dd (8.6, 5.9)	2.79 m	3.00 m	2.62 m (2H)	-	-	2.80 m
8′	-	-	-	-	-	1.82 s	-	-
9′	-	-	1.28 d (7.0)	3.89 m (2H)	-	1.79 s	1.97 s	1.28 d (7.0)
10′	1.55 s	1.57 s	1.57 s	1.57 s	1.57 s	1.62 s	1.64 s	1.56 s
8′–OCH_3_	3.79 s	3.81 s	3.74 s	3.78 s	3.72 s	-	3.80 s	3.74 s
9′–OCH_3_	3.80 s	3.80 s	-	-	-	-	-	
6–OH	5.47 s	-	-	-	-	-	-	5.07 s

**Table 2:** ^13^C NMR Data of Compounds 1-8 (δ ppm, CDCl_3_, 125 MHz)

Position	1	2	3	4	5	6	7	8
2	162.2	162.3	162.6	162.5	162.6	166.0	162.2	162.5
3	103.3	103.7	103.7	103.5	103.7	103.5	103.7	103.3
4	179.7	179.3	179.3	179.8	179.3	179.4	179.3	179.7
5	124.0	141.2	141.2	141.9	141.2	141.5	141.2	123.9
6	151.0	128.0	127.9	128.0	127.9	127.8	128.0	150.8
7	120.4	131.7	131.7	131.7	131.7	131.6	131.9	120.3
8	115.2	115.1	115.1	115.1	115.1	115.2	115.2	115.3
8a	148.5	154.3	154.3	154.0	154.2	154.3	154.3	148.7
4a	122.1	121.7	121.8	121.5	121.7	121.8	121.7	122.2
9	12.7	22.9	22.8	22.8	22.9	22.9	22.9	12.6
1′	111.8	111.8	111.6	111.7	111.7	111.5	111.8	111.6
2′	145.6	145.5	145.8	145.7	145.8	145.8	145.1	145.8
3′	36.5	36.5	36.6	36.6	36.5	36.6	36.5	36.6
4′	43.4	43.4	43.5	43.8	43.3	43.9	42.5	43.5
5′	74.2	74.3	74.7	75.0	75.7	73.9	73.6	74.6
6′	34.1	34.1	38.7	34.3	30.2	122.2	136.9	38.7
7′	47.9	47.9	36.0	43.6	29.6	140.0	131.3	36.0
8′	169.4	169.3	176.5	174.8	173.4	25.9	167.6	176.7
9′	169.6	169.6	17.3	63.7	-	18.7	13.3	17.4
10′	24.9	24.9	25.0	25.0	24.9	24.8	24.8	24.8
8′–OCH_3_	53.0	53.0	51.9	52.2	52.0	-	52.4	52.0
9′–OCH_3_	53.0	53.0	-	-	-	-	-	-

#### Pauciflorin G (**2**)

Colorless oil; [α]_D_^27^ – 73.5 (*c* 0.1, CHCl_3_); ^1^H and ^13^C NMR data, see Tables 1 and 2; positive-ion HRESIMS *m*/*z* 401.1593 [M + H]^+^ (calcd for C_22_H_25_O_7_^+^ 401.1595), 423.1413 (calcd for C_22_H_24_O_7_Na 423.1414).

#### Pauciflorin H (**3**)

Colorless oil; [α]_D_^27^ + 124.5 (*c* 0.1, CHCl_3_); ^1^H and ^13^C NMR data, see Tables 1 and 2; positive-ion HRESIMS *m*/*z* 357.1693 [M + H]^+^ (calcd for C_21_H_25_O_5_^+^ 357.1697).

#### Pauciflorin I (**4**)

Colorless oil; [α]_D_^27^ + 79.3 (*c* 0.05, CHCl_3_); ^1^H and ^13^C NMR data, see Tables 1 and 2; positive-ion HRESIMS peak at *m*/*z* 373.1640 [M + H]^+^ (calcd for C_21_H_25_O_6_^+^ 373.1646).

#### Pauciflorin J (**5**)

Colorless oil; [α]_D_^27^ + 124.5 (*c* 0.1, CHCl_3_); ^1^H and ^13^C NMR data, see Tables 1 and 2; positive-ion HRESIMS peak at *m*/*z* 343.1532 [M + H]^+^ (calcd for C_20_H_23_O_5_^+^ 343.1540).

#### Pauciflorin K (**6**)

Colorless oil; [α]_D_^26^ + 6.8 (*c* 0.05, CHCl_3_); ^1^H and ^13^C NMR data, see Tables 1 and 2; positive-ion HRESIMS peak at *m*/*z* 311.1639 [M + H]^+^ (calcd for C_20_H_23_O_3_^+^ 311.1642), 333.1459 [M + Na]^+^ (calcd for C_20_H_22_O_3_Na 333.1461).

#### Pauciflorin L (**7**)

White amorphous powder; [α]_D_^26^ + 53.3 (*c* 0.05, CHCl_3_); ^1^H and ^13^C NMR data, see Tables 1 and 2; positive-ion HRESIMS *m*/*z* 355.1534 [M + H]^+^ (calcd for C_21_H_23_O_5_^+^ 355.1540).

#### Pauciflorin M (**8**)

Colorless oil; [α]_D_^25^ + 10.2 (*c* 0.1, CHCl_3_); ^1^H and ^13^C NMR data, see Tables 1 and 2; positive-ion HRESIMS peak at *m*/*z* 373.1650 [M + H]^+^ (calcd for C_21_H_25_O_6_^+^ 373.1646).

#### Pauciflorin N (**10**)

White amorphous solid; [α]_D_^27^ – 38.3 (*c* 0.05, CHCl_3_); ^1^H and ^13^C NMR data, see Table 3; positive-ion HRESIMS peak at *m*/*z* 341.1386 [M + H]^+^ (calcd for C_20_H_21_O_5_^+^ 341.1384), 363.1204 [M + Na]^+^ (calcd for C_20_H_20_O_5_Na^+^ 363.1203).

**Table 3:** NMR Data of Compounds 10, 11 [δ ppm (*J* = Hz), CDCl_3_, 500 MHz (^1^H), and 125 MHz (^13^C)]

Position	^1^H NMR		^13^C NMR	
	**10**	**11**	**10**	**11**
2	-	-	167.8	167.1
3	-	-	69.3	72.6
4	-	-	193.1	191.6
4a	-	-	118.7	119.2
5	-	-	142.8	142.3
6	7.04, d (7.8)	7.11, d (7.8)	128.6	128.4
7	7.46, t (7.8)	7.52, t (7.8)	135.8	135.6
8	7.00, d (7.8)	7.10, d (7.8)	115.0	115.3
8a	-	-	155.9	155.8
9	2.57, s	2.60, s	22.8	21.8
1′a	4.94, d (17.3)	4.93, d (17.2)	115.8	114.9
1′b	4.71, d (10.7)	4.90, d (10.7)		
2′	5.31, dd (17.3, 10.7)	5.56, dd (17.2, 10.7)	138.6	139.9
3′	-	-	56.9	48.0
4′α	2.20, dd (14.0, 8.0)	2.45, d (16.3)	42.9	50.1
4′β	2.50, dd (14.0, 5.6)	2.70, d (16.3)		
5′	5.13, td (8.0, 5.6)	-	78.6	201.1
6′	4.33, dd (10.7, 8.0)	-	48.9	130.9
7′	2.88, dq (10.7, 7.1)	-	36.3	155.7
8′	-	2.44, s	177.5	21.7
9′	0.98, d (7.1)	1.60, s	11.2	27.6
10′	1.22, s	1.27, s	25.0	23.1

#### Pauciflorin O (**11**)

White amorphous solid; [α]_D_^26^ + 80.1 (*c* 0.1, CHCl_3_); ^1^H and ^13^C NMR data, see Table 3; positive-ion HRESIMS peak at *m*/*z* 325.1431 [M + H]^+^ (calcd for C_20_H_21_O_4_^+^ 325.1434), 347.1250 [M + Na]^+^ (calcd for C_20_H_20_O_4_Na^+^ 347.1254).

### Determination of Antiproliferative Properties

The cell culturing and evaluation of the antiproliferative effects of the isolated compounds against a panel of human cancer cell lines derived from gynecological origin were conducted using a methodology described previously.^20^

## Results and Discussion

**Figure 1:**
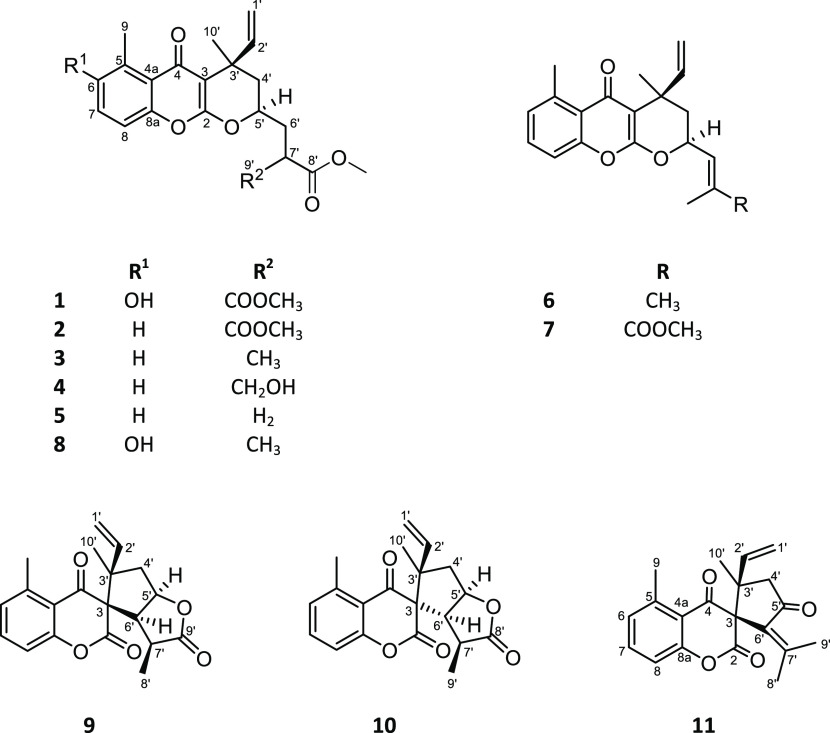
Structures of the Compounds Isolated from *C*. *pauciflorus*.

The fraction obtained from the chloroform leaf extract of *C*. *pauciflorus* with antiproliferative activity was selected for detailed phytochemical investigation and subjected to multistep chromatographic purification. Through this process, 11 compounds (**1**–**11**) (Figure 1) were isolated in pure form, and their structures were determined using spectroscopic analysis, including HRESIMS, 1D [^1^H and ^13^C *J*-modulated spin–echo (JMOD)] and 2D (^1^H–^1^H COSY, HSQC, HMBC, and NOESY) NMR experiments.

### Structure elucidation

**Figure 2:**
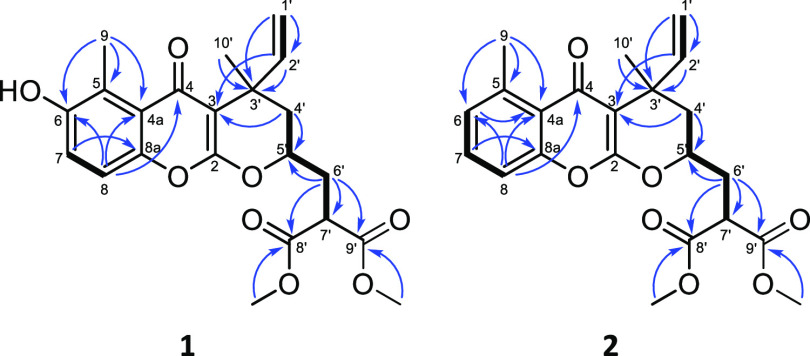
Key HMBC Correlations of Compounds **1** and **2**.

Pauciflorin F (**1**) was isolated as a colorless oily substance with an optical rotation of [α]_D_^27^ + 89.1 (*c* 0.1, CHCl_3_). The molecular formula of compound **1** was shown to be C_22_H_24_O_8_ based on the HRESIMS peak at *m*/*z* 417.1538 [M + H]^+^ (calcd for C_22_H_25_O_8_^+^ 417.1544). The ^1^H NMR, ^13^C NMR JMOD, and HSQC spectra of compound **1** revealed the presence of a 1,2,3,4-tetrasubstituted aromatic ring [δ_H_ 7.07 d (8.9 Hz), 7.03 d (8.9 Hz); δ_C_ 122.1, 124.0, 151.0, 120.4, 115.2, and 148.5], two tertiary methyl groups (δ_H_ 1.55 s, and 2.74 s; δ_C_ 24.9 and 12.7), two methoxy groups (δ_H_ 3.79 s, and 3.80 s; δ_C_ 2 × 53.0), and a vinyl group [δ_H_ 5.10 d (17.5 Hz), 5.07 d (10.7 Hz), 6.13 dd (17.5, 10.7 Hz); δ_C_ 111.8 and 145.6] (Tables 1 and 2). Additionally, three carbonyl functionalities were evident from the carbon resonances at δ_C_ 169.4, 169.6, and 179.7. The ^1^H–^1^H COSY spectrum revealed a sequence of correlated protons at δ_H_ 1.70 dd, 1.84 dd, 4.40 m, 2.34 m, and 3.82 dd, indicating a partial structure of – CH_2_–CH(OR)–CH_2_–CH(R)– (C-4′–C-7′). This structural unit, along with the quaternary carbons (δ_C_ 179.7, 169.6, 169.4, 162.2, 103.3, and 36.5), aromatic ring, methyl, and vinyl groups were connected using long-range heteronuclear correlations extracted from an HMBC spectrum. The correlations of H-7 (δ_H_ 7.07 d) with C-5 (δ_C_ 124.0) and C-8a (δ_C_ 148.5); H-8 (δ_H_ 7.03 d) with C-4a (δ_C_ 122.1) and C-6 (δ_C_ 151.0); H_3_-9 (δ_H_ 2.74 s) with C-4a, C-5, and C-6 provided evidence for the presence of a chromone structural part in pauciflorin F (**1**). In addition, the attachment of a C_10_ monoterpene unit to the chromone moiety at positions C-2 and C-3 was supported using the HMBC cross-peaks observed between H-2′ (δ_H_ 6.13 dd), H-4′ (δ_H_ 1.84 dd and 1.70 dd), H_3_-10′ (δ_H_ 1.55 s), and C-3 (δ_C_ 103.3) and C-3′ (δ_C_ 36.5), between H-4′, H-6′ (δ_H_ 2.34 m), and C-5′ (δ_C_ 74.2), and between H-5′ (δ_H_ 4.40 m), H-6′, and C-7′ (δ_C_ 47.9). Long-range correlations were also observed between H-6′ and methoxy groups (δ_H_ 3.79 s, 3.80 s) with C-8′ (δ_C_ 169.4) and C-9′ (δ_C_ 169.6), confirming the connection of two carboxymethyl group at C-7 (Figure 2).

**Figure 3:**
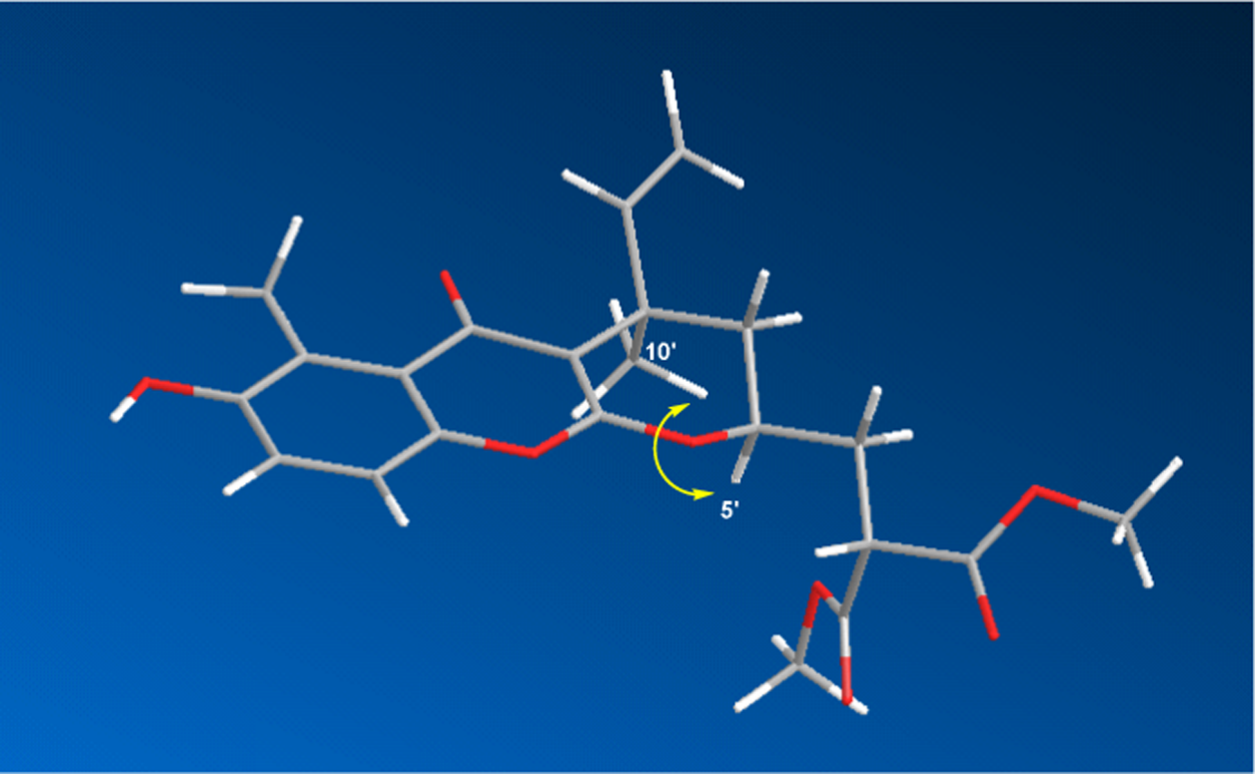
NOESY Correlation (yellow ↔) of Pauciflorin F (**1**).

The presence of hydroxyl group (δ_H_ 5.47 s) at position C-6 was deduced from the downfield shift of the aromatic carbon C-6 (δ_C_ 151.0). The chemical shift assignments of the ring system of compound **1** agreed with the published data for nassauvia chromones.^13^ Additionally, the relative configuration of compound **1** was determined using the NOESY experiment. The strong Overhauser effect observed between H_3_-10′ (δ_H_ 1.55 s) and H-5′ (δ_H_ 4.40 m) indicated their same α-orientation (Figure 3), thereby confirming the complete structure of pauciflorin F (**1**), as depicted on Figure 1.

Pauciflorin G (**2**) was isolated as a colorless oily substance with an optical rotation of [α]_D_^27^ – 73.5 (*c* 0.1, CHCl_3_). The molecular formula of compound **2** was deduced from the observed peak at *m*/*z* 401.1593 [M + H]^+^ (calcd for C_22_H_25_O_7_^+^ 401.1595) in the positive-ion HRESIMS spectrum. The ^1^H and ^13^C NMR data of compound **2** revealed a chromone-coupled monoterpenoid structure with the same monoterpene moiety as compound **1** but with a different chromone moiety. Three *ortho*-coupled aromatic protons were detected at δ_H_ 7.06 d (*J* = 7.9 Hz), 7.39 t (*J* = 7.9 Hz), and 7.14 d (*J* = 7.9 Hz), corresponding to H-6, H-7, and H-8, respectively. The chemical shifts of C-5, C-6, and C-7 at δ_C_ 141.2, 128.0, and 131.7, respectively, further supported the characterization of pauciflorin G (**2**) as the 6-deoxy derivative of pauciflorin F (**1**). The HMBC (Figure 2) and NOESY correlations agreed with the proposed structure of compound **2**.

Pauciflorin H (**3**), a colorless oil with an optical rotation value of [α]_D_^27^ + 124.5 (*c* 0.1, CHCl_3_), had a molecular composition of C_21_H_25_O_5_ based on the observed peak at *m*/*z* 357.1693 [M + H]^+^ (calcd for C_21_H_25_O_5_^+^ 357.1697) in the positive-ion HRESIMS. The ^1^H and ^13^C NMR JMOD assignments of compound **3**, obtained via the analysis of the ^1^H–^1^H COSY, HSQC, and HMBC spectra, showed that chemical shifts of compound **3** were very similar to those of compound **2** with differences observed only in the resonances of the C-7′–C-9′ region of the molecule (Tables 1 and 2). The 9′-methylcarboxylate group of **2** was replaced by a methyl group [δ_H_ 1.28 d (*J* = 7.0 Hz), δ_C_ 17.3] in compound **3**, as confirmed using the HMBC correlation observed between H_3_-9′ and C-6′ (δ_C_ 38.7), C-7′ (δ_C_ 36.0), and C-8′ (δ_C_ 176.5). The key NOESY correlations were observed between H_3_-9 (δ_H_ 2.83 s) and H-6 (δ_H_ 7.06 d), as well as between H_3_-10′ (δ_H_ 1.57 s) and H-5′ (δ_H_ 4.41 m), revealing the stereostructure of compound **3**, as shown in Figure 1. However, the configuration of C-7′ could not be determined based on NMR studies.

Pauciflorin I (**4**) was isolated as a colorless oily substance with an optical rotation of [α]_D_^27^ + 79.3 (*c* 0.05, CHCl_3_). The molecular formula of compound **4** was found to be C_21_H_20_O_5_ based on the protonated molecular ion at *m*/*z* 373.1640 [M + H]^+^ (calcd for C_21_H_25_O_6_^+^ 373.1646) detected in the HRESIMS spectrum. The ^1^H and ^13^C NMR JMOD data (Tables 1 and 2) of **4** showed a similar structural pattern as that of compound **3**, with only a difference in the functionality at C-9′ (δ_C-9′_ 63.7 for **4**, and 17.3 for **3**) and a less extent in the neighboring carbons. The 9′-methyl group (δ_H_ 1.28 s) of compound **3** was replaced by a hydroxymethyl group [δ_H_ 3.89 m (2H)] in **4**. The HMBC correlation between the 9′-methylene protons and C-8′ (δ_C_ 174.8) further supported the presence of a hydroxymethyl group at C-7′.

Pauciflorin J (**5**), a colorless oily substance with an optical rotation value of [α]_D_^27^ + 124.5 (*c* 0.1, CHCl_3_), was determined to have the molecular formula of C_20_H_22_O_5_ based on the HRESIMS by the presence of a protonated molecular ion peak at *m*/*z* 343.1532 [M + H]^+^ (calcd for C_20_H_23_O_5_^+^ 343.1540). Analysis of the ^1^H NMR and ^13^C NMR JMOD spectra of **5** revealed the presence of the same dihydropyranochromone ring system, which was substituted with two methyl groups (C-9 and C-10′) and a vinyl group (C-1′–C-2′), as observed in compounds **1**–**4**. However, compound **5** exhibited the presence of a C_9_ monoterpene unit, while the C-9′ position was missing. This observation was supported by the sequence of correlated protons in the ^1^H–^1^H COSY spectrum, which indicated the structural unit of – CH_2_–CH(OR)–CH_2_–CH_2_– [δ_H_ 1.85 dd, 1.70 dd, 4.40 m, 2.09 m (2H), and 2.62 m (2H)] (C-4′–C-7′). Furthermore, the HMBC correlation of H_2_-7′ (δ_H_ 2.62 m) and methoxy group (δ_H_ 3.72 s) with C-8′ (δ_C_ 173.4) confirmed the presence of a carboxymethyl group at C-7′. The NOESY cross-peaks between H-5′ (δ_H_ 4.40 m) and H-10′ (δ_H_ s) showed the same stereochemistry of **5** as that of compounds **1**–**4**. These findings were consistent with the proposed structure of pauciflorin J (**5**), as depicted in Figure 1.

Pauciflorin K (**6**) was isolated as a colorless oily substance with an optical rotation of [α]_D_^26^ + 6.8 (*c* 0.05, CHCl_3_). The molecular formula of compound **6** was determined to be C_22_H_22_O_3_ based on the HRESIMS peak observed at *m*/*z* 311.1639 [M + H]^+^ (calcd for C_20_H_23_O_3_^+^ 311.1642). The ^1^H NMR and ^13^C NMR JMOD spectra of compound **6** exhibited similar chemical shifts of protons and carbons as those observed in compound **5**, except for C-2 (δ_C_**5**: 162.6 vs **6**: 166.0), C-5′ (δ_C_**5**: 75.7 vs **6**: 73.9), H-5′ (δ_H_**5**: 4.40 m vs **6**: 5.07 m), and the C-6′–C-9′ side chain at C-5′ (Tables 1 and 2). This side chain was identified as an isobutenyl group, as evidenced by the HMBC correlations of H-5′ (δ_H_ 5.07 m) with C-7′ (δ_C_ 140.0), as well as H_3_-8′ (δ_C_ 1.82 s) and H_3_-9′ (δ_C_ 1.79 s) with C-6′ (δ_C_ 122.2) and C-7′. The NOESY spectrum of compound **6** revealed the presence of the characteristic Overhauser effect between the α-oriented 10′-methyl and H-5′.

Pauciflorin L (**7**) was isolated as a white amorphous powder with an optical rotation of [α]_D_^26^ + 53.3 (*c* 0.05, CHCl_3_). Its molecular formula was deduced from the peak observed at *m*/*z* 355.1534 [M + H]^+^ (calcd for C_21_H_23_O_5_^+^ 355.1540) in the positive-ion HRESIMS spectrum. The 1D and 2D NMR spectra demonstrated that compound **7** shares a similar structure to compound **6**, but the C-6′–C-9′ structural part of **7** differs (Tables 1 and 2). The side chain connected at C-5′ consists of two quaternary carbons (δ_C_ 131.3, 167.6), a methine (δ_C_ 136.9, δ_H_ 6.83 dd), a methoxy (δ_C_ 52.4, δ_H_ 3.80 s), and a methyl group (δ_C_ 13.3, δ_H_ 1.97 s). The HMBC correlations of H-5′ (δ_H_ 5.19 m) with C-6′ (δ_C_ 136.9), H_3_-9′ (δ_H_ 1.97 s) with C-7′ (δ_C_ 131.3), 8-OCH_3_ (δ_H_ 3.80 s), H_3_-9′, and H-6′ (δ_H_ 6.83 dd) with C-8′ (δ_C_ 167.6) confirmed the –CH=C(CH_3_)–COOCH_3_ side chain at C-5′ in compound **7**. The relative configuration was elucidated based on the NOESY correlations observed between H-2′/H-4′β, H-4′β/H-6′, H-5′/H-4′α, H-5′/H_3_-9′, and H-5′/H_3_-10′, providing evidence for the α position of H-5′ and H_3_-10′, as well as *trans* geometry of the C-6′/C-7′ olefin group.

Pauciflorin M (**8**), a colorless oil with an optical rotation of [α]_D_^25^ + 10.2 (*c* 0.1, CHCl_3_), displayed the molecular composition C_21_H_24_O_6_ according to the peak observed at *m*/*z* 373.1650 [M + H]^+^ (calcd for C_21_H_25_O_6_^+^ 373.1646) in the positive-ion HRESIMS. The NMR characteristics of compound **8** revealed similarities to **1** regarding the aromatic part of the molecule and compound **3** regarding the monoterpene moiety. The ^1^H and ^13^C NMR chemical shift assignments, performed using the HSQC, HMBC, and ^1^H–^1^H COSY spectra, confirmed the presence of a 5-methyl-6-hydroxychromone and a monoterpene carboxylic acid methyl ester adduct structure in compound **8** (Tables 1 and 2).

Compound **9** was isolated as colorless oil and identified based on its 1D and 2D NMR spectroscopic data as (+)-spiro-ethuliacoumarin (**9**). This compound was previously isolated from *Ethulia conyzoides*.^23^ The complete structure and the relative stereochemistry of compound **9** were established by Mahmoud et al. through X-ray crystallography.

Pauciflorin N (**10**) was obtained as a white amorphous powder with an optical rotation of [α]_D_^27^ – 38.3 (*c* 0.05, CHCl_3_). The molecular formula of compound **10** was determined to be C_20_H_20_O_5_ based on the positive-ion HRESIMS peak observed at *m*/*z* 341.1386 [M + H]^+^ (calcd for C_20_H_21_O_5_^+^ 341.1384). The ^1^H and ^13^C JMOD NMR spectra of compound **10** showed a structural pattern similar to that of compound **9** (Table 3).

In the ^1^H–^1^H COSY spectrum, identical spin systems were identified for both compounds, concluding that compounds **9** and **10** are stereoisomers. Notable differences in chemical shifts were observed in their ^1^H and ^13^C NMR spectra in the carbon resonances around the spiro C-3 stereocenter (C-3, C-4, C-1′, C-4′, C-6′, and C-10′) and the corresponding proton resonances (H-1′*cis*, H-2′, H-4′β, H-6′, and H_3_-10′). The NOESY correlations of **10** between H_3_-10′ and H-5′, H-6′, H-4′α, between H-6′ and H-7′, and between H-2′ and H-4′β agreed those published for compound **9**.^21^ These correlations confirmed the α-position of H_3_-10′, H-5′, and H-6′, as well as β-position of vinyl and 9′-methyl groups. The only possible difference in compounds **9** and **10** is likely the opposite stereochemistry of C-3. This is supported by the considerable difference in the chemical shifts of the 10′-methyl group (δ_H_**9**: 1.01 s vs **10**: 1.22 s) and the key NOESY correlation between H_3_-9 and H_3_-10′. Such correlation is only possible when the α-oriented H_3_-10′ methyl group is connected to the spiro-structure opposite to that of compound **9**. In the 3D model of **10**, the distance between H_3_-9 and H_3_-10′ protons was 2.9 Å (Figure S73). Consequently, the structure of pauciflorin N was elucidated, as presented in the structural formula of compound **10**.

Pauciflorin O (**11**) was isolated as a white amorphous solid material with an optical rotation value of [α]_D_^26^ + 80.1 (*c* 0.1, CHCl_3_). Its molecular formula was found to be C_20_H_20_O_4_ from the protonated molecular ion observed at *m*/*z* 325.1431 [M + H]^+^ (calcd for C_20_H_21_O_4_^+^ 325.1434) detected in the MS spectrum. HRESIMS, ^1^H and ^13^C NMR JMOD data indicated that the molecule has 11 degrees of unsaturation. In the ^13^C NMR spectrum, the presence of two keto groups (δ_C_ 191.6 and 201.1) and one ester functionality (δ_C_ 167.1) were detected beside an aromatic nucleus and two other double bonds—one monosubstituted (δ_C_ 114.9, 139.9) and the other tetrasubstituted (δ_C_ 130.9, 155.7). These structural elements contribute to nine unsaturations, which indicates the presence of two additional rings in the molecule: the 2,4-chromandione part and another ring in the terpene segment. The ^1^H–^1^H COSY spectrum showed two spin systems, namely CH_2_=CH– (δ_H_ 4.90, 4.93, and 5.56 dd) (C-1′–C-2”) and –CH=CH–CH= (δ_H_ 7.11, 7.52, and 7.10 d) (C-6–C-8). The planar structure of compound **11** was constructed using the HMBC correlations. Noteworthy correlations were observed between C-3/H_3_-10′, C-3/H_2_-4′, C-4/H-6, C-4/H_3_-9, C-8a/H-7, C-8a/H-8, C-6′/H_3_-8′, C-6′/H_3_-9′, C-6′/H_2_-4′, C-5′/H_2_-4′, and C-5′/H_3_-9′. However, the characteristic NOESY correlation between the H_3_-9 and H_3_-10′ characteristic to the spiro-structure of **10** was absent in the case of **11**. Additionally, a comparison of ^13^C NMR chemical shifts of C-2–C-4 and C-10′ of compounds **9**, **10**, and **11** was also conducted to determine the relative configuration. The chemical shifts of C-2 (δ_C_**9**: 167.4, **10**: 167.8, **11**: 167.1), C-3 (δ_C_**9**: 70.8, **10**: 69.3, **11**: 72.6), C-4 (δ_C_**9**: 191.0, **10**: 193.1, **11**: 191.6), and C-10′ (δ_C_**9**: 23.1 **10**: 25.0, **11**: 23.1) of **11** were more similar to those of compound **9**, proving the same stereochemistry of C-3 in pauciflorin O (**11**).

### Assay for Antiproliferative Activity

Seven of the isolated compounds, namely pauciflorins F, G, H, J, M, and O (**1**–**3**, **5**, **8**, **11**) and (+)-spiro-ethuliacoumarin (**9**), were investigated for their potential antiproliferative activity against a panel of adherent human malignant cell lines comprising breast (MCF-7, MDA-MB-231), cervical (HeLa, SiHa), and ovarian (A2780) cancer cells. In the first MTT (3-(4,5-dimethylthiazol-2-yl)-2,5-diphenyltetrazolium bromide) test, two final concentrations (10 and 30 μM) were tested after 72 h of incubation. Subsequently, a broad range of concentrations (0.1–30 μM) was employed when at least 50% cell growth inhibition was detected at 30 μM. Among the compounds tested, only pauciflorin F (**1**) elicited >50% inhibition of proliferation against MCF-7 and A2780 cells. Notably, its activity against MCF-7 was comparable to that of the reference agent cisplatin, as evidenced by the IC_50_ values (Table 4). However, pauciflorin F (**1**) was ineffective against HeLa cells and exhibited modest activity on MDA-MB-231 and SiHa cells. Pauciflorins G, H, J, and M (**2**, **3**, **5**, and **8**) showed weak antiproliferative effects, with maximal inhibition of 40%–50% on some cell lines. Conversely, (+)-spiro-ethuliacoumarin (**9**) and pauciflorin O (**11**) demonstrated limited efficacy, eliciting <35% growth inhibition against the tested cell lines.

**Table 4:** Antiproliferative Properties of the Isolated Compounds 1–3, 5, 8, 9, and 11

		Inhibition (%) ± SEM and calculated IC_50_ (μM)				
Compound	Conc. (μM)	MCF-7	MDA-MB-231	HeLa	SiHa	A2780
**1**	10	40.47 ± 2.18	22.79 ± 0.80	–a	–	–
	30	84.14 ± 0.45	47.32 ± 1.44	–	36.75 ± 1.63	66.23 ± 0.66
	IC_50_	**11.74**	–b	–b	–b	**28.37**
**2**	10	28.02 ± 1.87	–	–	26.76 ± 1.05	–
	30	38.50 ± 2.18	27.37 ± 1.92	30.53 ± 1.13	41.53 ± 0.73	24.34 ± 3.72
**3**	10	–	–	–	–	–
	30	25.36 ± 3.73	–	40.61 ± 2.33	27.70 ± 1.41	–
**5**	10	34.65 ± 1.47	22.87 ± 2.64	39.32 ± 3.34	32.71 ± 2.21	22.52 ± 1.31
	30	44.90 ± 2.17	23.67 ± 2.71	41.26 ± 3.72	46.57 ± 0.32	23.49 ± 2.06
**8**	10	–	22.26 ± 1.10	31.60 ± 3.01	–	20.38 ± 1.73
	30	22.88 ± 2.40	24.87 ± 0.56	44.62 ± 1.22	21.51 ± 1.96	41.00 ± 2.75
**9**	10	–	–	–	–	–
	30	–	–	23.71 ± 0.58	–	–
**11**	10	22.43 ± 0.81	–	–	20.62 ± 1.61	–
	30	34.92 ± 0.85	–	22.51 ± 1.42	30.77 ± 2.35	21.80 ± 1.11
cisplatin^c^	10	66.91 ± 1.81	42.72 ± 2.68	42.61 ± 2.33	60.98 ± 0.92	83.57 ± 2.21
	30	96.80 ± 0.35	86.44 ± 0.42	99.93 ± 0.26	88.95 ± 0.53	95.02 ± 0.28
	IC_50_	5.78	10.17	12.43	4.29	1.30

a Inhibition values lower than 20% are considered negligible and not given numerically.

b Not determined.

c Results from ref (20).

## Conclusion

The chloroform extract of the methanol extract prepared from *C*. *pauciflorus* leaves was subjected to an activity-guided isolation process to identify compounds with potential antiproliferative activity. The chloroform extract and its fractions were tested against the human breast (MCF-7 and MDA-MB-231), cervical (HeLa and SiHa), and ovarian (A2780) cancer cell lines and fractions with high activity were further purified using multistep chromatographic separations. Ten previously undescribed meroterpenoids (**1**–**8**, **10**, **11**), named pauciflorins F–O, and the known compound (+)-spiro-ethuliacoumarin were isolated, and their structures determined using MS and NMR measurements. These compounds represent three structural types: 5-methylchromone–monoterpene (**1**–**8**), tricyclic 5-methyl-2,4-chromadione–monoterpene (**11**), and tetracyclic 5-methyl-2,4-chromadione–monoterpene derivatives (**9**, **10**). The 2,4-chromadione-based meroterpenoids have a spiro-structure. A common structural feature among these compounds is the presence of a 5-methyl group at C-5, as well as methyl and vinyl groups at C-3′. The structural variations originate from the monoterpene part, which can have lactone, carboxymethyl, hydroxymethyl, methyl, or olefin functionalities. Pauciflorin J (**5**) is the only compound featuring a C_9_ nor-monoterpene moiety.

The occurrence of chromone-monoterpene-based meroterpenoids in plants is sparely reported in the literature. Gerdelavin B was isolated from the Chinese Asteraceae species *Gerbera delavayi*,^11^ and additional 5-methyl-chromone–monoterpene adducts were obtained from *Gerbera piloselloides*.^10^ Ptaerobliquol, belonging to the same structural type, was found in *Ptaeroxylon obliquum* (Rutaceae).^24^ Nassauvia chromones, similar to pauciflorins F–M (**1**–**8**), were obtained from *Triptilion spinosum*, featuring a tricyclic, 5-methylchromone-containing ring system with methyl and vinyl groups in position C-3′, but with a long aliphatic chain attached at C-5′.^14^

5-Methyl-2,4-chromadione–monoterpenes are rare in nature. Previously, only the isolation of (+)-spiro-ethuliacoumarin (**9**) had been reported from the Egyptian plant *Ethulia conyzoides*.^23^ In addition, a compound encoded as ZINC31161132 with a 5-methyl-2,4-chromadione–monoterpene structure, was virtually screened for antituberculosis activity using a pharmacophor model.^25^

**Figure 4:**
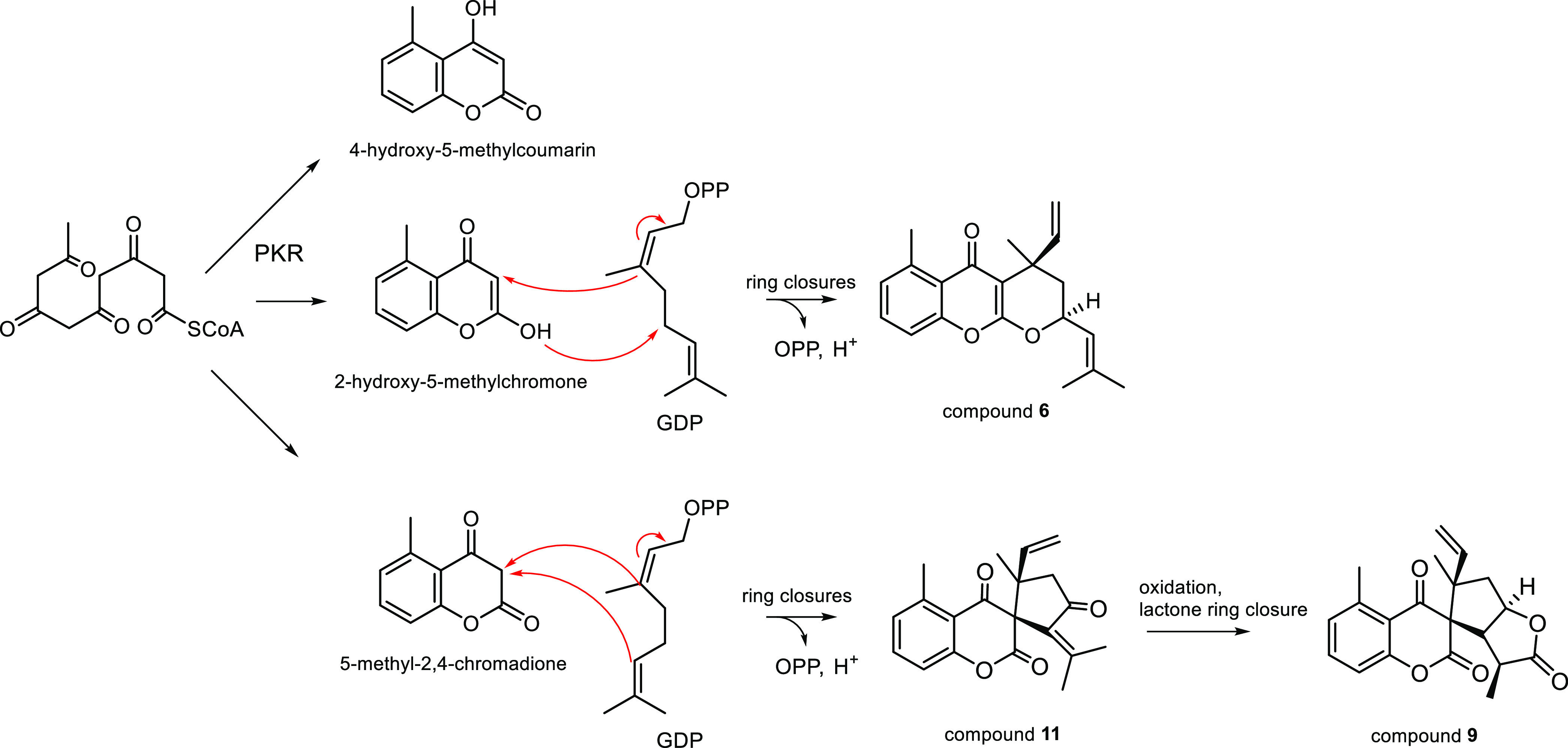
Putative Biogenetic Pathway Proposed for the Main Structural Types Represented by Compounds **6**, **9** and **11**. PKR: polyketide reductase; GDP: geranyl-diphosphate

As regards, the biosynthesis of the compounds, a common biosynthetic origin can be supposed for the co-occurring 4-hydroxy-5-methylcoumarin, 2-hydroxcy-5-methylchromone, and 5-methyl-2,4-chromadione derivatives (Figure 4). The aromatic parts are derived though the acetate-malonate pathway,^17^ while the monoterpene parts form from geranyl-diphosphate (GDP). Claisen cyclization of the polyketide precursor affords the aromatic rings by enzymatic route catalyzed by polyketide reductase (PKR), and *O*-heterocyclic rings are formed in the next steps (enolization, Michael-type nucleophilic attack, etc.). The connection of the aromatic parts with GDP includes C-alkylation, oxidative clavage, cyclization and lactonization.^26^Figure 4 shows the putative biogenetic pathway proposed for the main structural types of the isolated compounds represented by **6**, **9** and **11**.

Seven isolated compounds were assayed for antiproliferative action against human adherent cancer cell lines of gynecological origin using the MTT method. Pauciflorin F (**1**) exhibited considerable activity against MCF-7 breast cancer cells, with an IC_50_ value comparable to that of the clinically used drug cisplatin. However, its activity was less pronounced against ovarian (A2780) and triple-negative breast (MDA-MB-231), while no relevant effect was detected on cervical cancer cell lines (HeLa and SiHa). Despite sharing structural similarities, the remaining investigated compounds, namely pauciflorins G, H, J, M, and O (**2**, **3**, **5**, **8**, and **11**) and (+)-spiro-ethuliacoumarin (**9**), did not exhibit substantial activity against the tested cancer cell lines.

## Supporting Information Available

HRESIMS, NMR spectra of **1**–**11** and 3D structure of compound **10** (PDF)
