# Supplementary material for: Anticancer Meroterpenoids from Centrapalus pauciflorus leaves: Chromone- and 2,4-Chromadione-Monoterpene Derivatives
Source: ACS Omega. 2023 Aug 16;8(34):31389–98. doi: 10.1021/acsomega.3c03884 (PMC10468835; doi:10.1021/acsomega.3c03884)
Supplement: Supplementary file 1 — ao3c03884_si_001.pdf [file ao3c03884_si_001.pdf]

SUPPORTING INFORMATION

Anticancer Meroterpenoids from *Centrapalus pauciflorus* leaves: Chromone- and 2,4-Chromadione-Monoterpene Derivatives

Gordana Krstić,[1,2,#] Muhammad Bello Saidu,[1,#] Anita Barta,[1] Máté Vágvölgyi,[1] Hazhmat Ali,[3] István Zupkó,[3] Róbert Berkecz,[4] Umar Shehu Gallah,[5] Dóra Rédei,[1]* Judit Hohmann[1,6]*

[1] Department of Pharmacognosy, University of Szeged, Eötvös u. 6, 6720 Szeged, Hungary

[2] University of Belgrade - Faculty of Chemistry, Studentski trg 12-16, 11158 Belgrade, Serbia

[3] Institute of Pharmacodynamics and Biopharmacy, University of Szeged, Eötvös u. 6, 6720 Szeged, Hungary

[4] Institute of Pharmaceutical Analysis, University of Szeged, Somogyi u. 4, 6720 Szeged, Hungary

[5] Bioresource Department, National Research Institute for Chemical Technology (NARICT), Zaria, Nigeria

[6] ELKH-USZ Biologically Active Natural Products Research Group, University of Szeged, Eötvös u. 6, H-6720 Szeged, Hungary

CONTENT

Figure S1. ^{1}H NMR spectrum of **1** 3
Figure S2. ^{13}C NMR JMOD spectrum of **1** 3
Figure S3. HSQC spectrum of **1** 4
Figure S4. HMBC spectrum of **1** 4
Figure S5. COSY spectrum of **1** 5
Figure S6. NOESY spectrum of **1** 5
Figure S7. HRESIMS spectrum of **1** 6
Figure S8. ^{1}H NMR spectrum of **2** 6
Figure S9. ^{13}C NMR JMOD spectrum of **2** 7
Figure S10. HSQC spectrum of **2** 7
Figure S11. HMBC spectrum of **2** 8
Figure S12. COSY spectrum of **2** 8
Figure S13. NOESY spectrum of **2** 9
Figure S14. HRESIMS spectrum of **2** 9
Figure S15. ^{1}H NMR spectrum of **3** 10
Figure S16. ^{13}C NMR JMOD spectrum of **3** 10
Figure S17. HSQC spectrum of **3** 11
Figure S18. HMBC spectrum of **3** 11
Figure S19. COSY spectrum of **3** 12
Figure S20. NOESY spectrum of **3** 12
Figure S21. HRESIMS spectrum of **3** 13
Figure S22. ^{1}H NMR spectrum of **4** 13
Figure S23. ^{13}C NMR JMOD spectrum of **4** 14
Figure S24. HSQC spectrum of **4** 14
Figure S25. HMBC spectrum of **4** 15
Figure S26. COSY spectrum of **4** 15
Figure S27. NOESY spectrum of **4** 16
Figure S28. HRESIMS spectrum of **4** 16
Figure S29. ^{1}H NMR spectrum of **5** 17
Figure S30. ^{13}C NMR JMOD spectrum of **5** 17
Figure S31. HSQC spectrum of **5** 18
Figure S32. HMBC spectrum of **5** 18
Figure S33. COSY spectrum of **5** 19
Figure S34. NOESY spectrum of **5** 19
Figure S35. HRESIMS spectrum of **5** 20
Figure S36. ^{1}H NMR spectrum of **6** 20
Figure S37. ^{13}C NMR JMOD spectrum of **6** 21
Figure S38. HSQC spectrum of **6** 21
Figure S39. HMBC spectrum of **6** 22
Figure S40. COSY spectrum of **6** 22
Figure S41. NOESY spectrum of **6** 23
Figure S42. HRESIMS spectrum of **6** 23
Figure S43. ^{1}H NMR spectrum of **7** 24
Figure S44. ^{13}C NMR JMOD spectrum of **7** 24
Figure S45. HSQC spectrum of **7** 25
Figure S46. HMBC spectrum of **7** 25
Figure S47. COSY spectrum of **7** 26
Figure S48. NOESY spectrum of **7** 26
Figure S49. HRESIMS spectrum of **7** 27
Figure S50. ^{1}H NMR spectrum of **8** 27

Figure S51. ^{13}C NMR JMOD spectrum of **8** 28
Figure S52. HSQC spectrum of **8** 28
Figure S53. HMBC spectrum of **8** 29
Figure S54. COSY spectrum of **8** 29
Figure S55. NOESY spectrum of **8** 30
Figure S56. HRESIMS spectrum of **8** 30
Figure S57. ^{1}H NMR spectrum of **9** 31
Figure S58. ^{13}C NMR JMOD spectrum of **9** 31
Figure S59. ^{1}H NMR spectrum of **10** 32
Figure S60. ^{13}C NMR JMOD spectrum of **10** 32
Figure S61. HSQC spectrum of **10** 33
Figure S62. HMBC spectrum of **10** 33
Figure S63. COSY spectrum of **10** 34
Figure S64. NOESY spectrum of **10** 34
Figure S65. HRESIMS spectrum of **10** 35
Figure S66. ^{1}H NMR spectrum of **11** 35
Figure S67. ^{13}C NMR JMOD spectrum of **11** 36
Figure S68. HSQC spectrum of **11** 36
Figure S69. HMBC spectrum of **11** 37
Figure S70. COSY spectrum of **11** 37
Figure S71. NOESY spectrum of **11** 38
Figure S72. HRESIMS spectrum of **11** 38
Figure S73. 3D structure of compound **10** 39

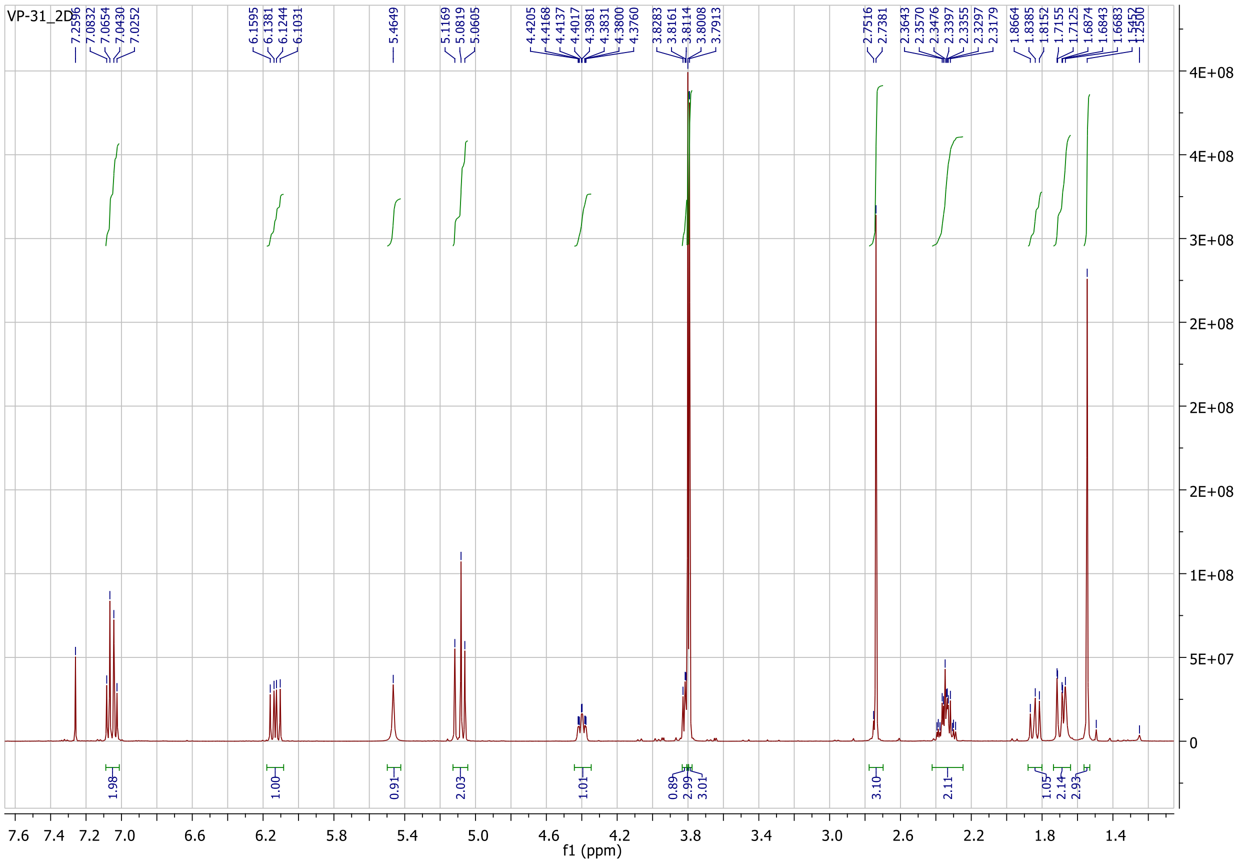

Figure S1. ^{1}H NMR spectrum of **1** ($CDCl_3$, 500 MHz)

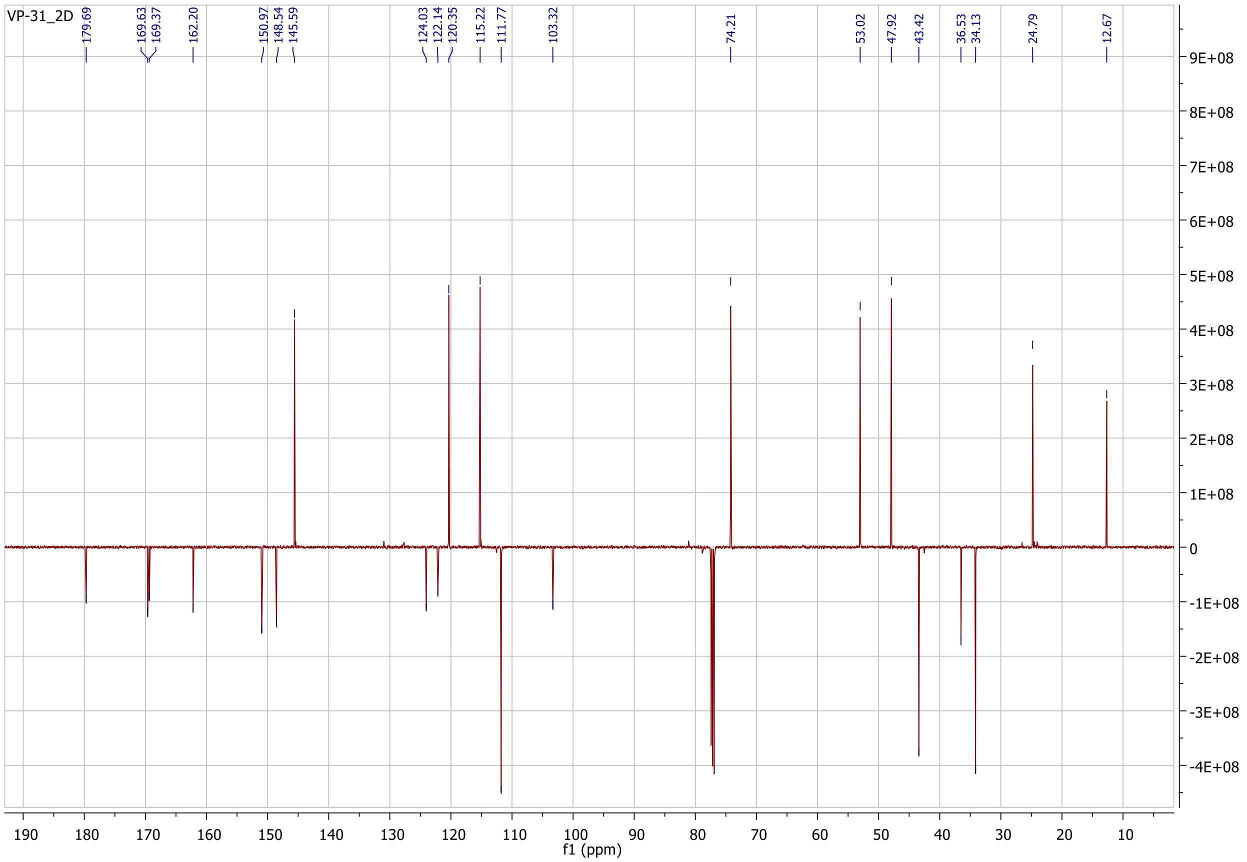

Figure S2. ^{13}C NMR JMOD spectrum of **1** ($CDCl_3$, 125 MHz)

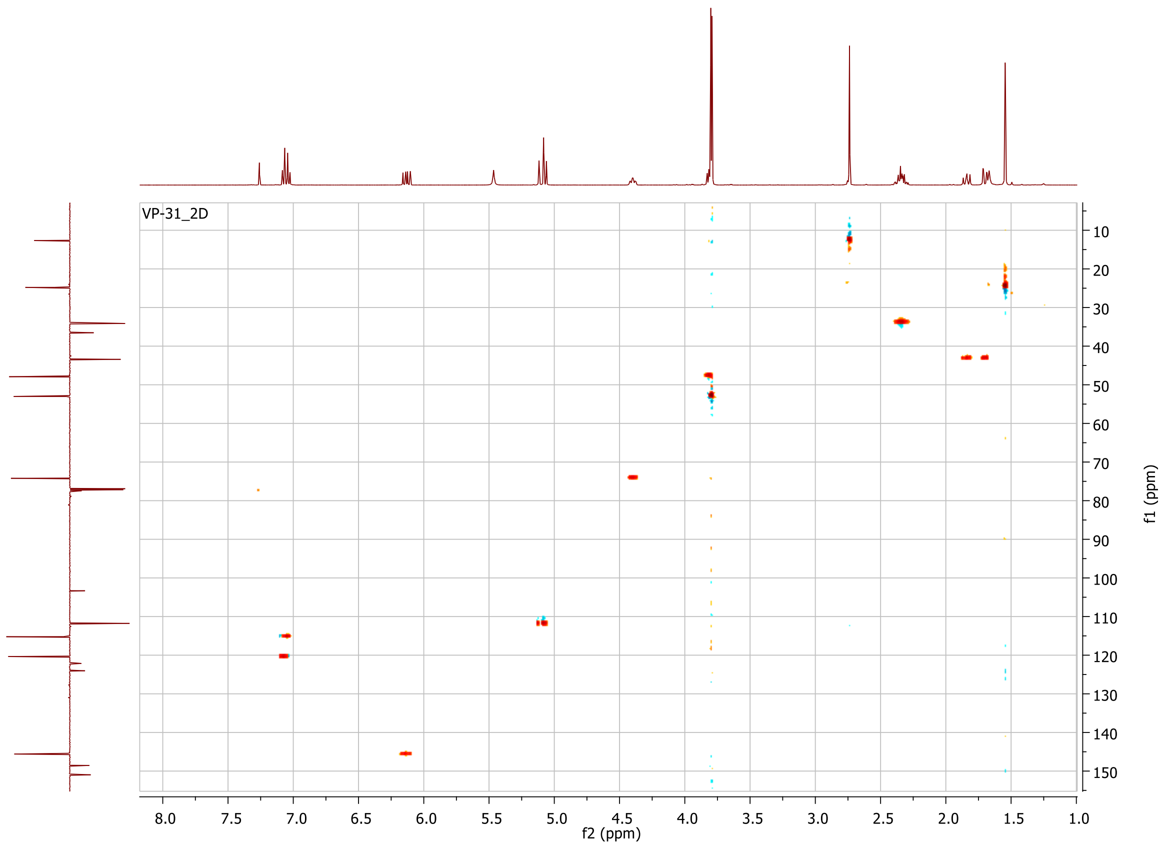

Figure S3. HSQC NMR spectrum of **1** ($CDCl_3$, 125/500 MHz)

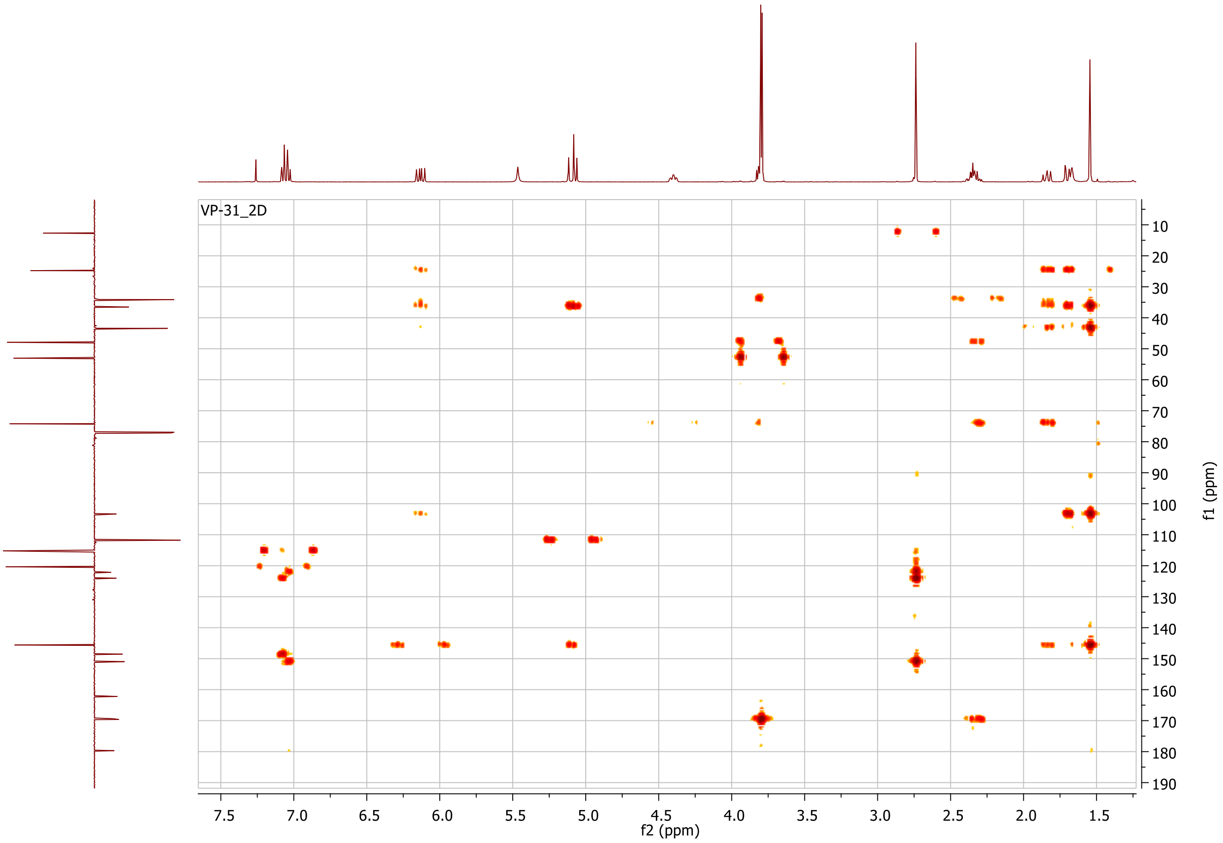

Figure S4. HMBC NMR spectrum of **1** ($CDCl_3$, 125/500 MHz)

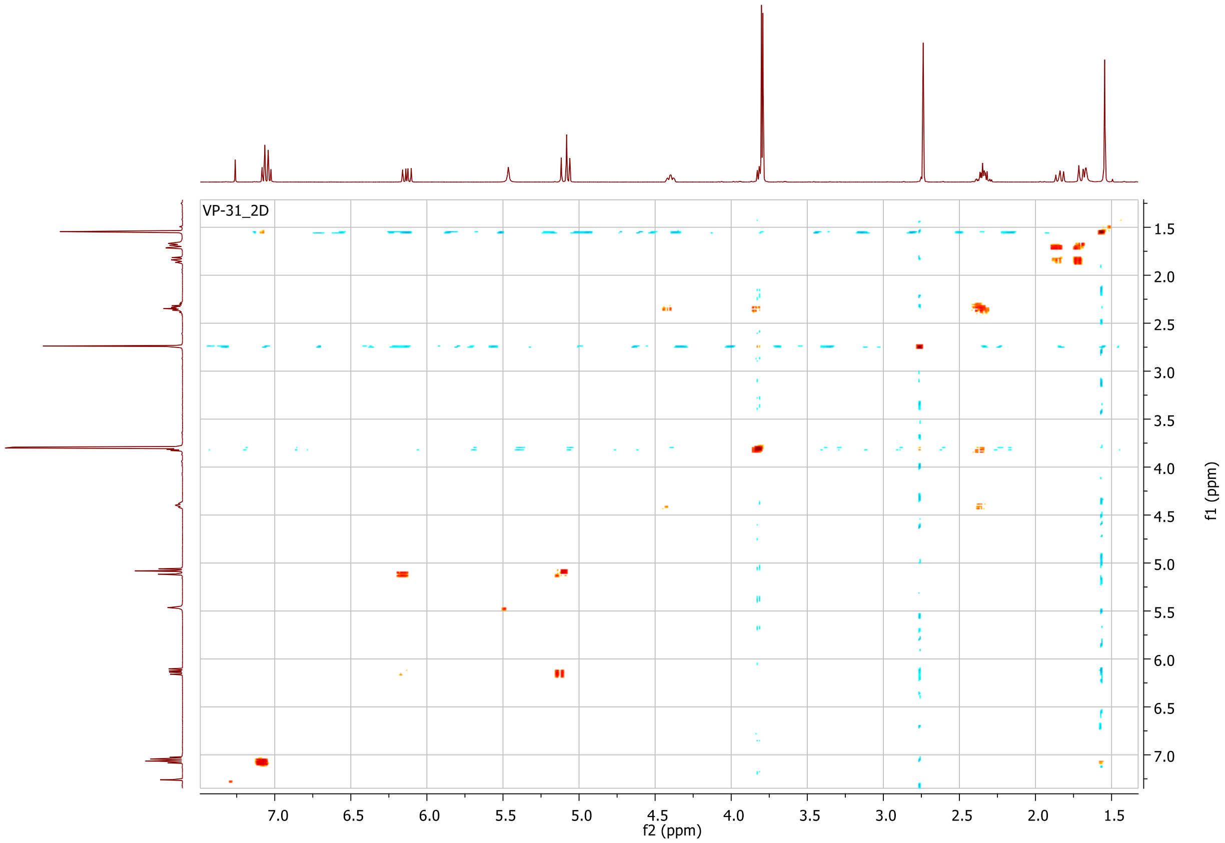

Figure S5. COSY NMR spectrum of **1** ($CDCl_3$, 500 MHz)

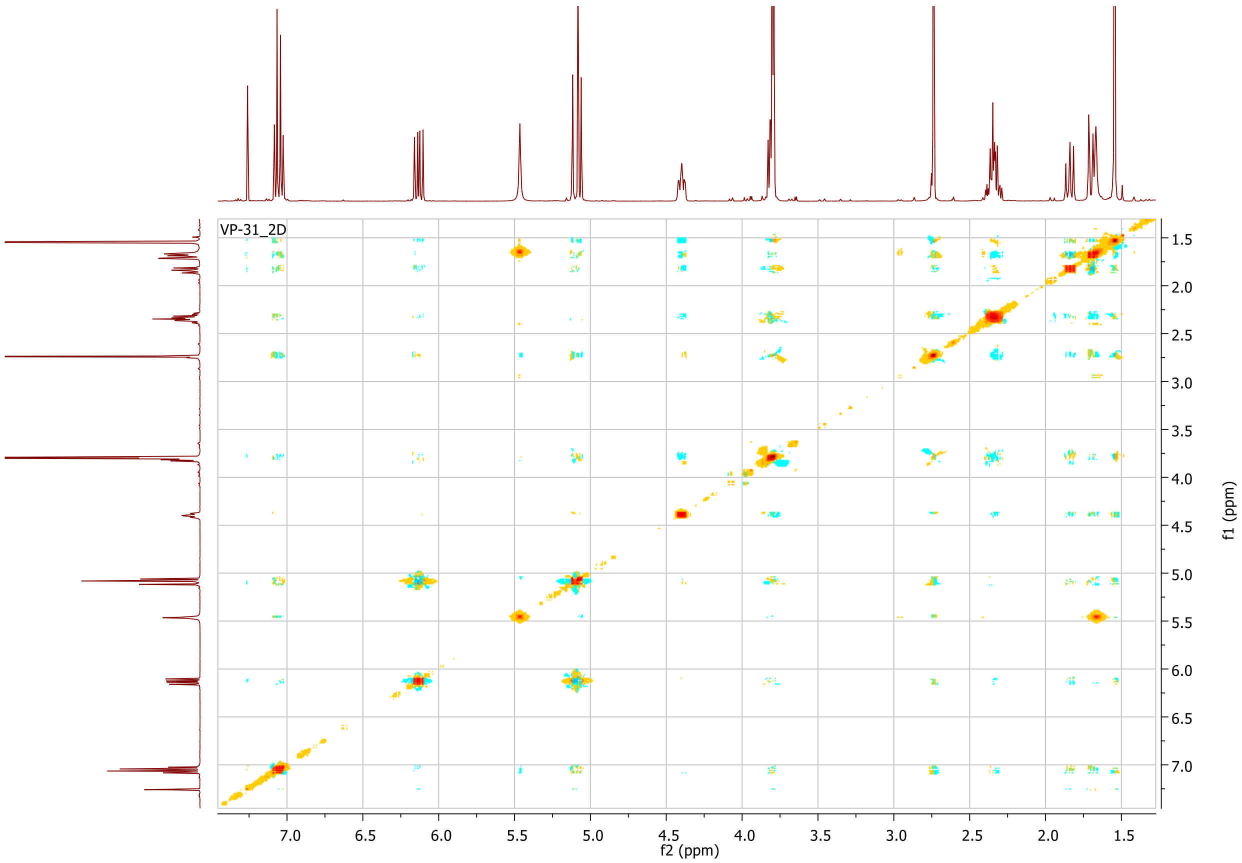

Figure S6. NOESY spectrum of **1** ($CDCl_3$, 500 MHz)

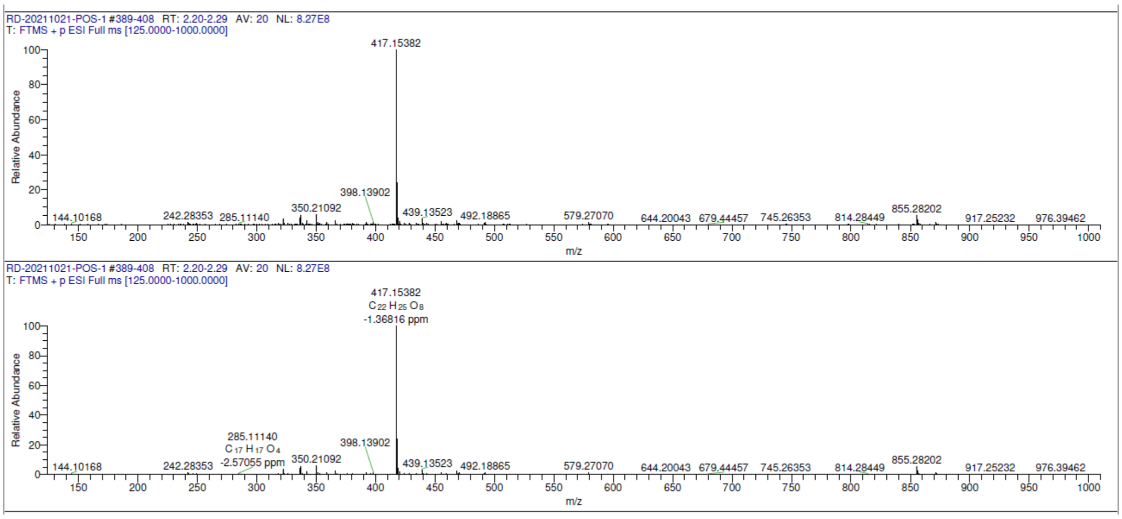

Figure S7. HRESIMS spectrum of **1**

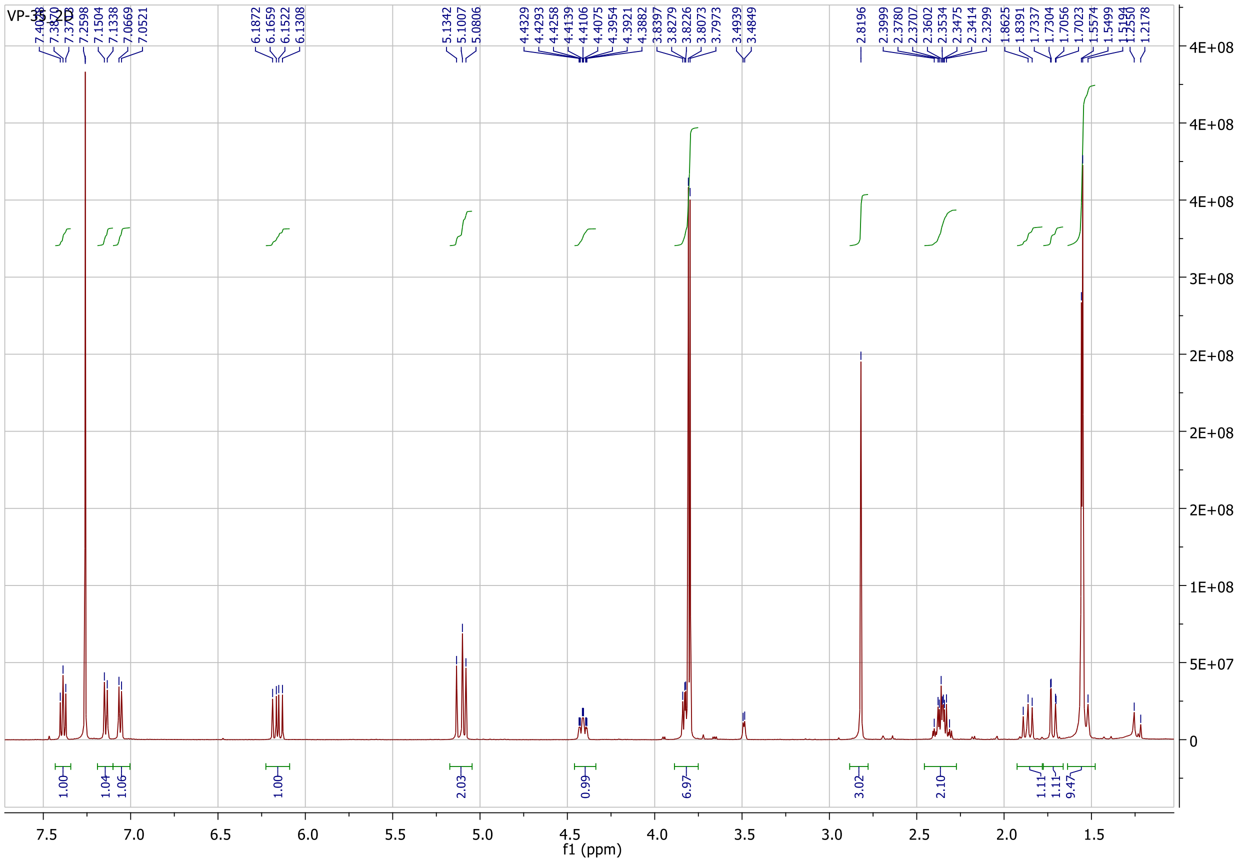

Figure S8. ^{1}H NMR spectrum of **2** ($CDCl_3$, 500 MHz)

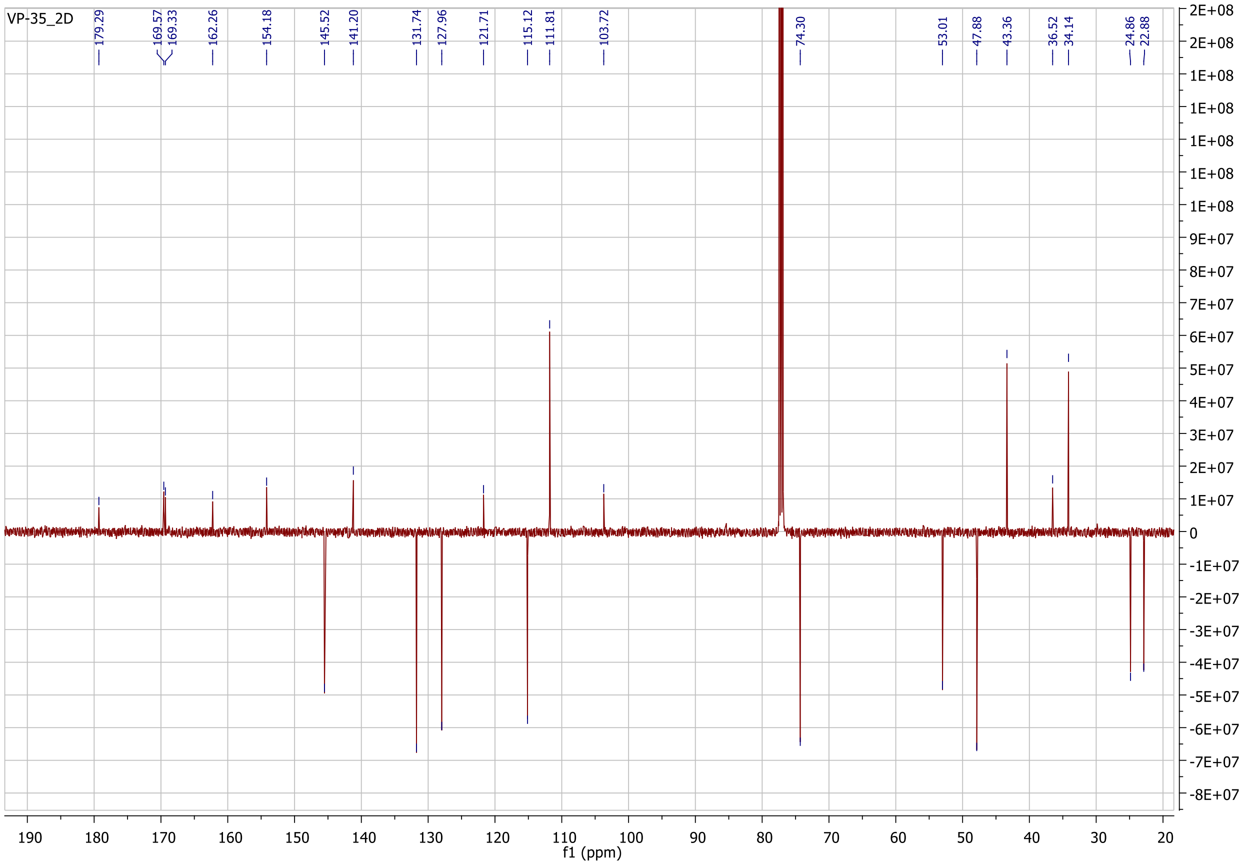

Figure S9. ^{13}C NMR JMOD spectrum of **2** ($CDCl_3$, 125 MHz)

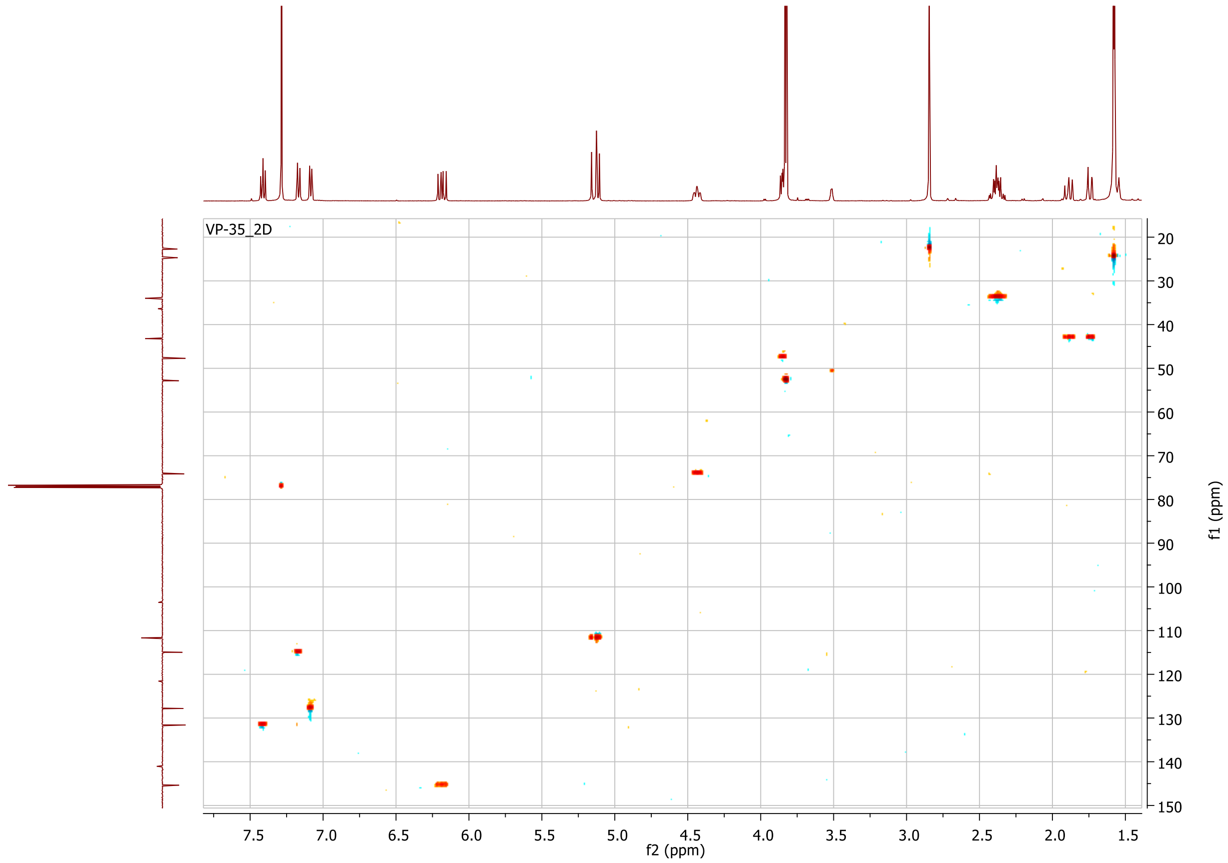

Figure S10. HSQC spectrum of **2** ($CDCl_3$, 125/500 MHz)

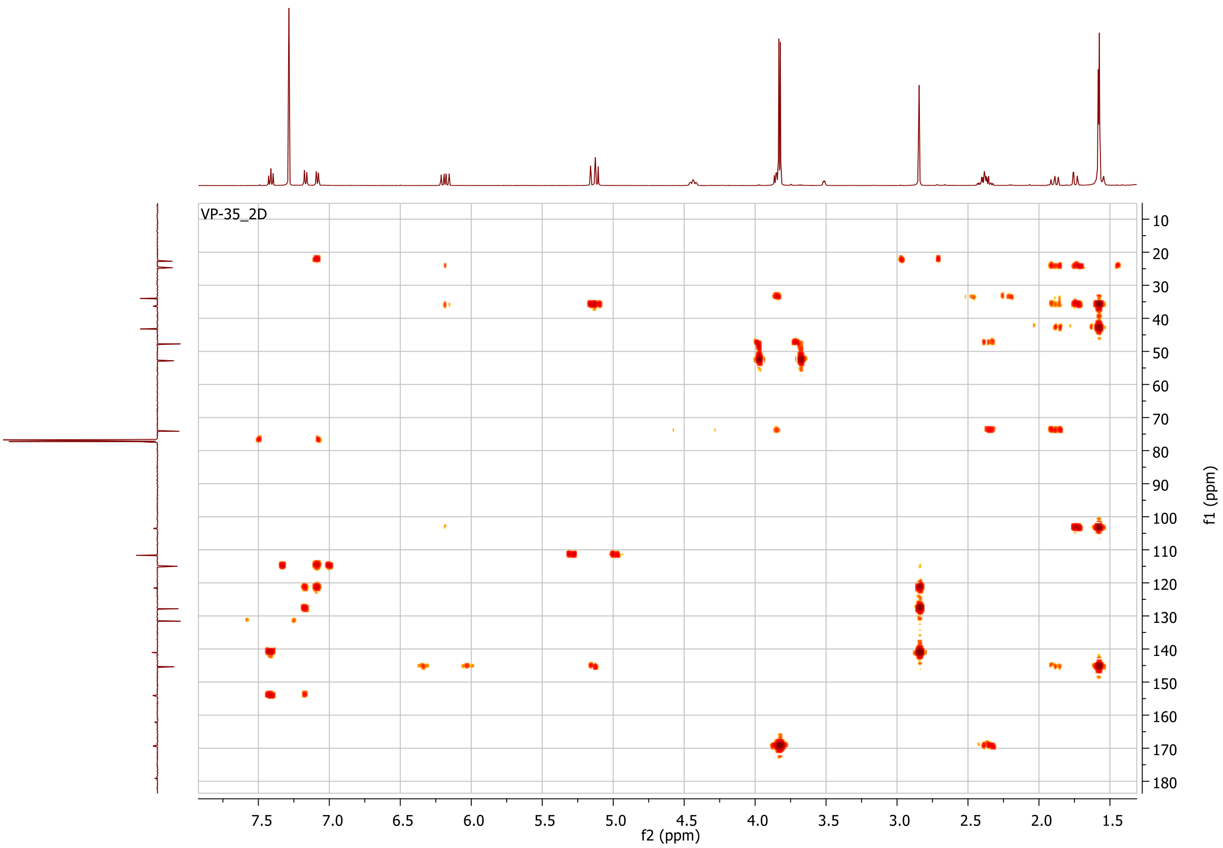

Figure S11. HMBC spectrum of **2** ($CDCl_3$, 125/500 MHz)

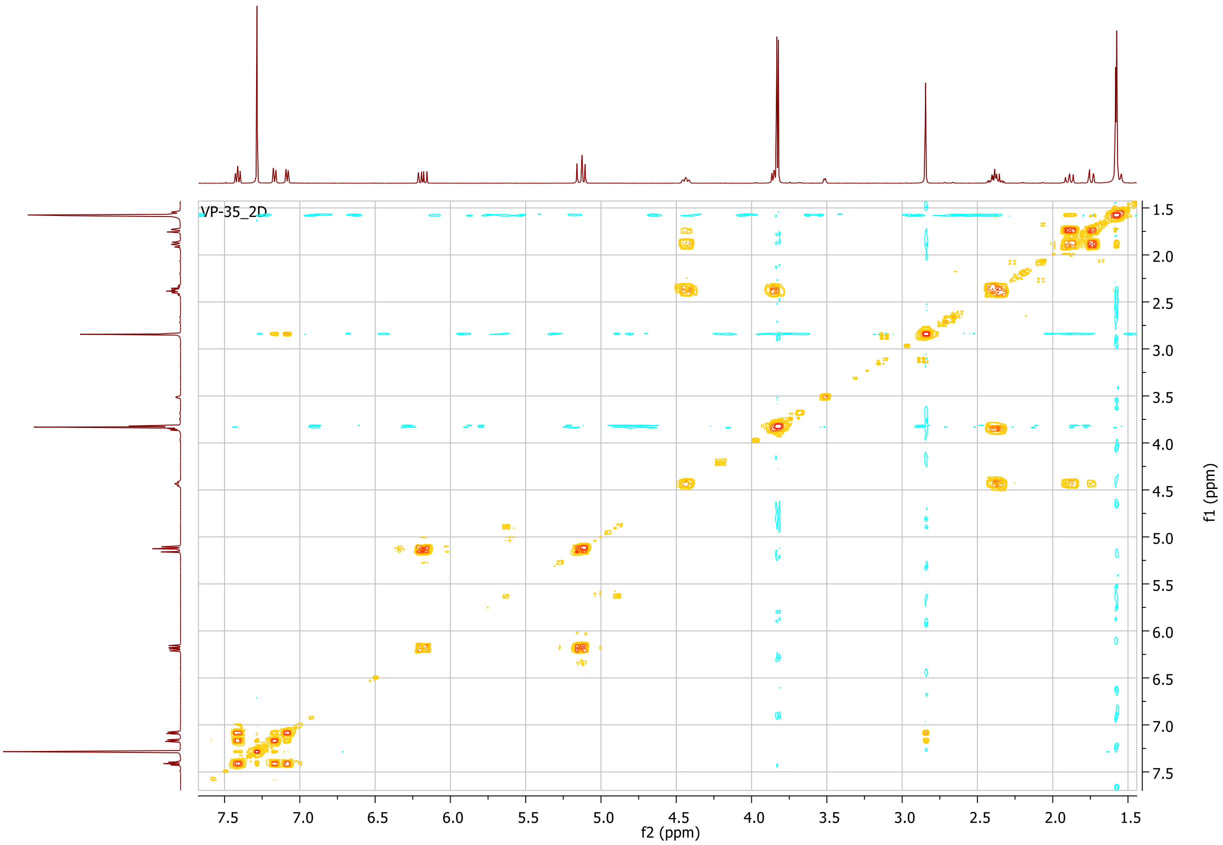

Figure S12. COSY spectrum of **2** ($CDCl_3$, 500 MHz)

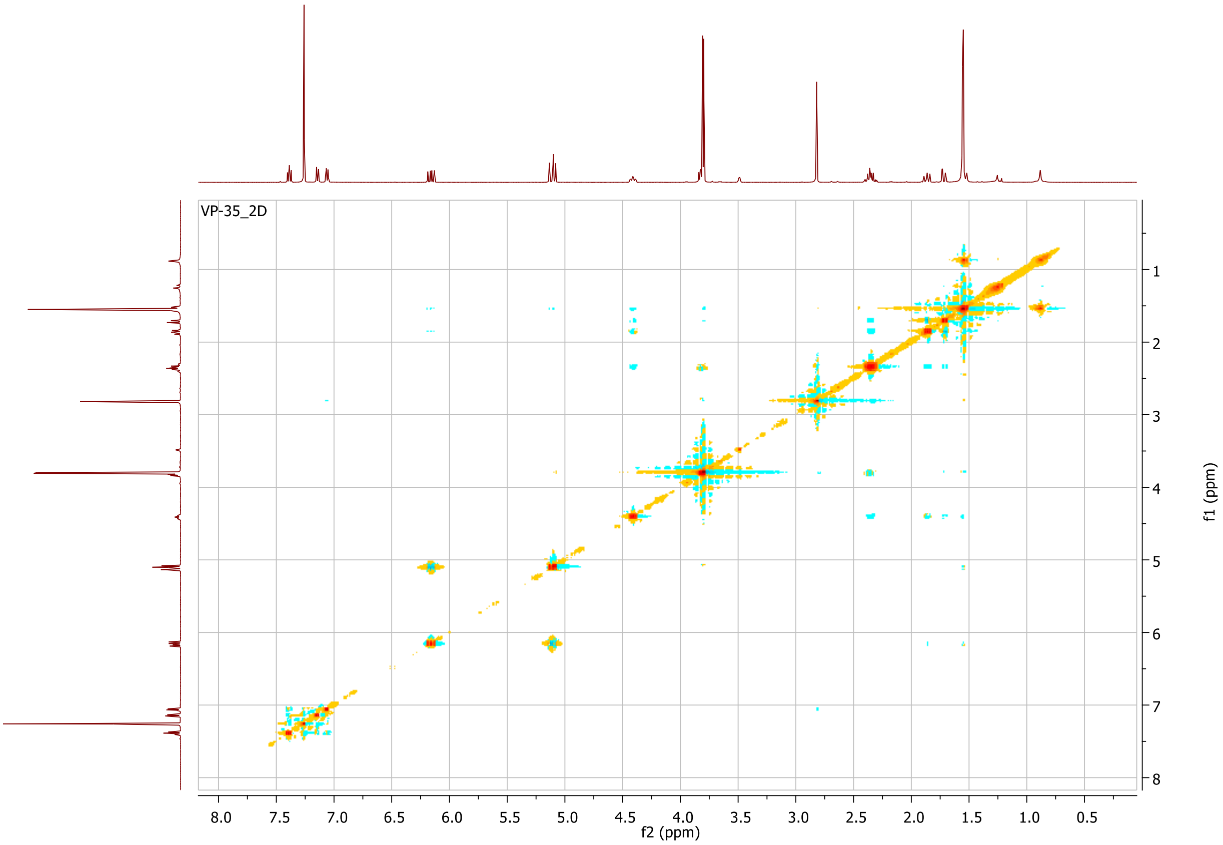

Figure S13. NOESY spectrum of **2** ($CDCl_3$, 500 MHz)

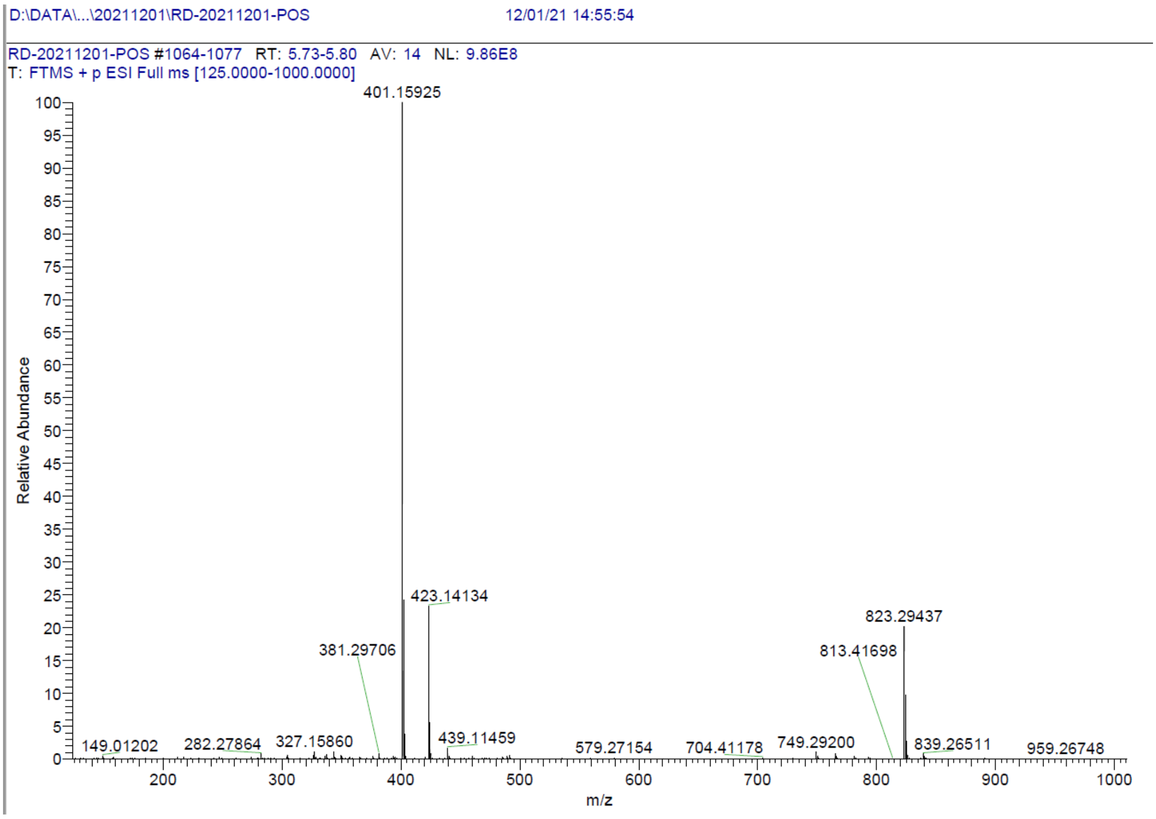

Figure S14. HRESIMS spectrum of **2**

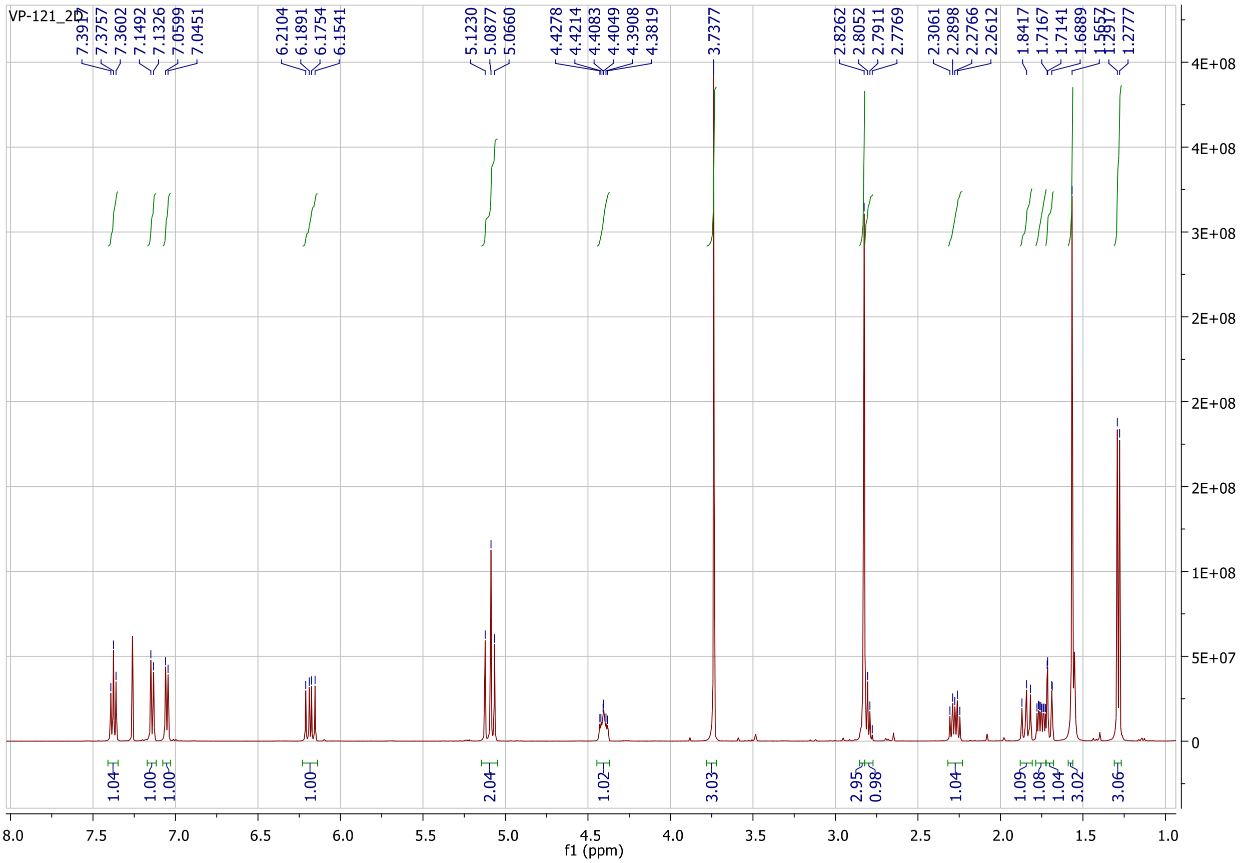

Figure S15. ^{1}H NMR spectrum of **3** ($CDCl_3$, 500 MHz)

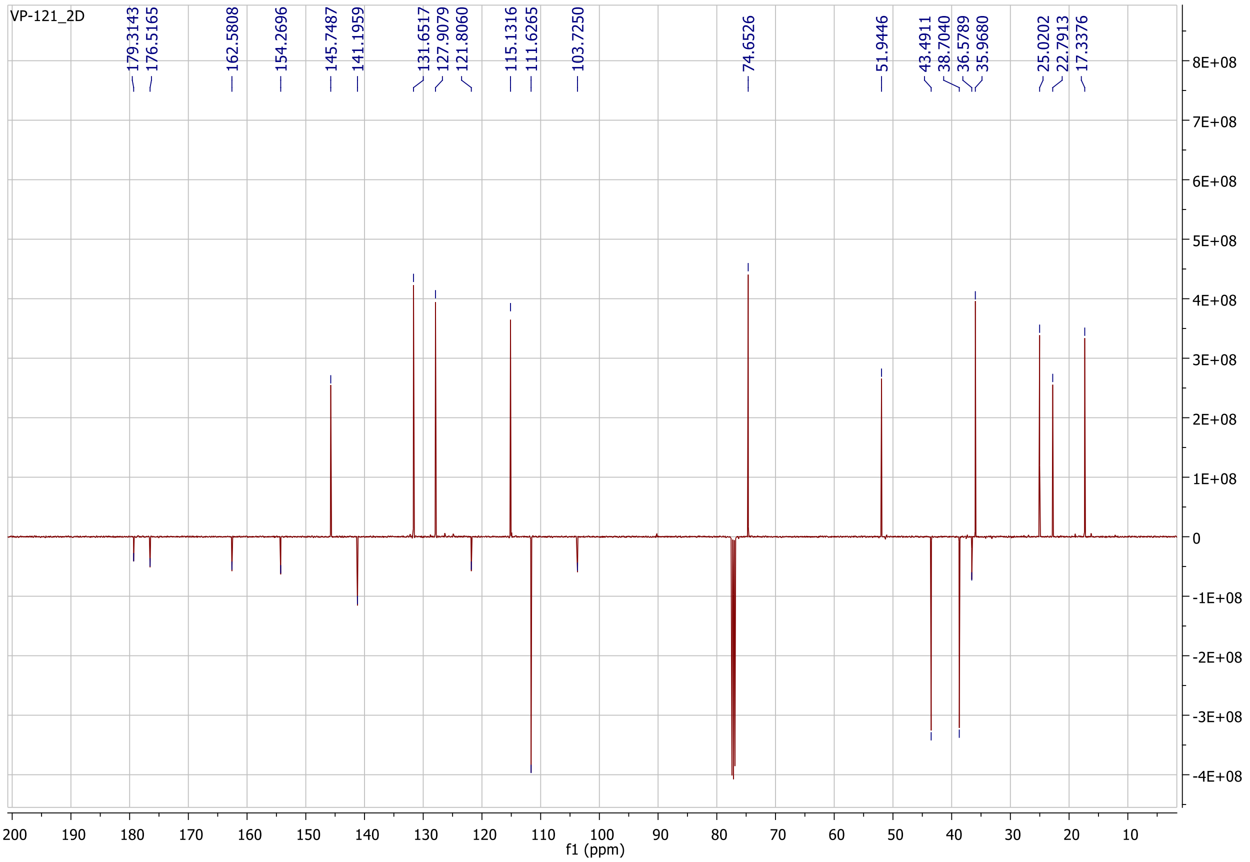

Figure S16. ^{13}C NMR JMOD spectrum of **3** ($CDCl_3$, 125 MHz)

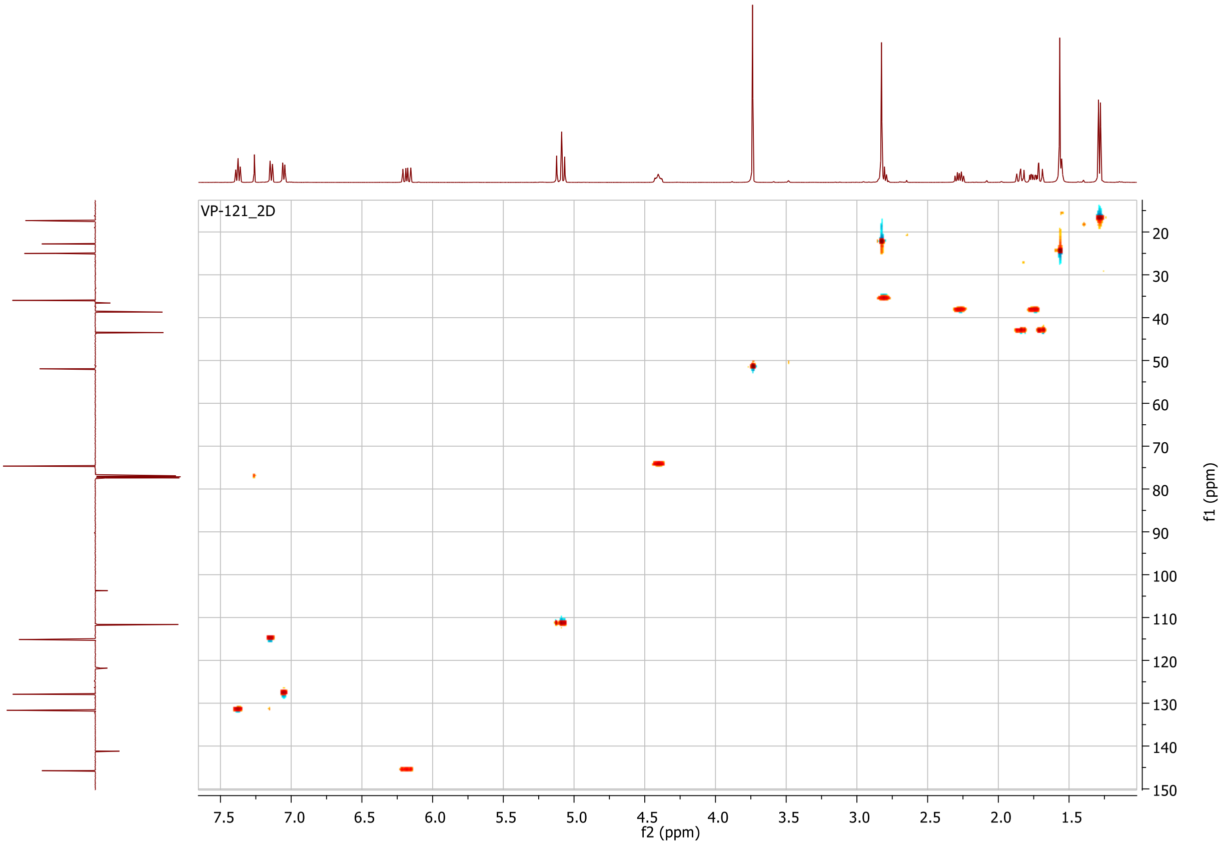

Figure S17. HSQC spectrum of **3** ($CDCl_3$, 125/500 MHz)

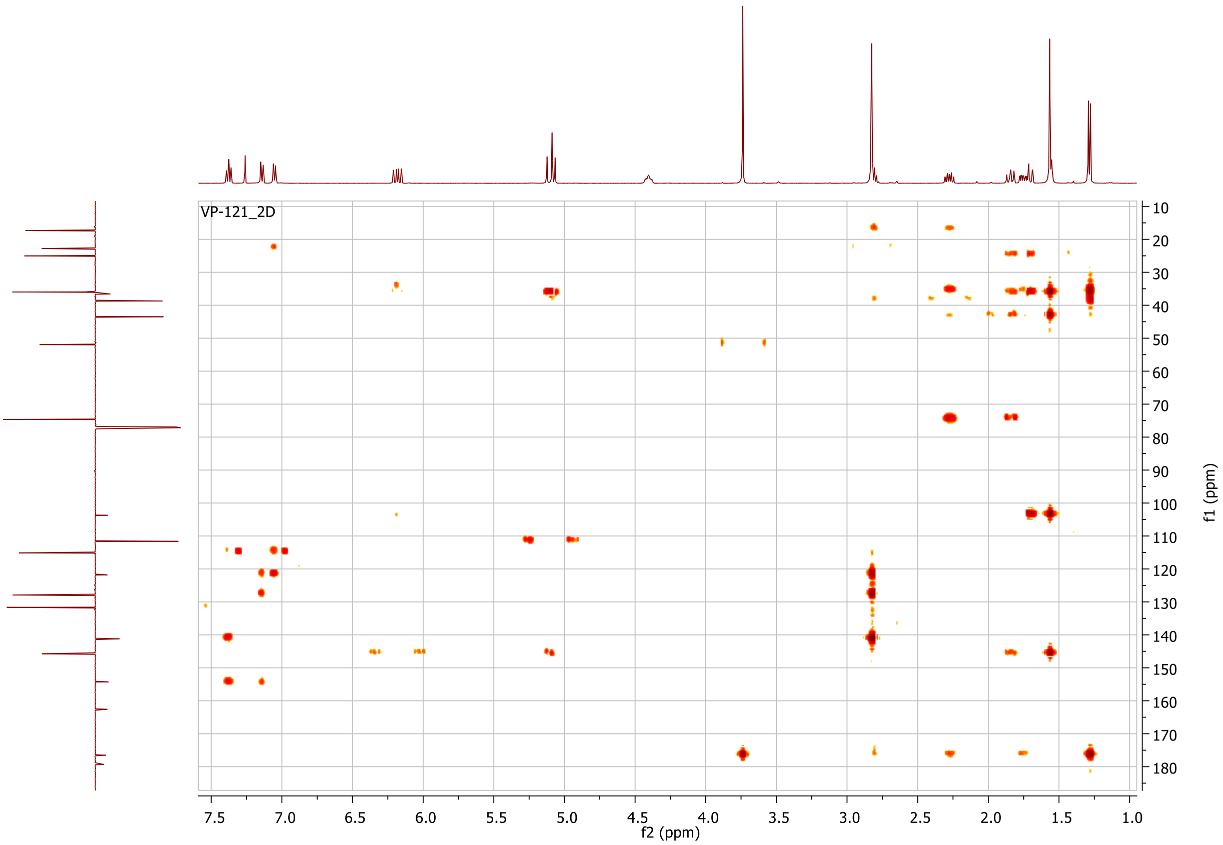

Figure S18. HMBC spectrum of **3** ($CDCl_3$, 125/500 MHz)

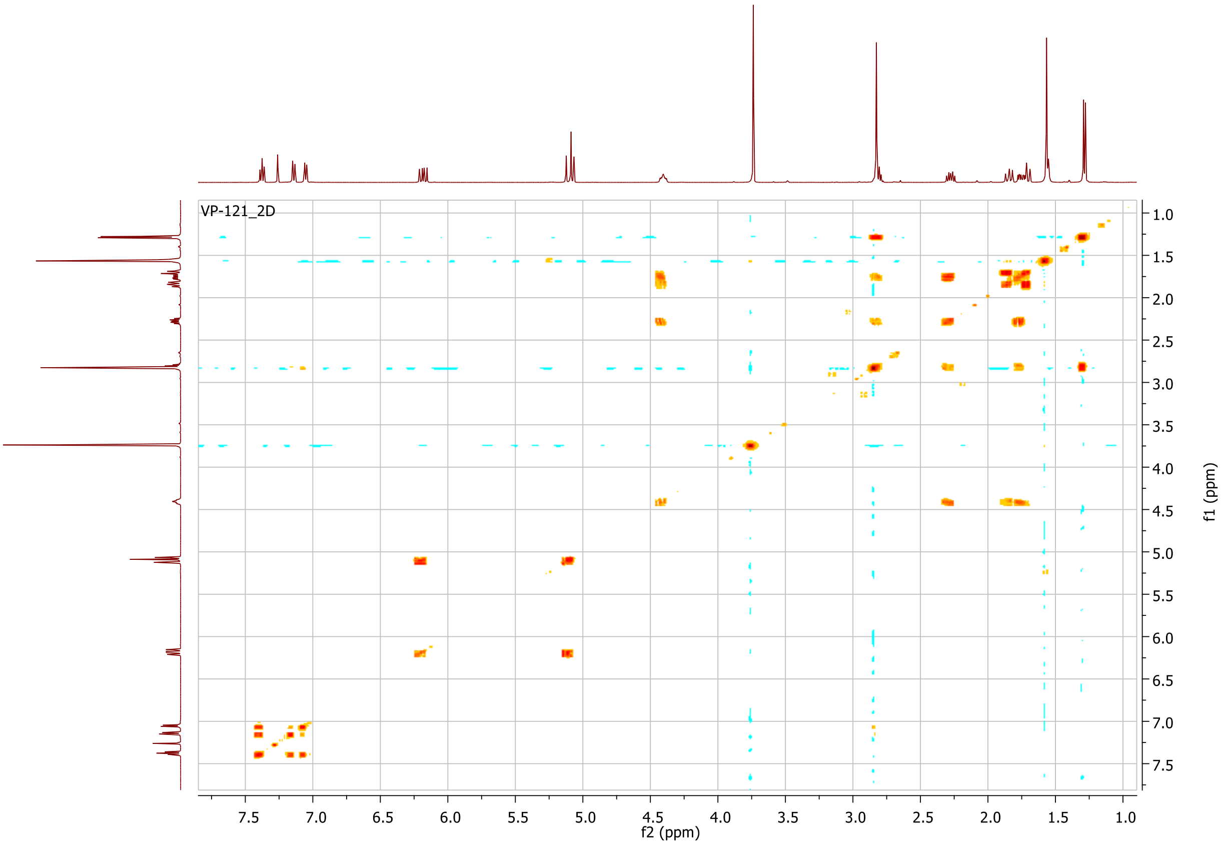

Figure S19. COSY spectrum of **3** ($CDCl_3$, 500 MHz)

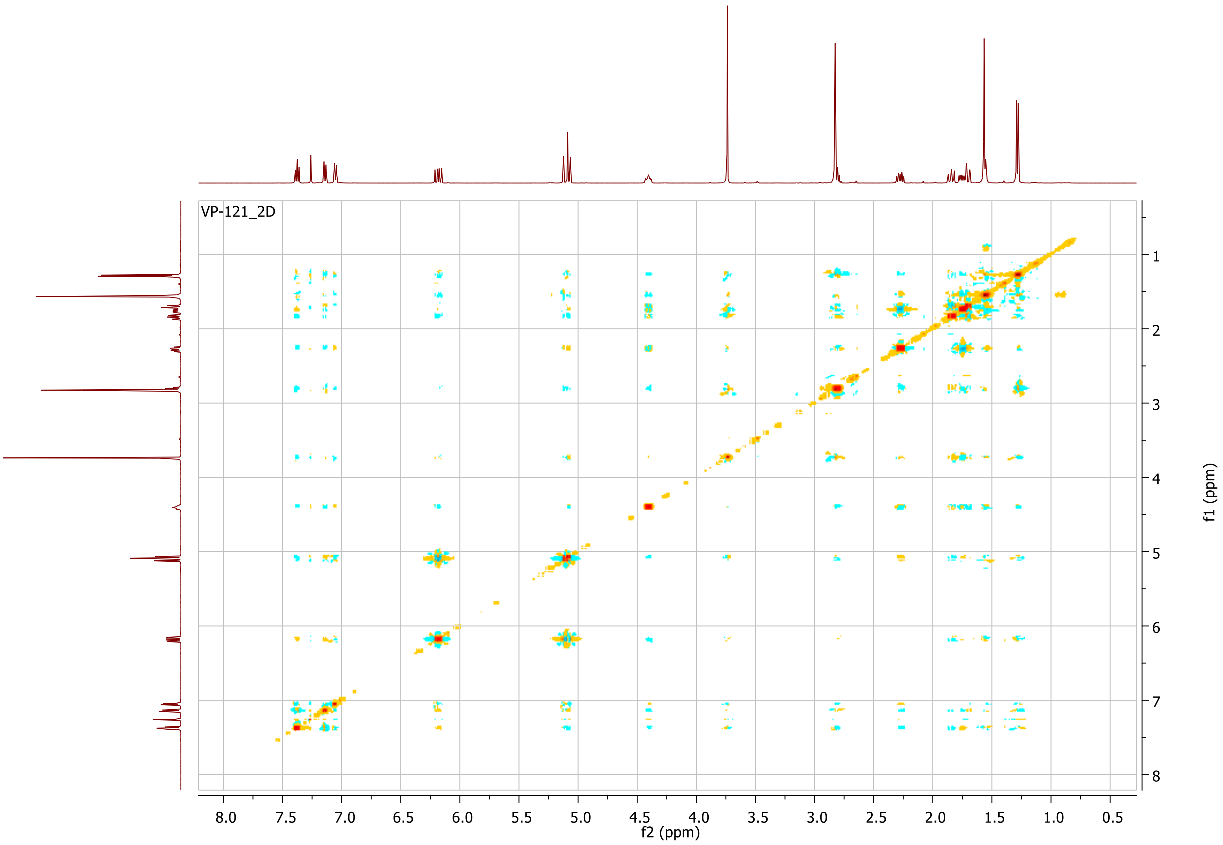

Figure S20. NOESY spectrum of **3** ($CDCl_3$, 500 MHz)

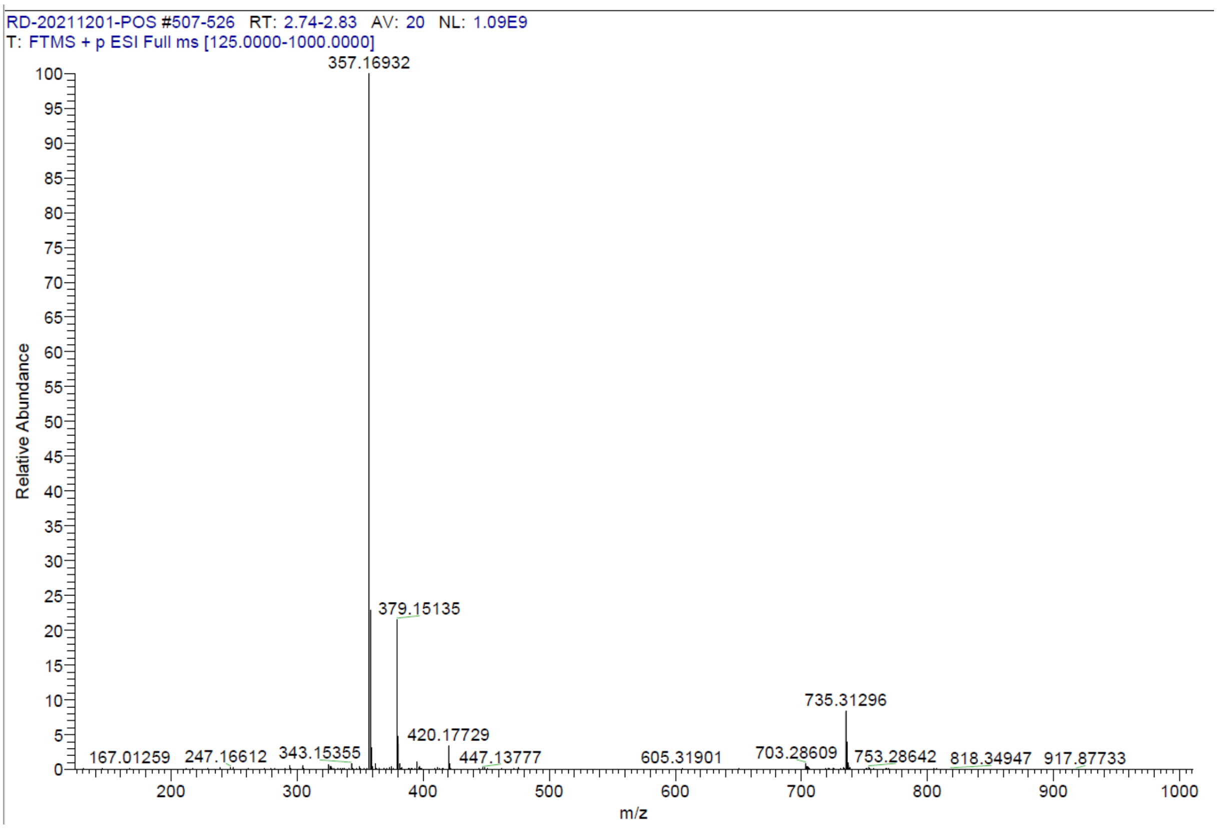

Figure S21. HRESIMS spectrum of **3**

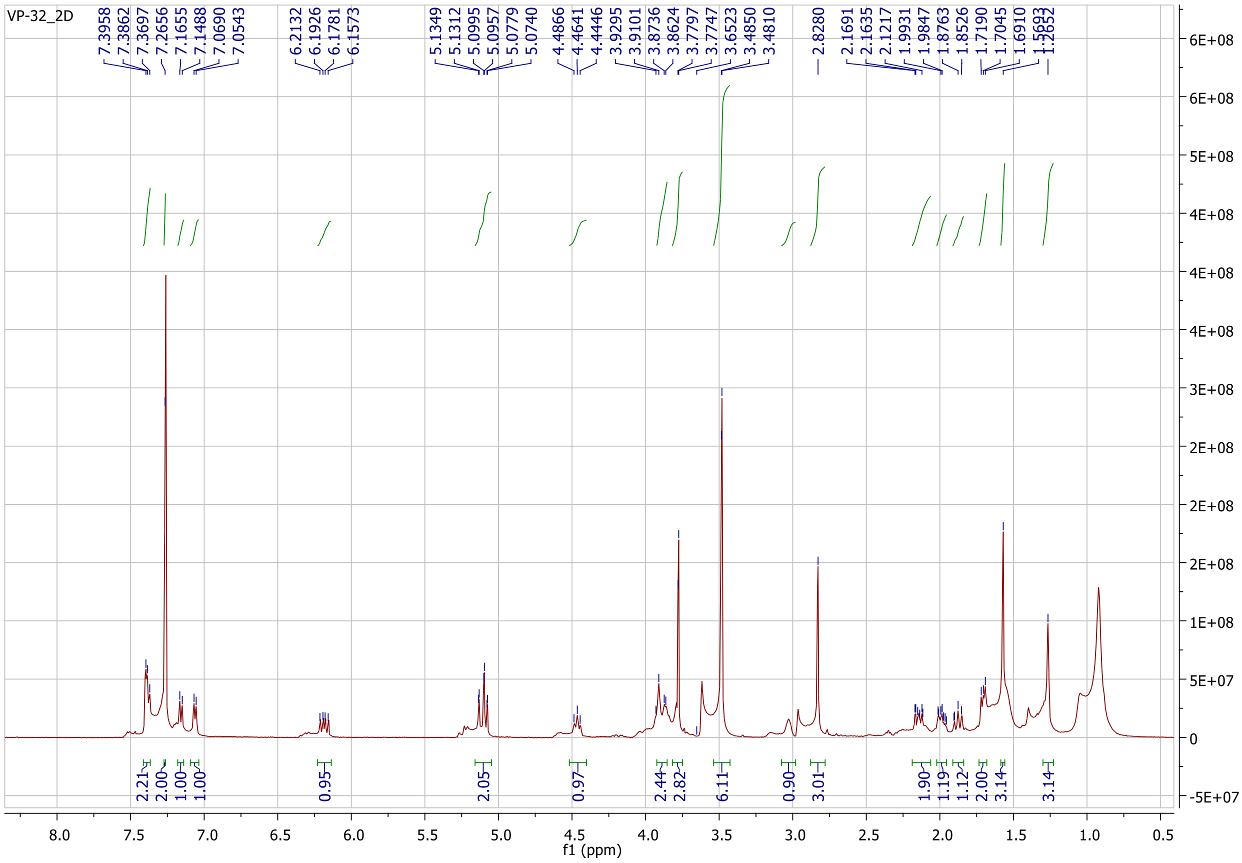

Figure S22. ^{1}H NMR spectrum of **4** ($CDCl_3$, 500 MHz)

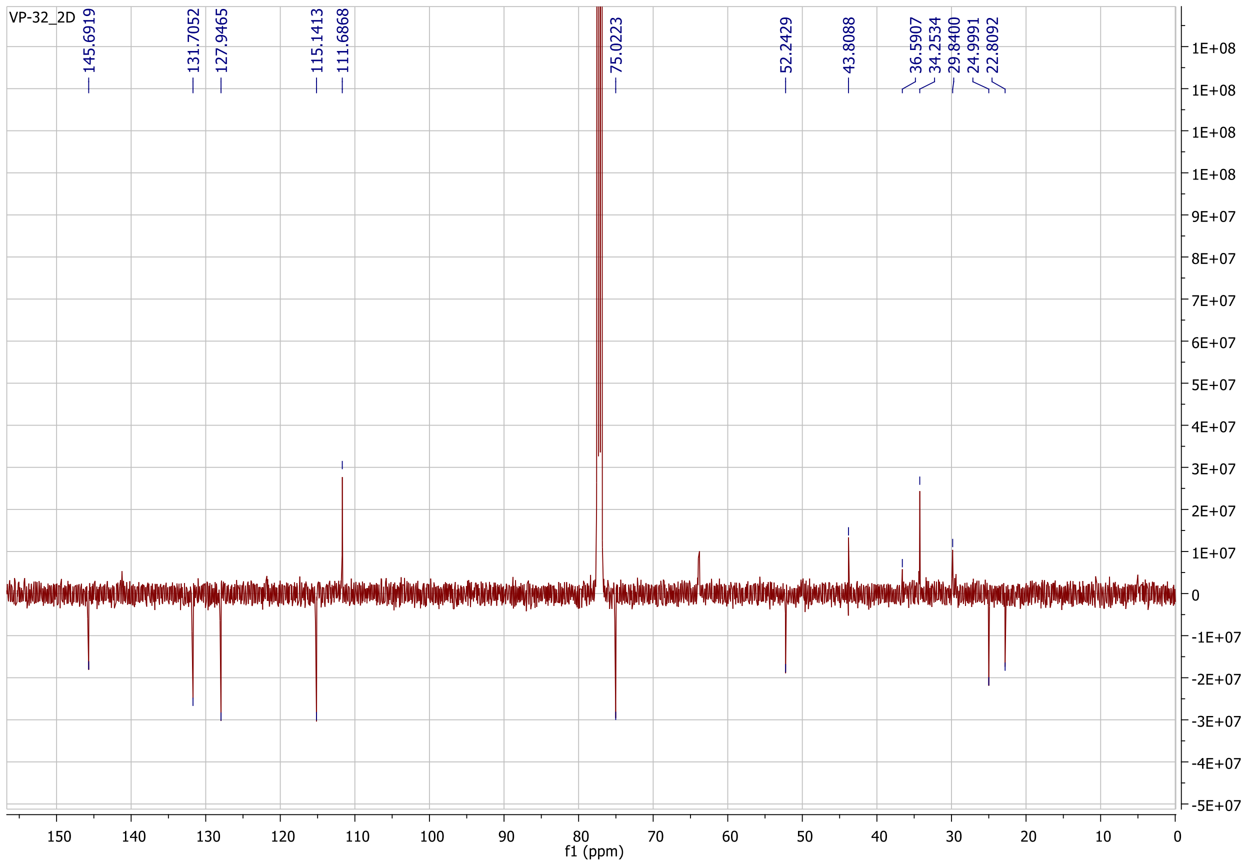

Figure S23. ^{13}C NMR JMOD spectrum of **4** ($CDCl_3$, 125 MHz)

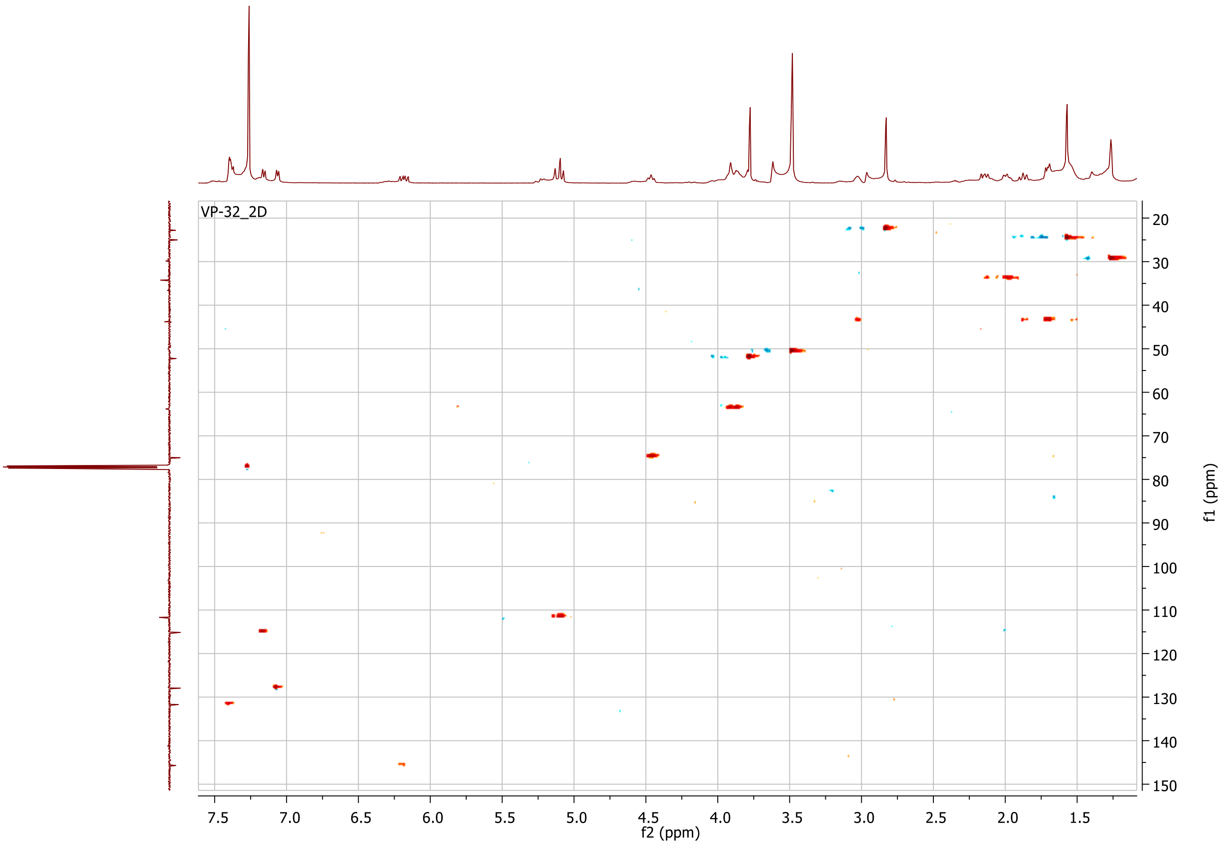

Figure S24. HSQC spectrum of **4** ($CDCl_3$, 125/500 MHz)

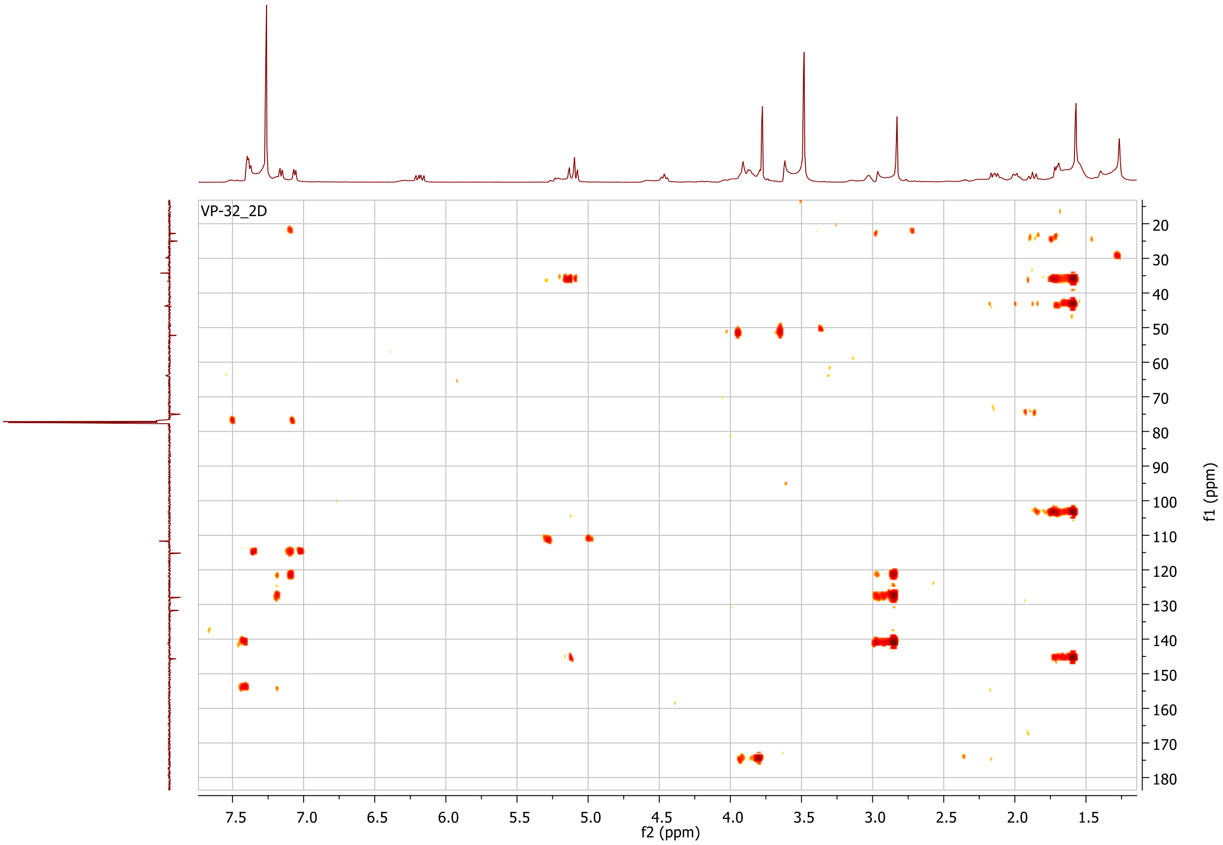

Figure S25. HMBC spectrum of **4** ($CDCl_3$, 125/500 MHz)

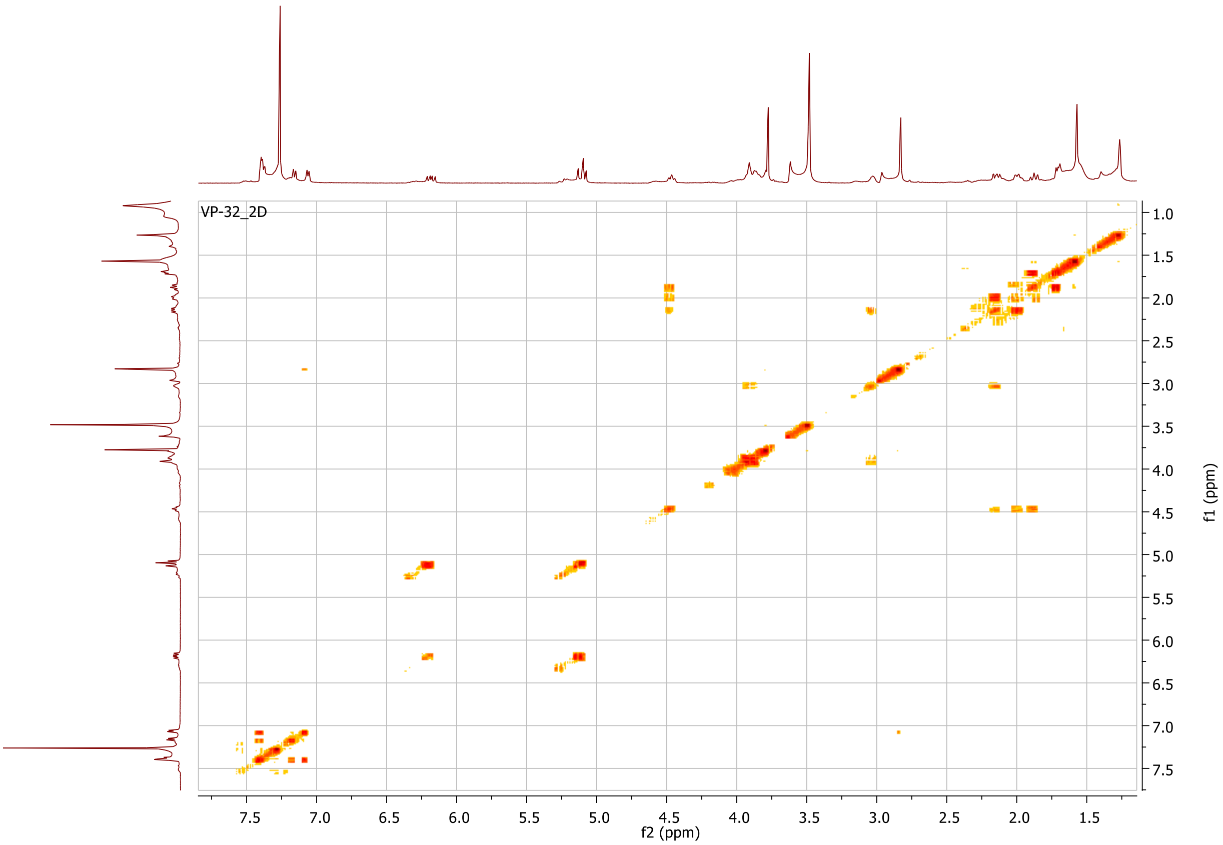

Figure S26. COSY spectrum of **4** ($CDCl_3$, 500 MHz)

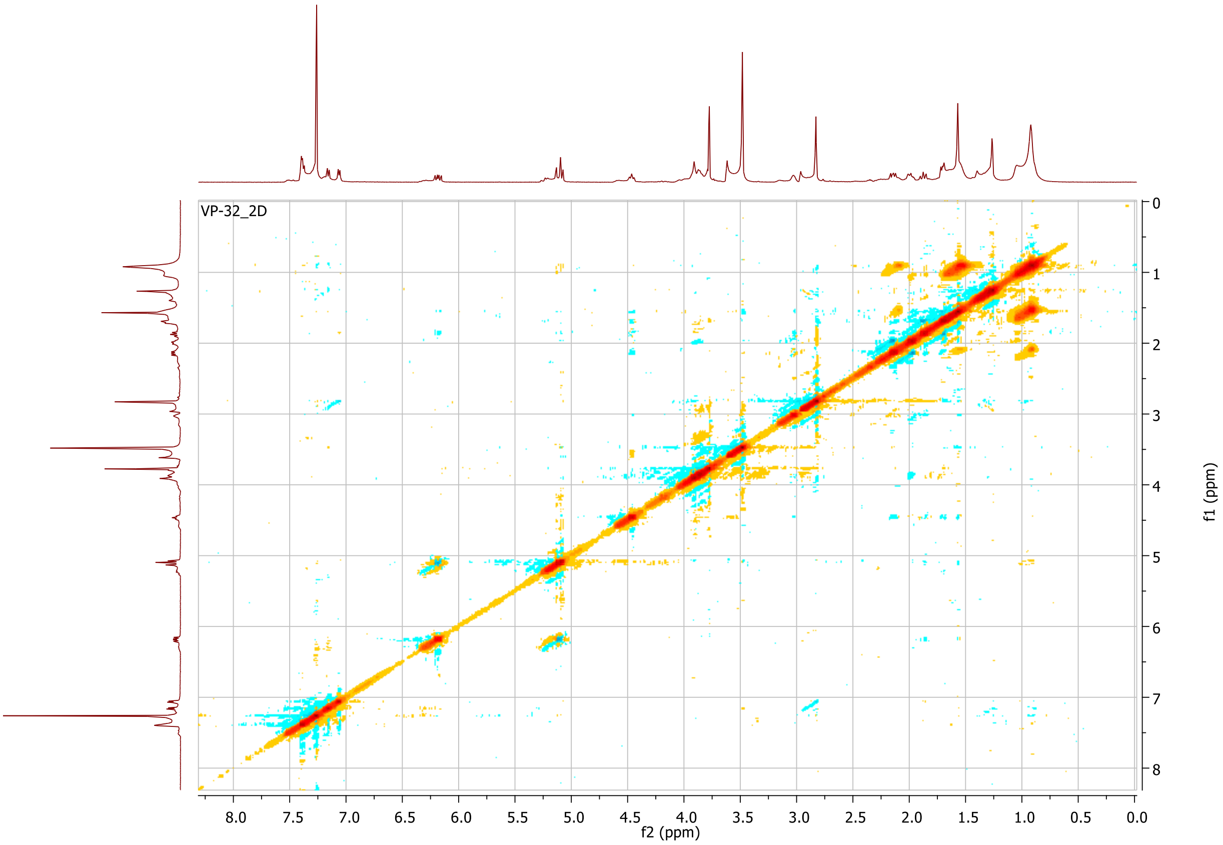

Figure S27. NOESY spectrum of **4** ($CDCl_3$, 500 MHz)

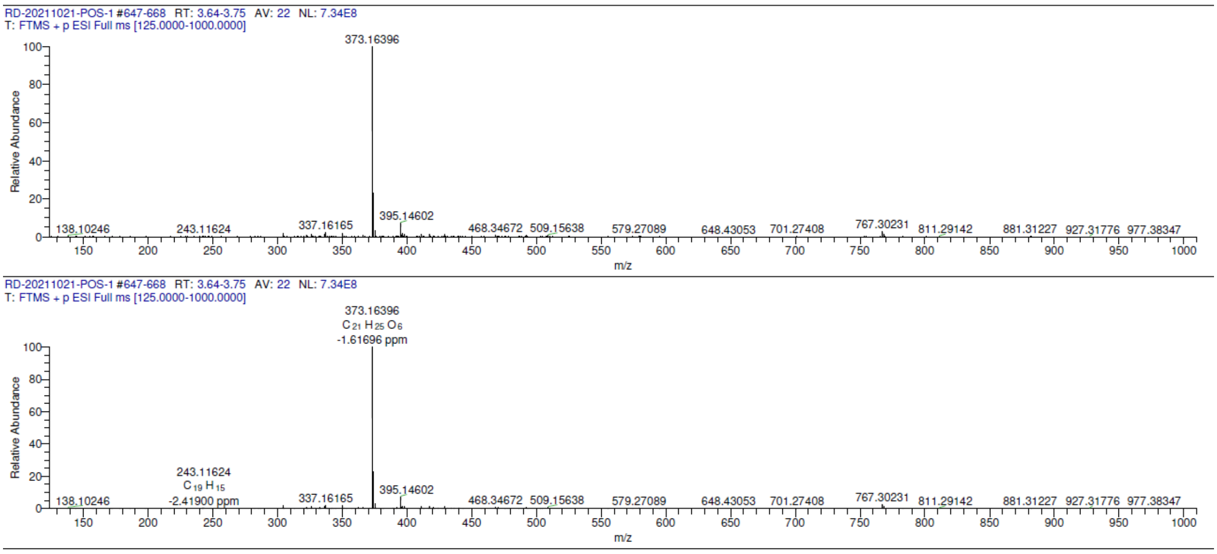

Figure S28. HRESIMS spectrum of **4**

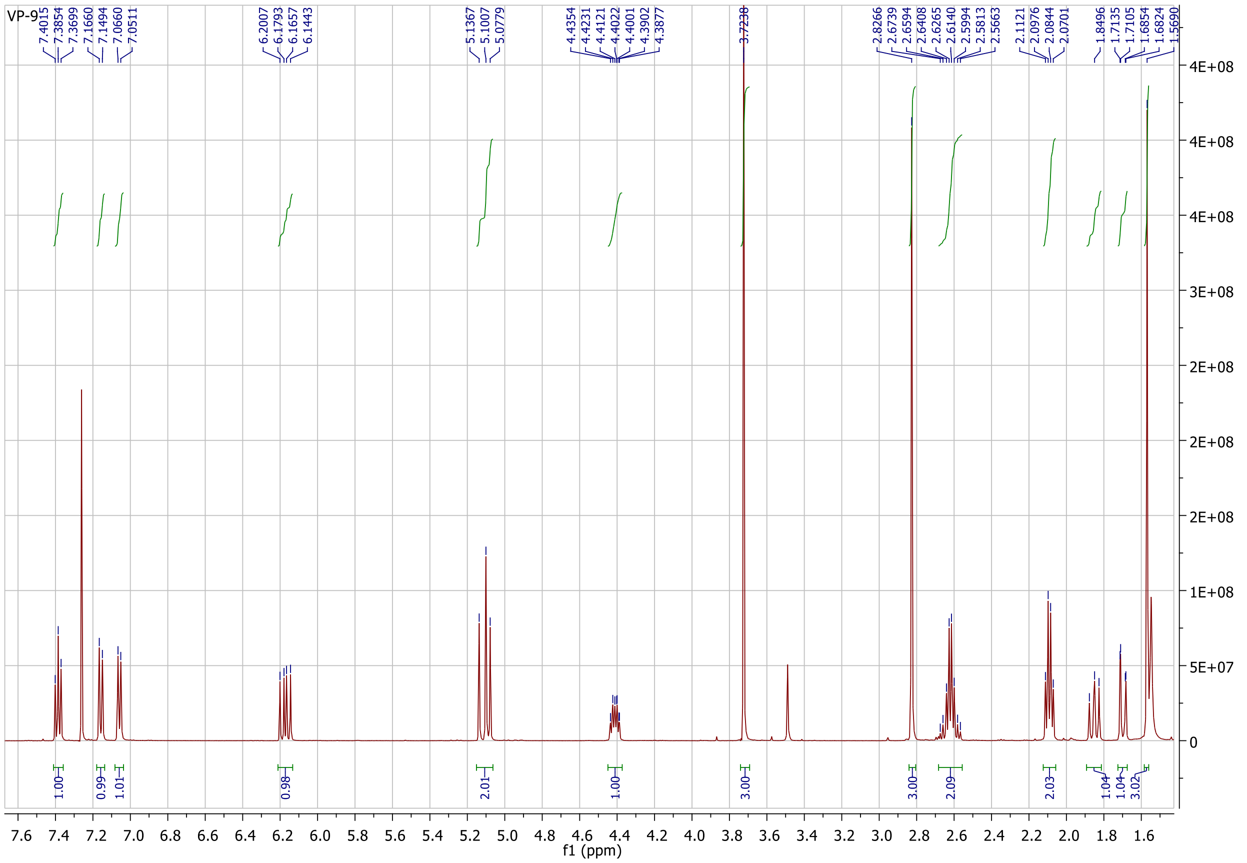

Figure S29. ^{1}H NMR spectrum of **5** ($CDCl_3$, 500 MHz)

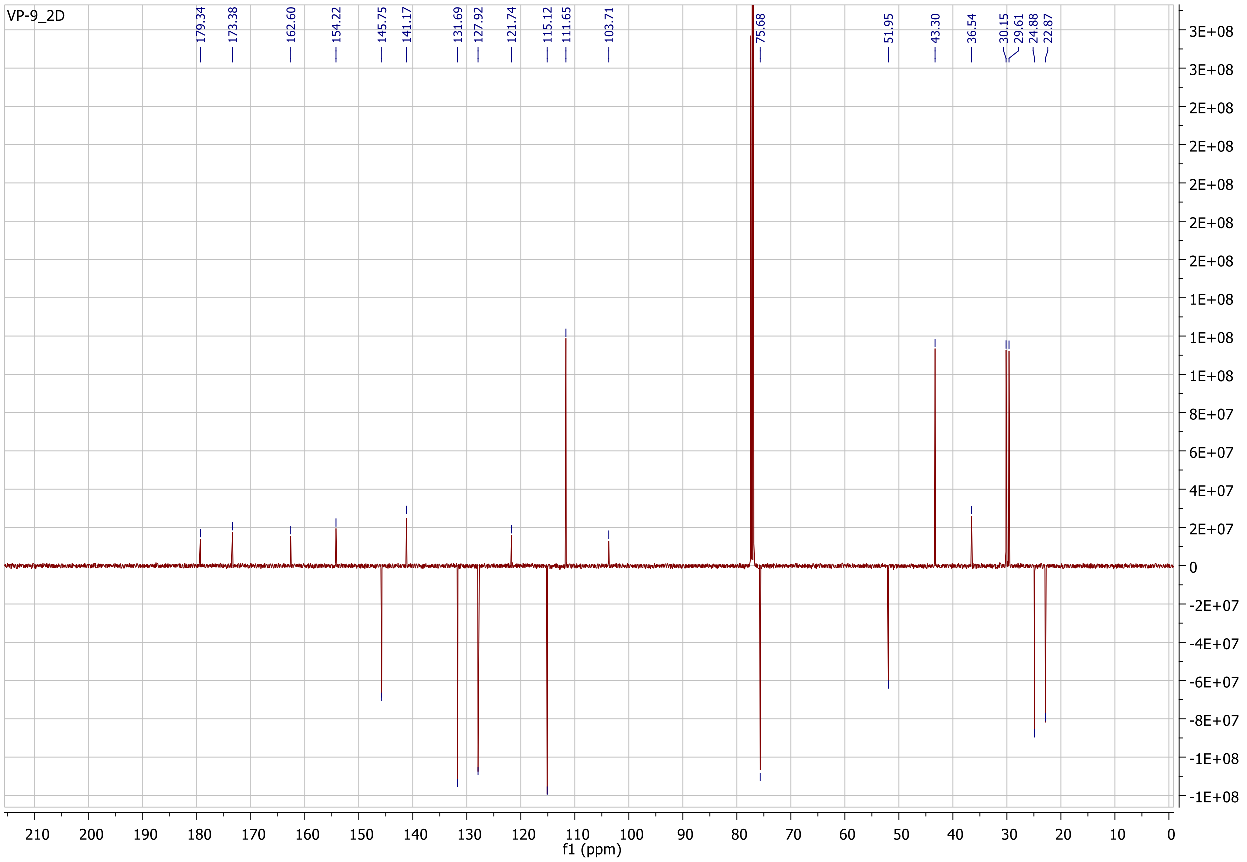

Figure S30. ^{13}C NMR JMOD spectrum of **5** ($CDCl_3$, 500 MHz)

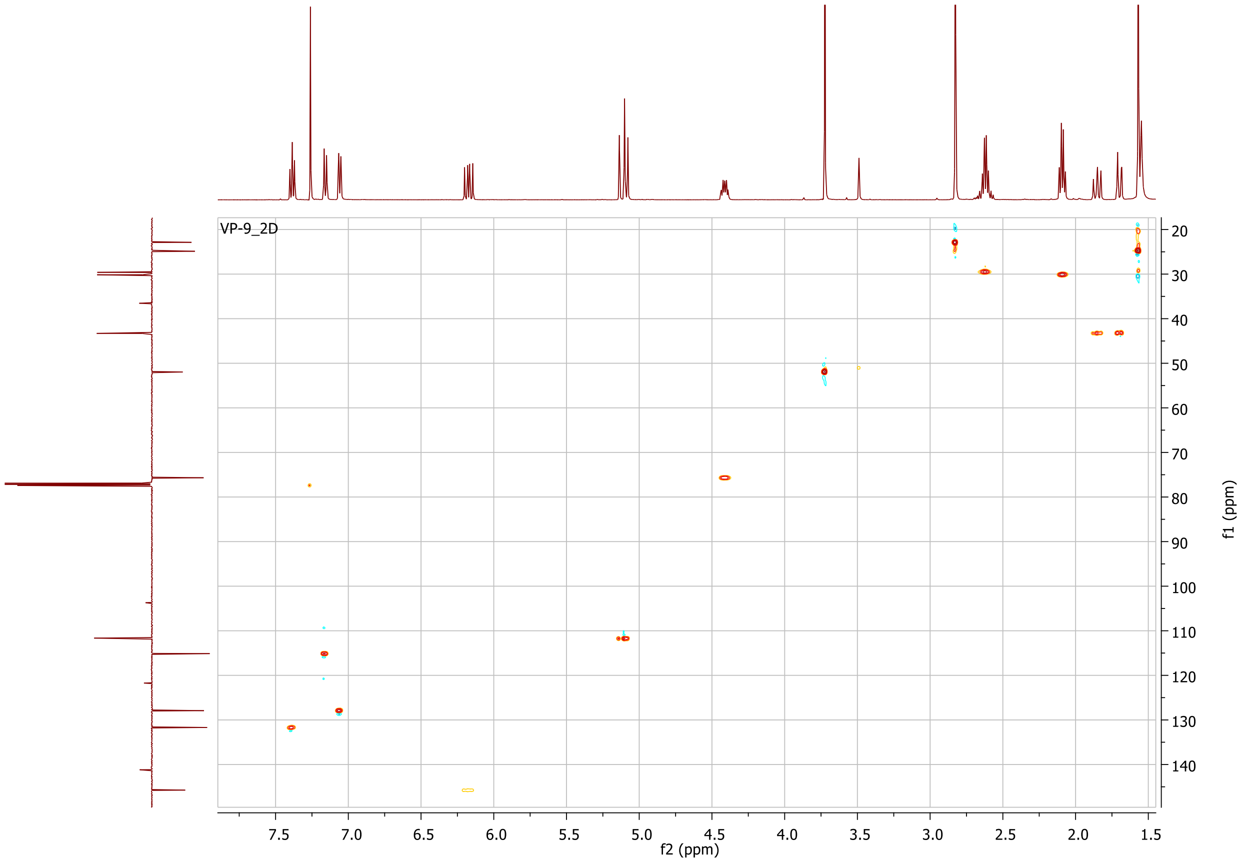

Figure S31. HSQC spectrum of **5** ($CDCl_3$, 125/500 MHz)

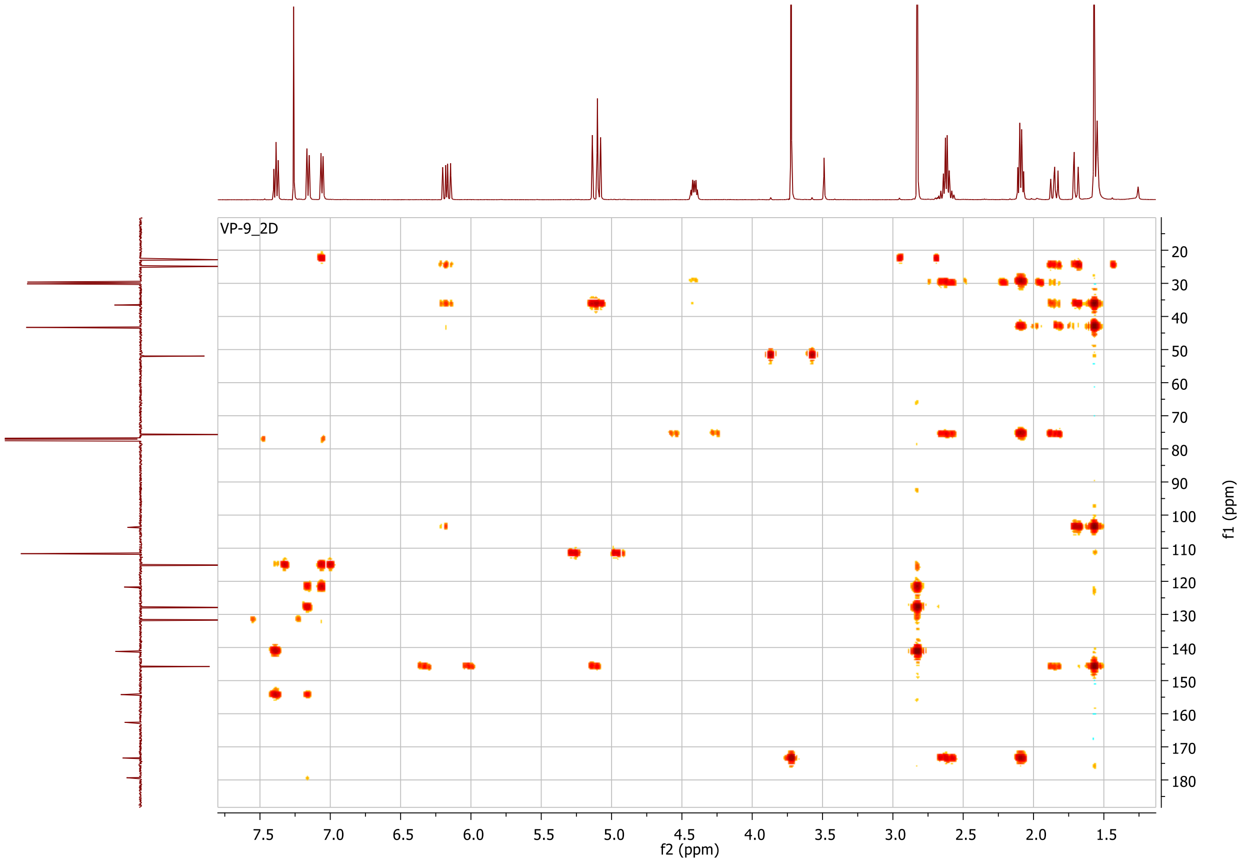

Figure S32. HMBC spectrum of **5** ($CDCl_3$, 125/500 MHz)

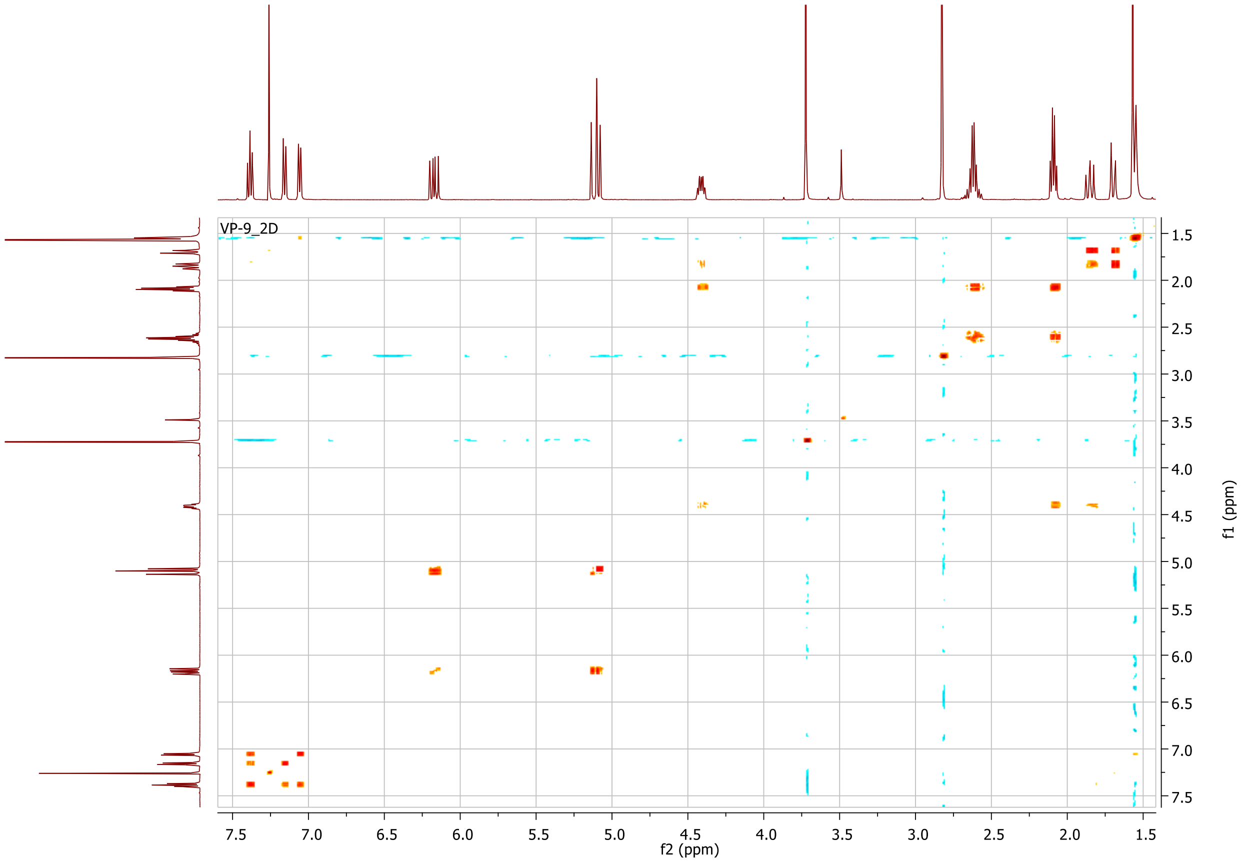

Figure S33. COSY spectrum of **5** ($CDCl_3$, 500 MHz)

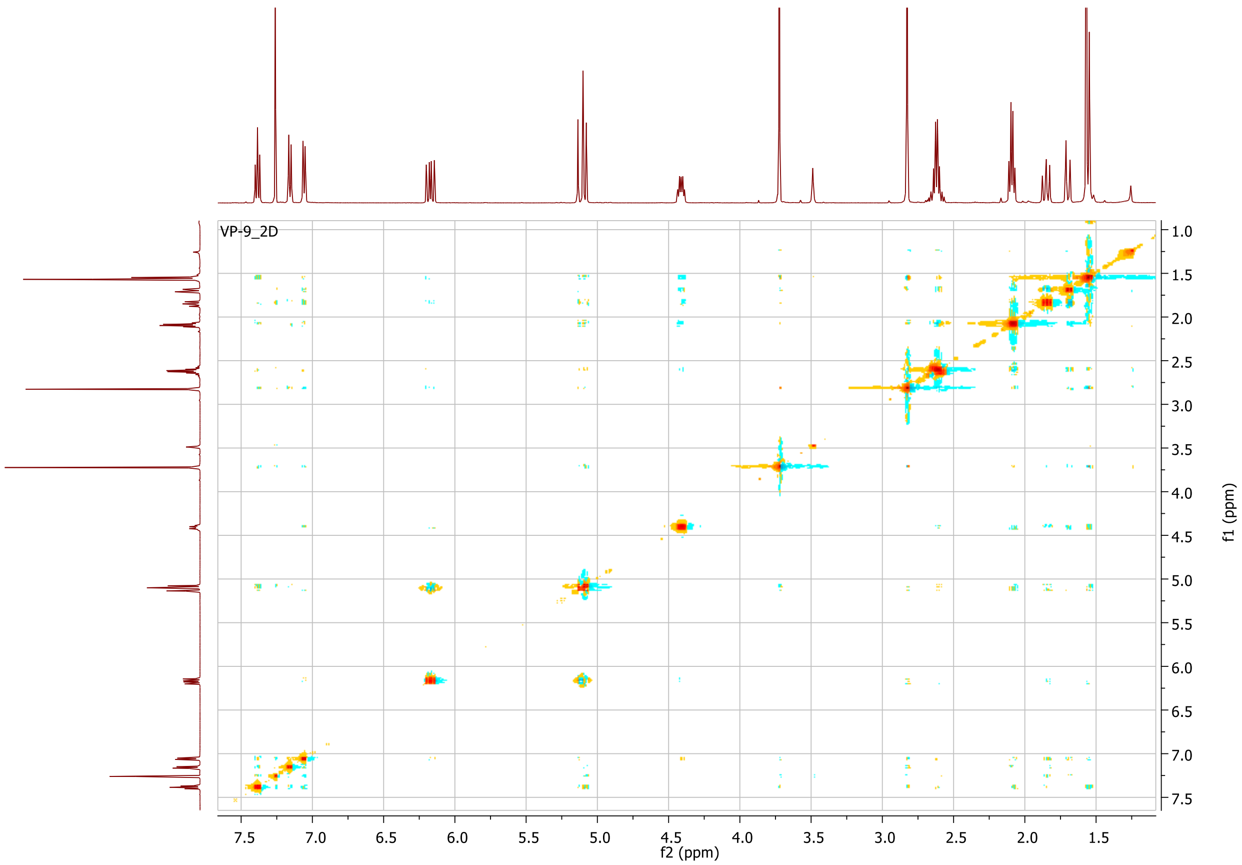

Figure S34. NOESY spectrum of **5** ($CDCl_3$, 500 MHz)

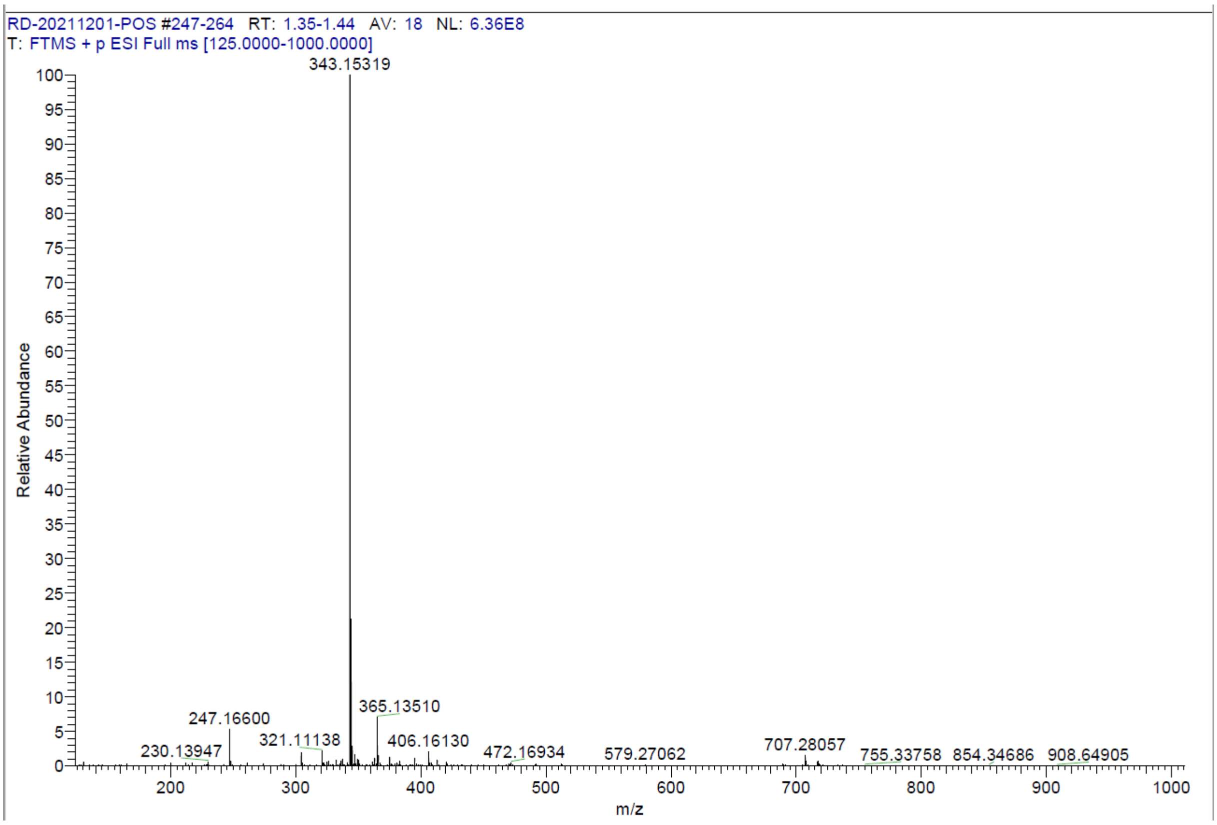

Figure S35. HRESIMS spectrum of **5**

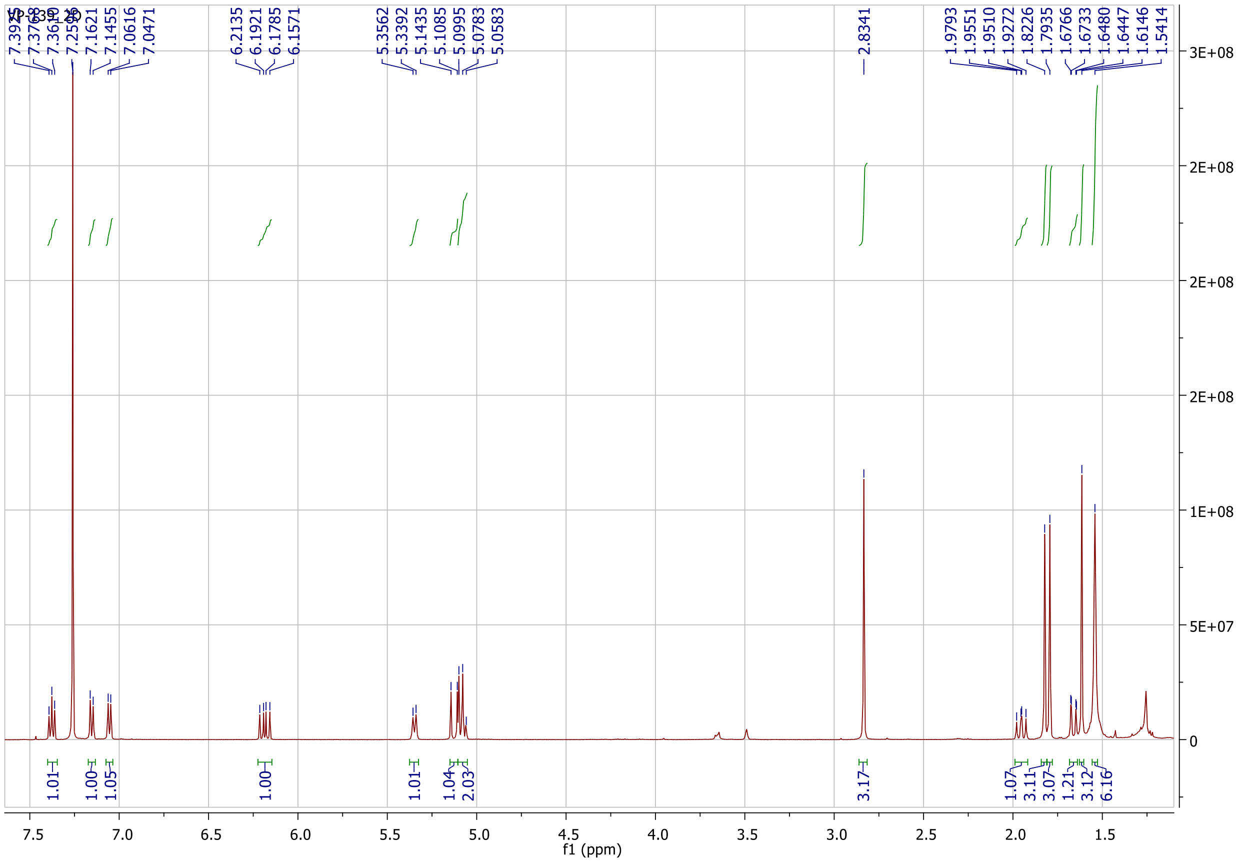

Figure S36. ^{1}H NMR spectrum of **6** ($CDCl_3$, 500 MHz)

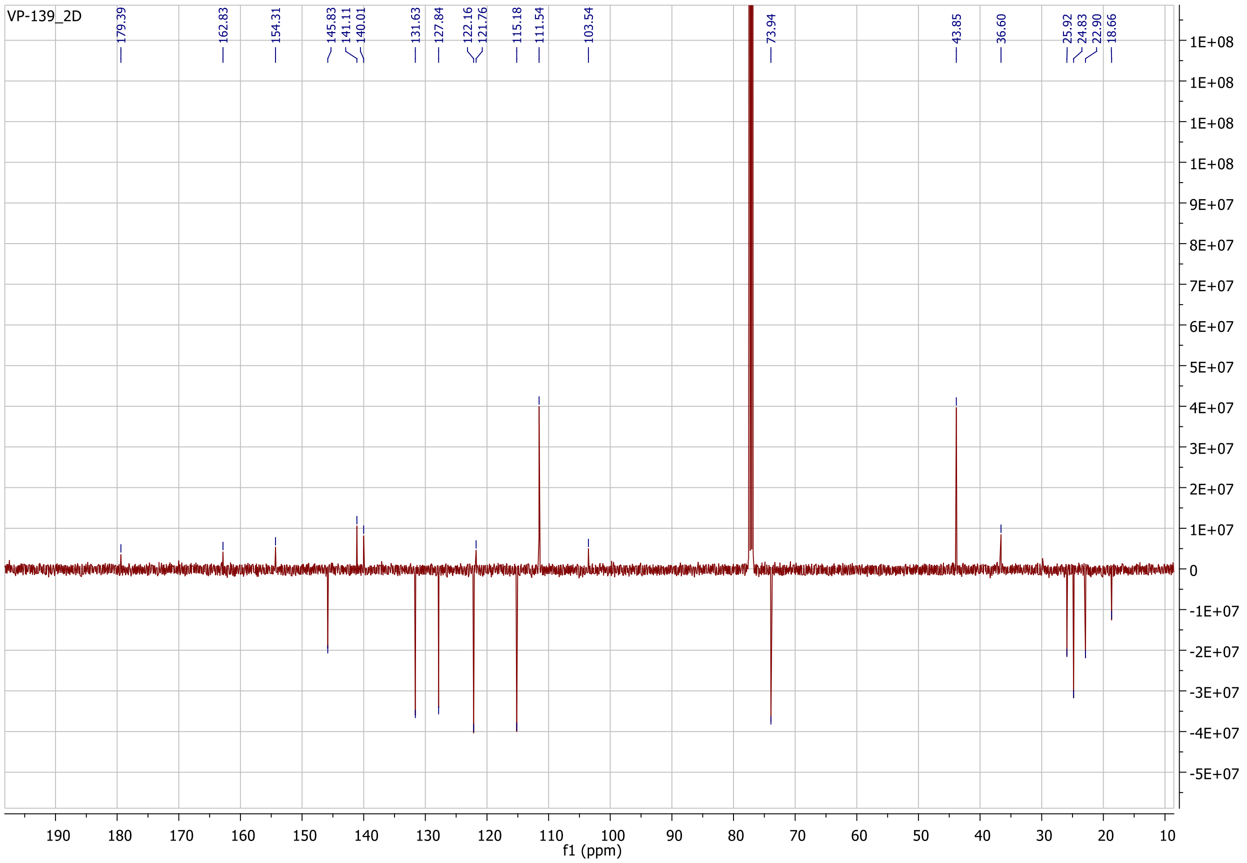

Figure S37. ^{13}C NMR JMOD spectrum of **6** ($CDCl_3$, 500 MHz)

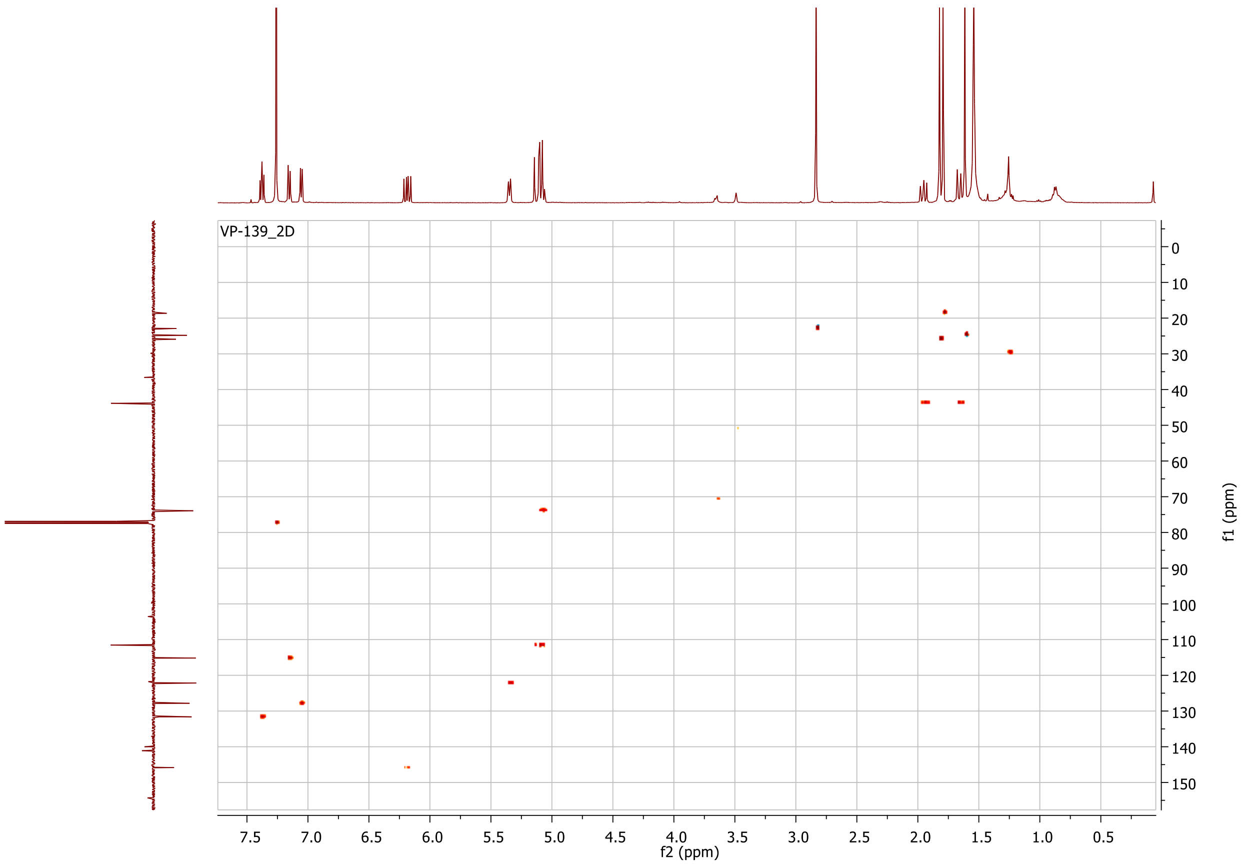

Figure S38. HSQC spectrum of **6** ($CDCl_3$, 125/500 MHz)

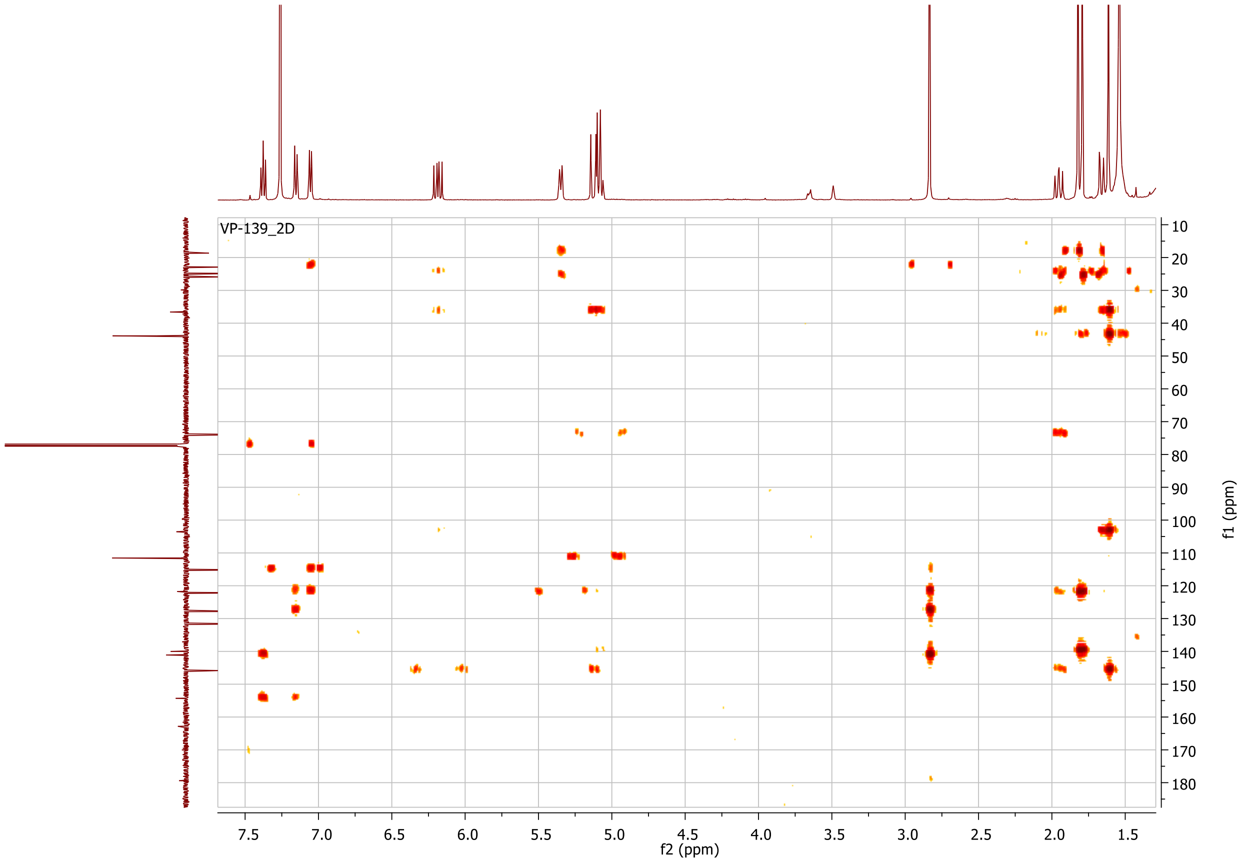

Figure S39. HMBC spectrum of **6** ($CDCl_3$, 125/500 MHz)

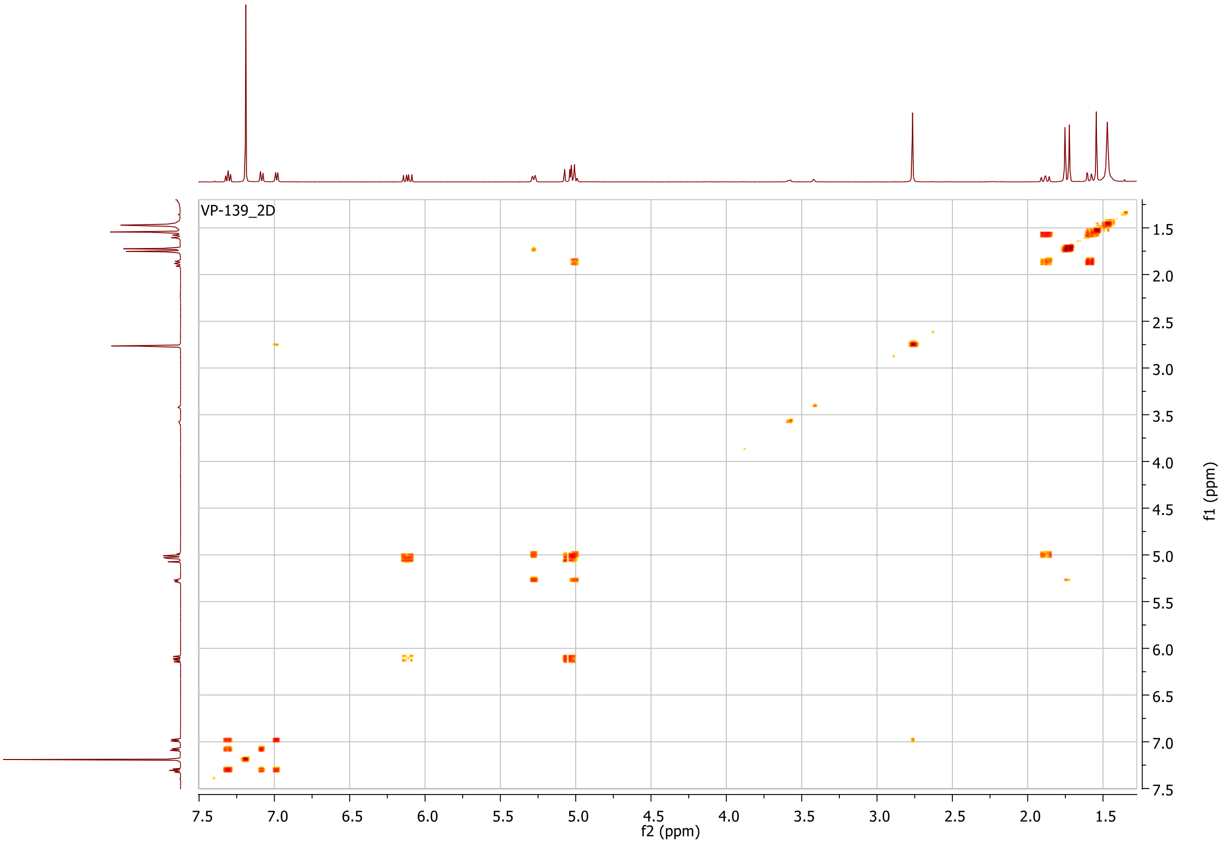

Figure S40. COSY spectrum of **6** ($CDCl_3$, 500 MHz)

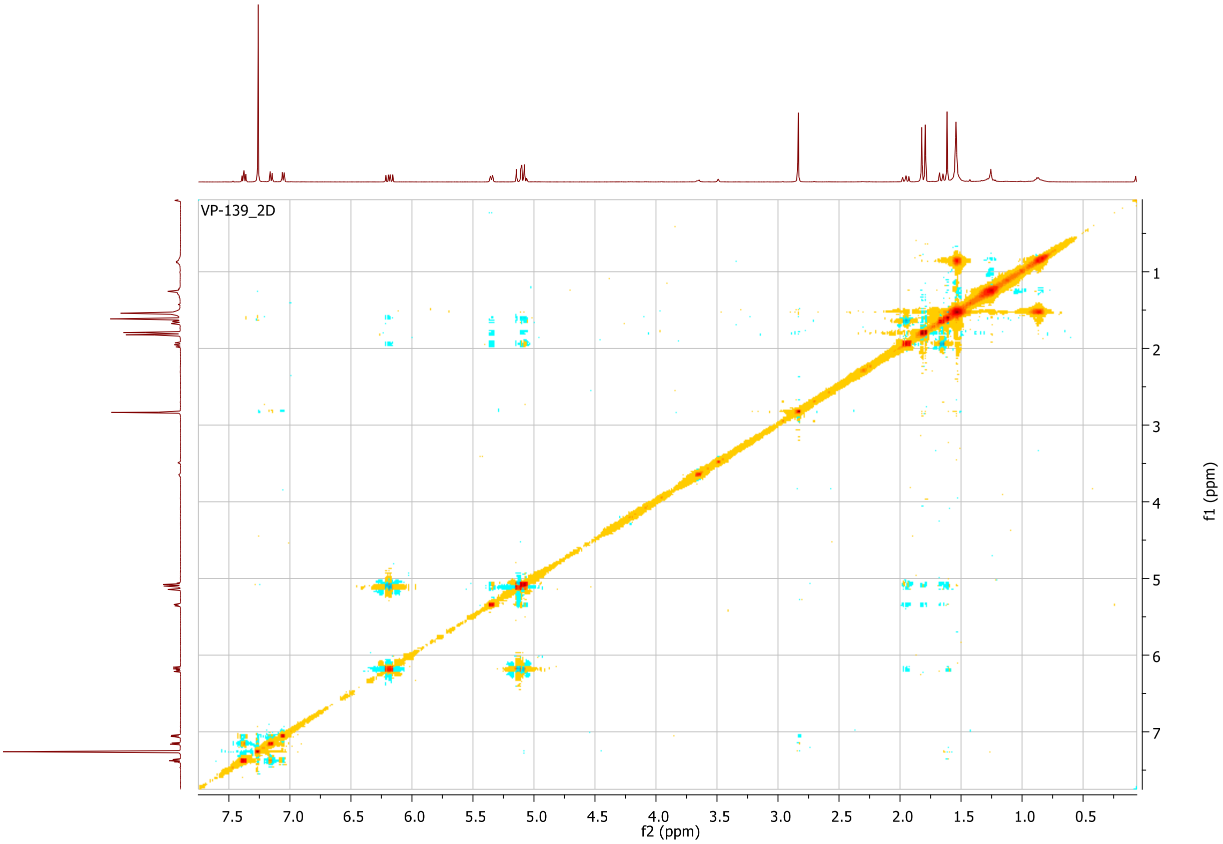

Figure S41. NOESY spectrum of **6** ($CDCl_3$, 500 MHz)

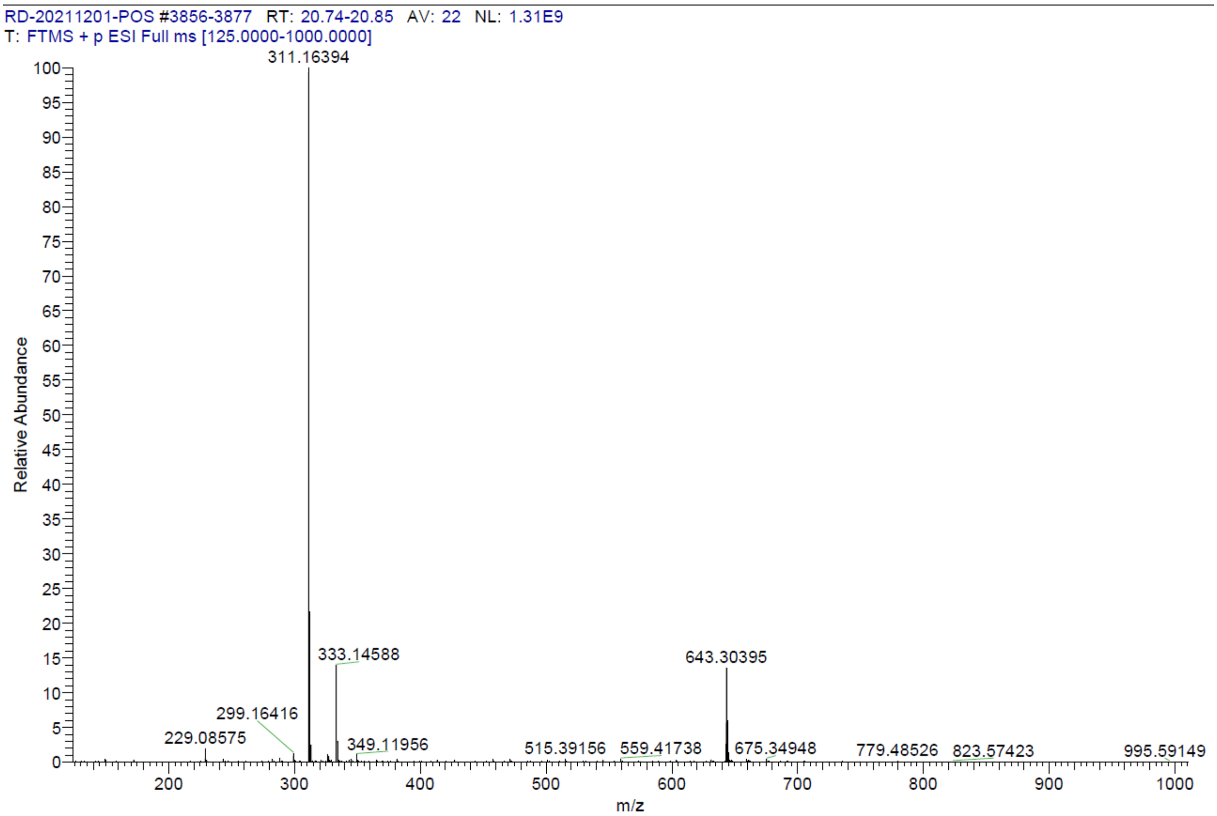

Figure S42. HRESIMS spectrum of **6**

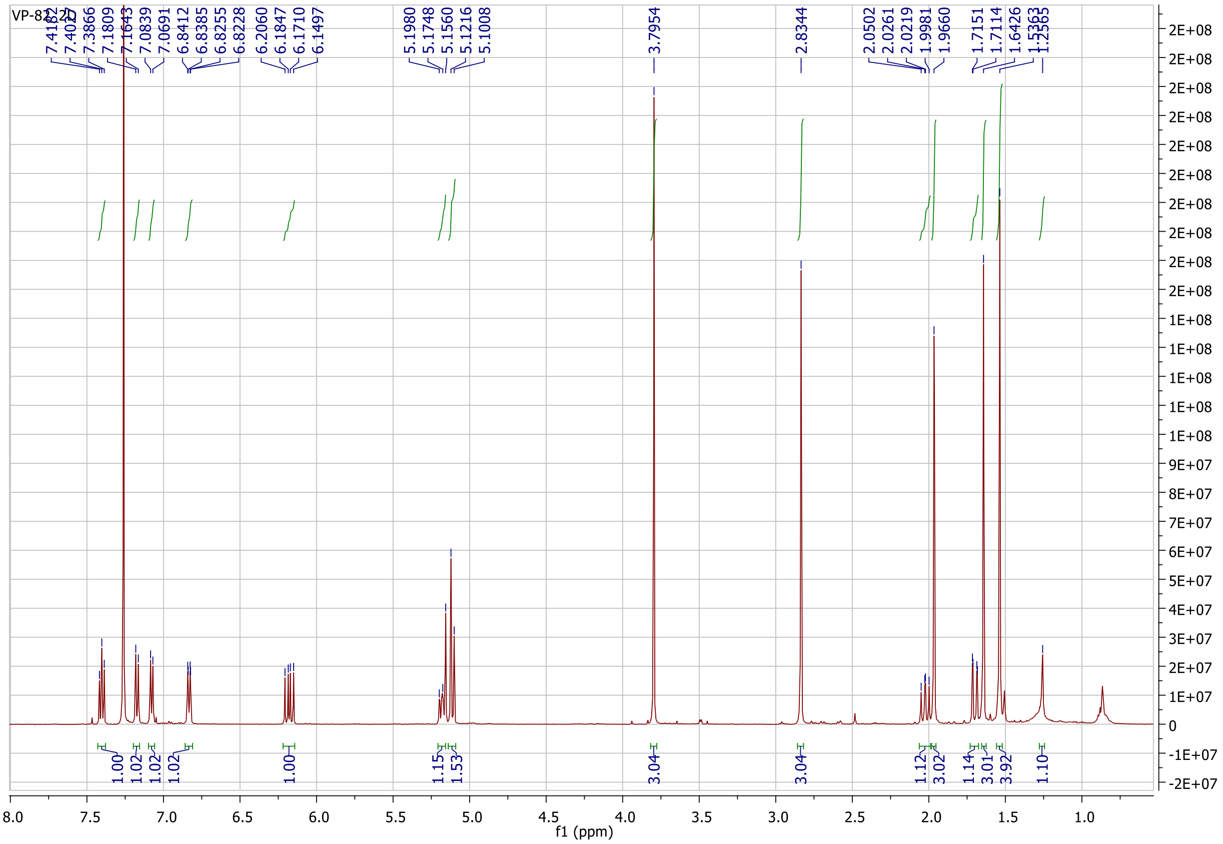

Figure S43. ^{1}H NMR spectrum of **7** ($CDCl_3$, 500 MHz)

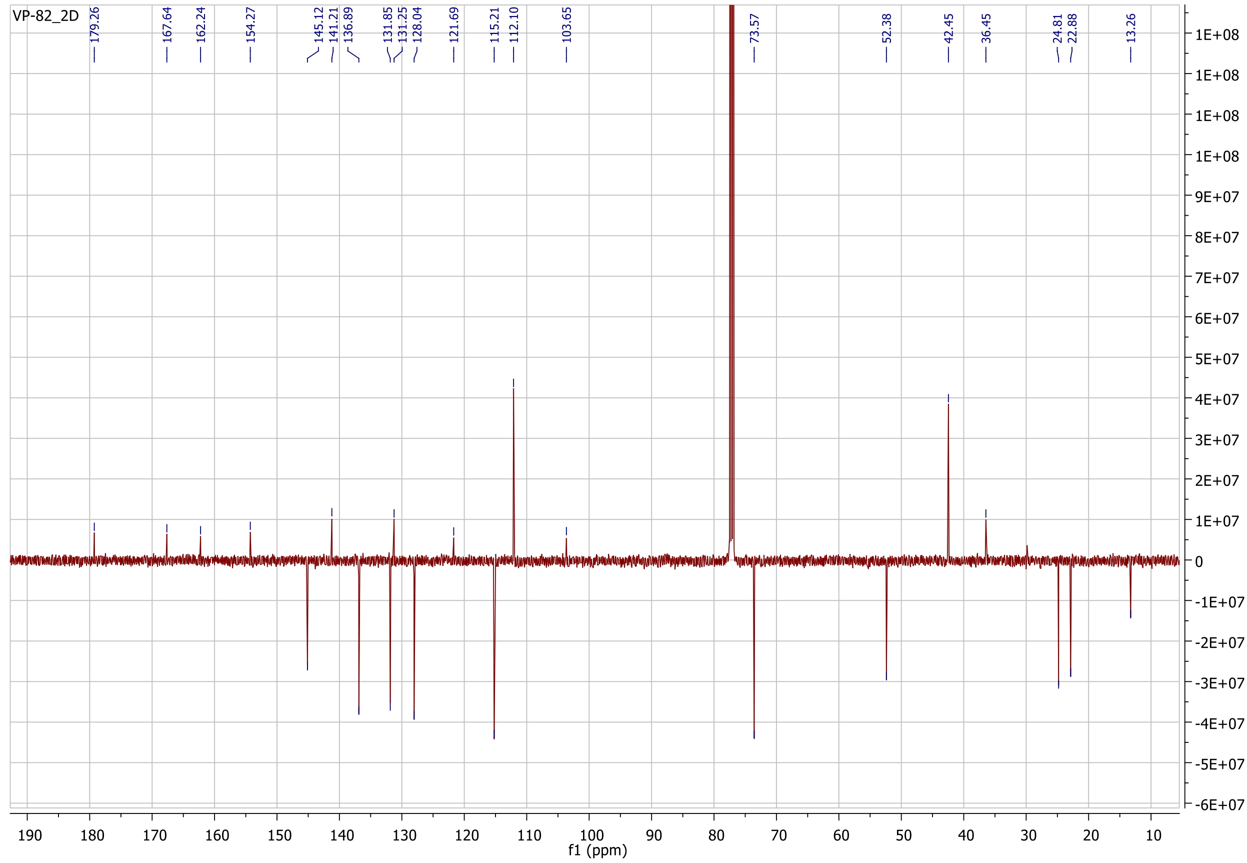

Figure S44. ^{13}C NMR JMOD spectrum of **7** ($CDCl_3$, 500 MHz)

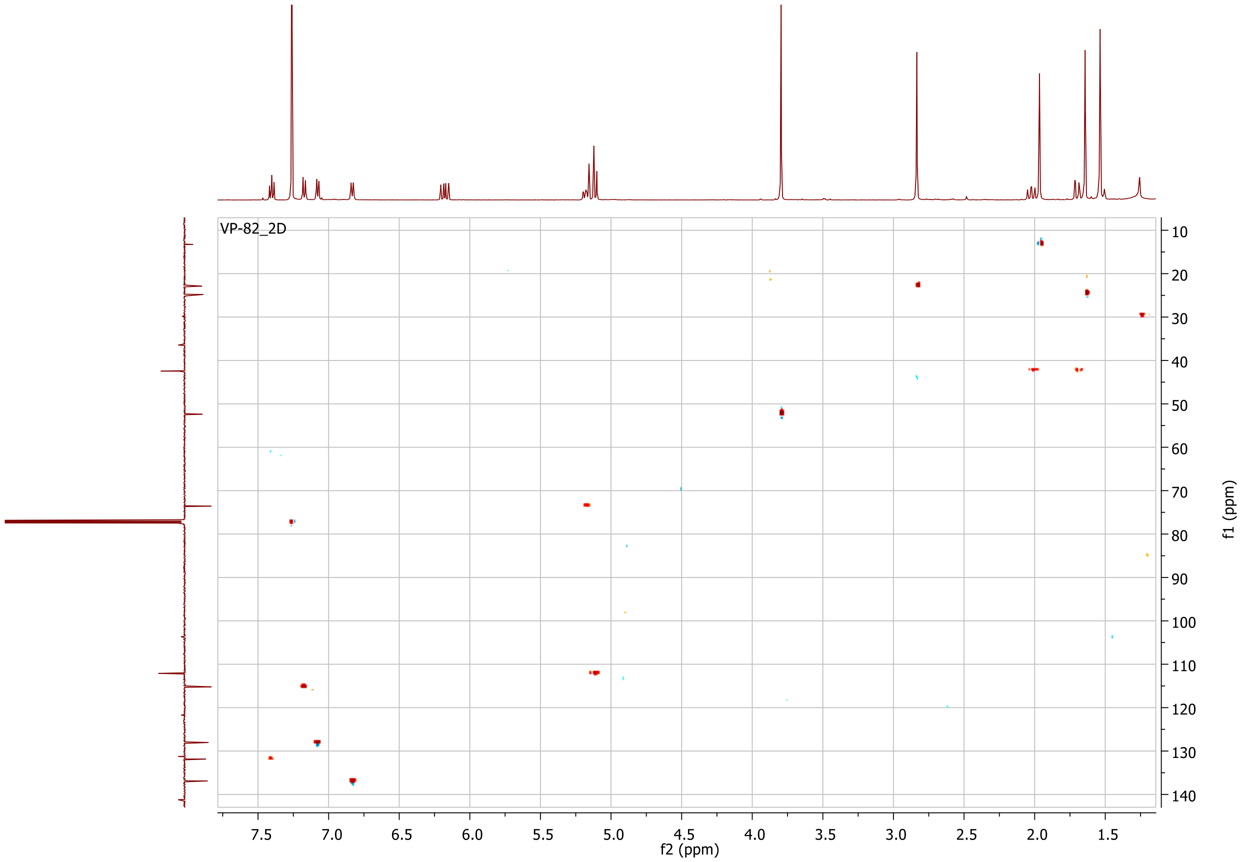

Figure S45. HSQC spectrum of **7** ($CDCl_3$, 125/500 MHz)

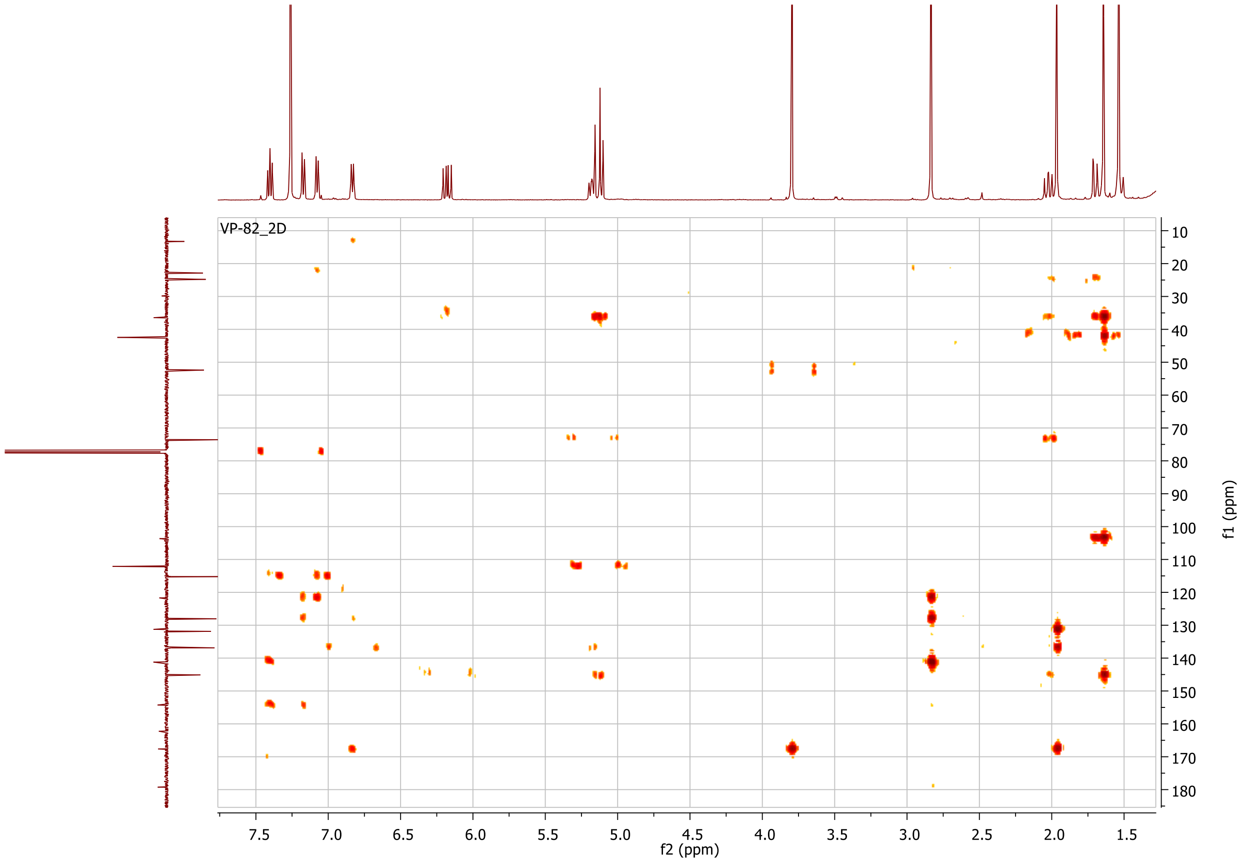

Figure S46. HMBC spectrum of **7** ($CDCl_3$, 125/500 MHz)

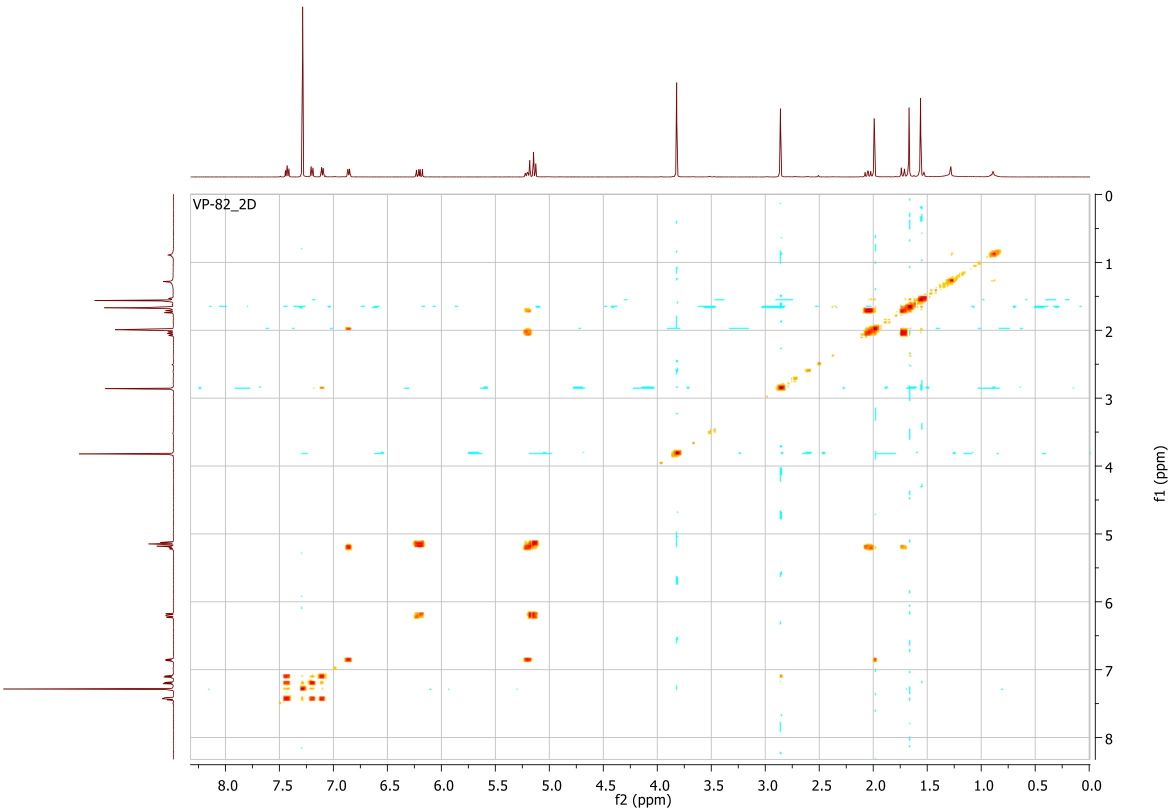

Figure S47. COSY spectrum of **7** ($CDCl_3$, 500 MHz)

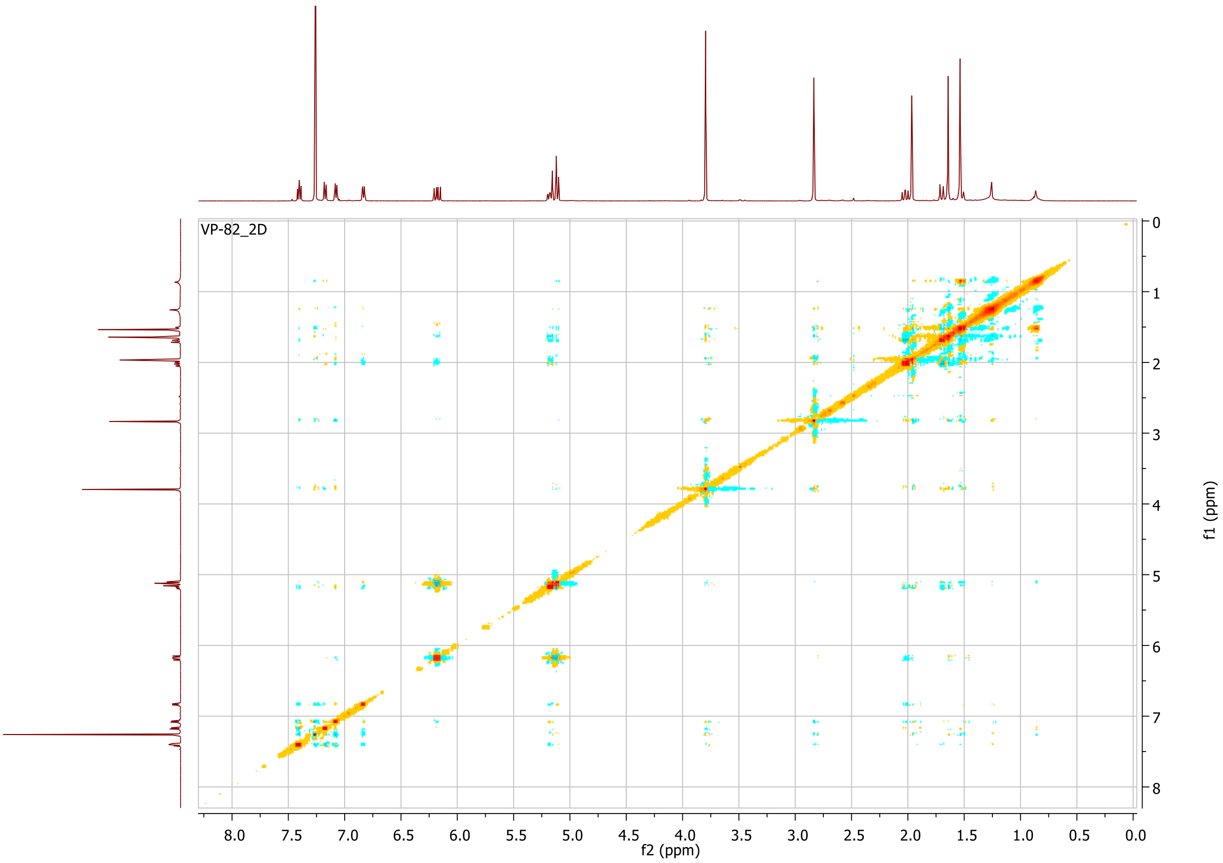

Figure S48. NOESY spectrum of **7** ($CDCl_3$, 500 MHz)

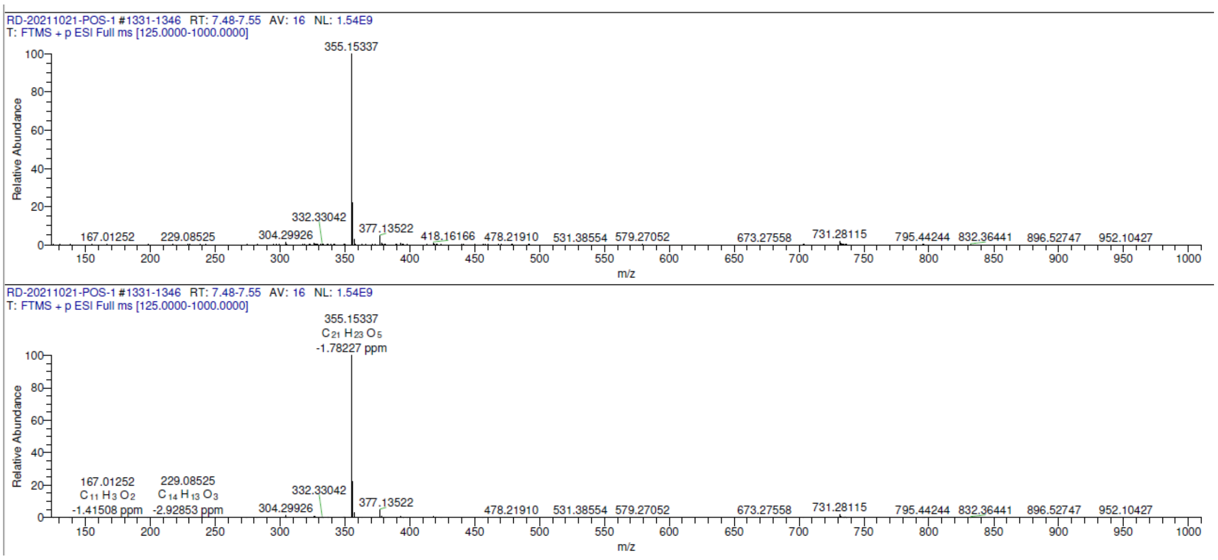

Figure S49. HRESIMS spectrum of **7**

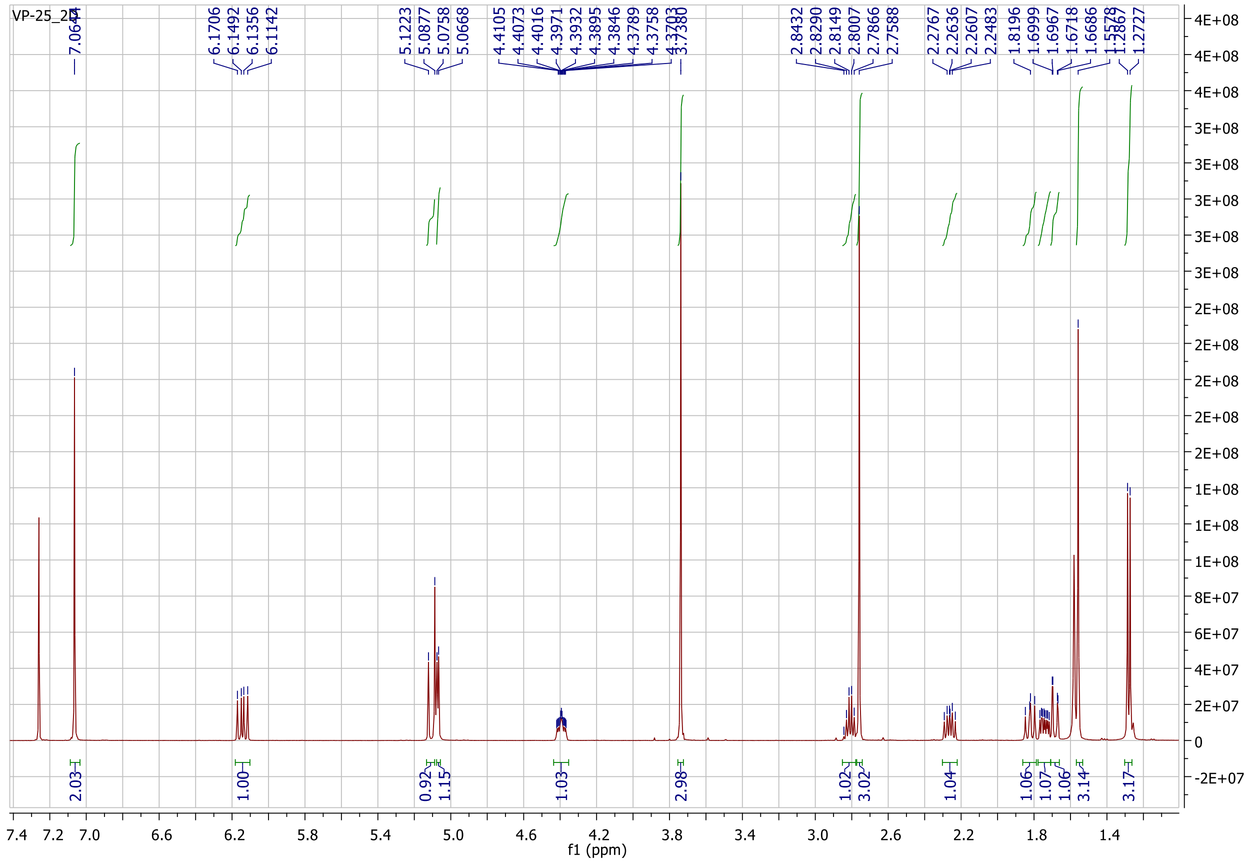

Figure S50. ^{1}H NMR spectrum of **8** ($CDCl_3$, 500 MHz)

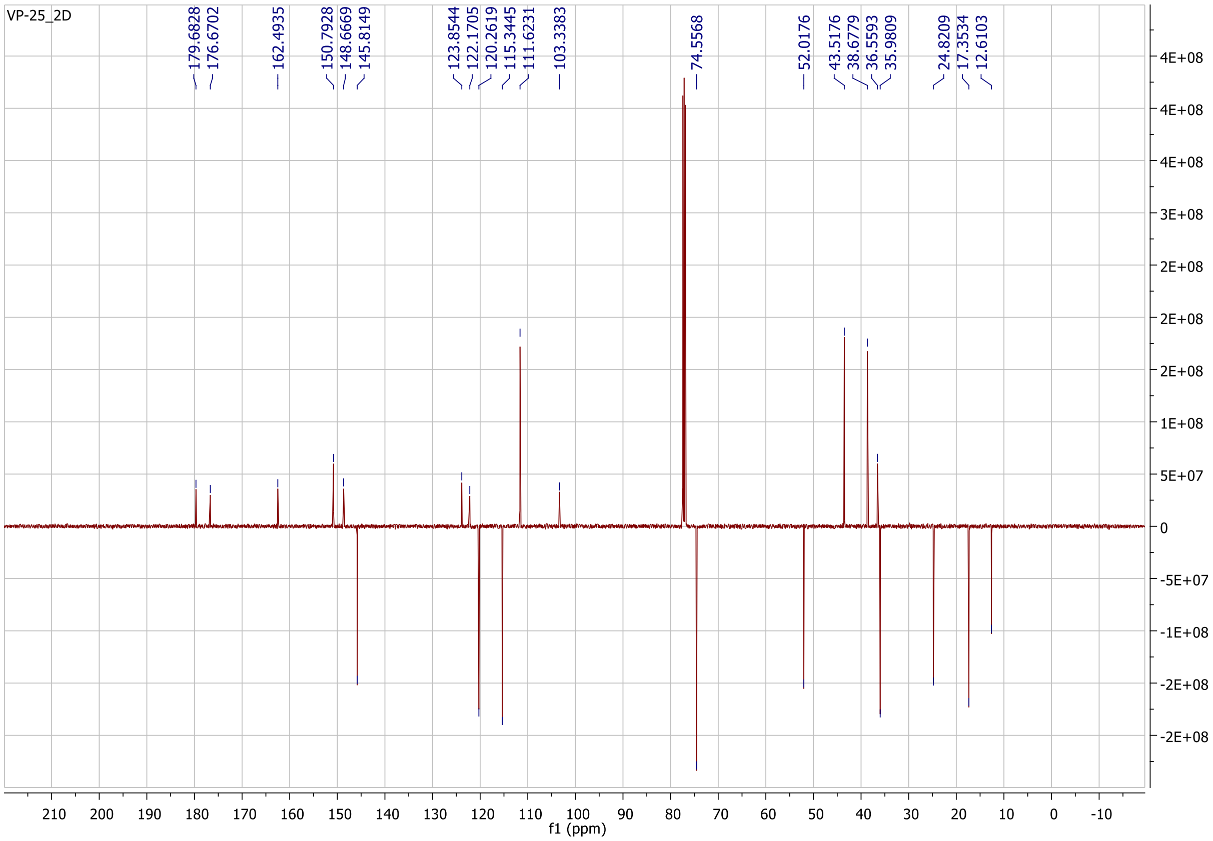

Figure S51. ^{13}C NMR JMOD spectrum of **8** ($CDCl_3$, 500 MHz)

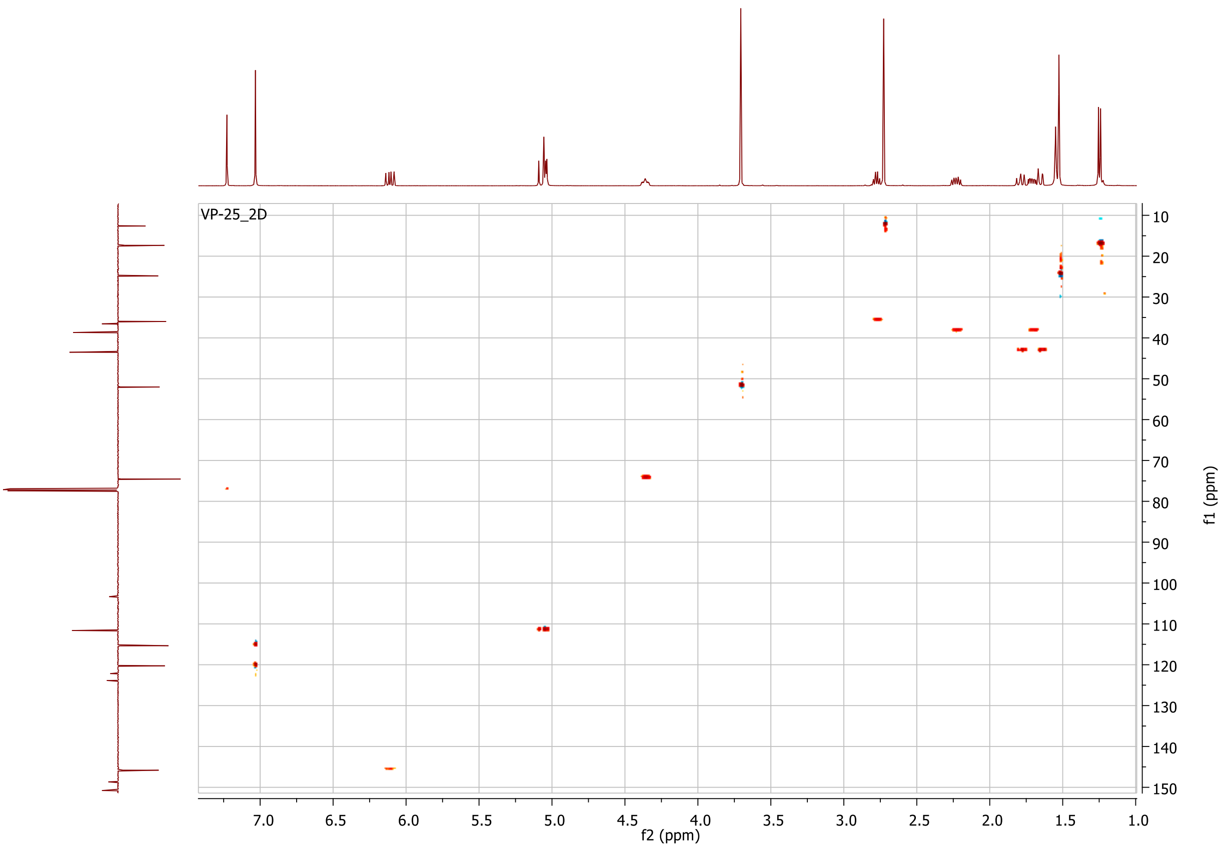

Figure S52. HSQC spectrum of **8** ($CDCl_3$, 125/500 MHz)

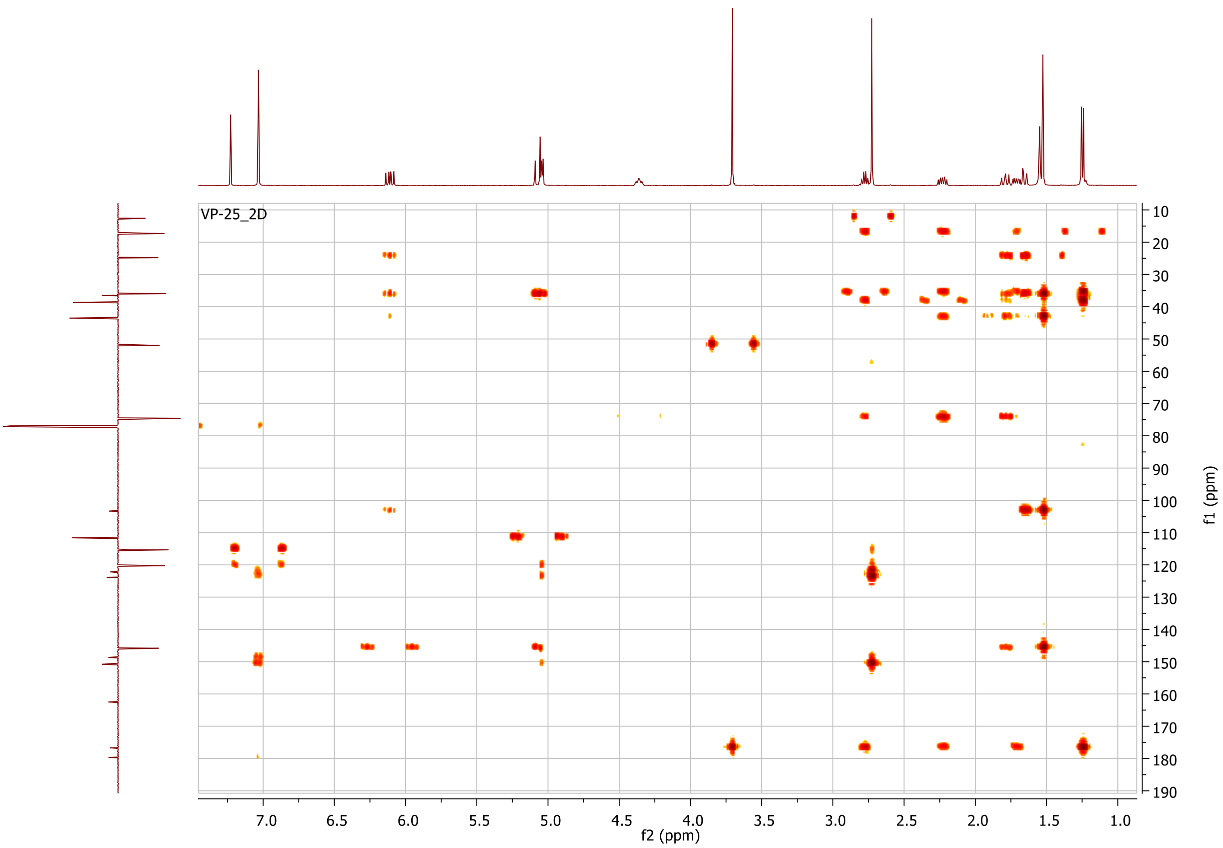

Figure S53. HMBC spectrum of **8** ($CDCl_3$, 125/500 MHz)

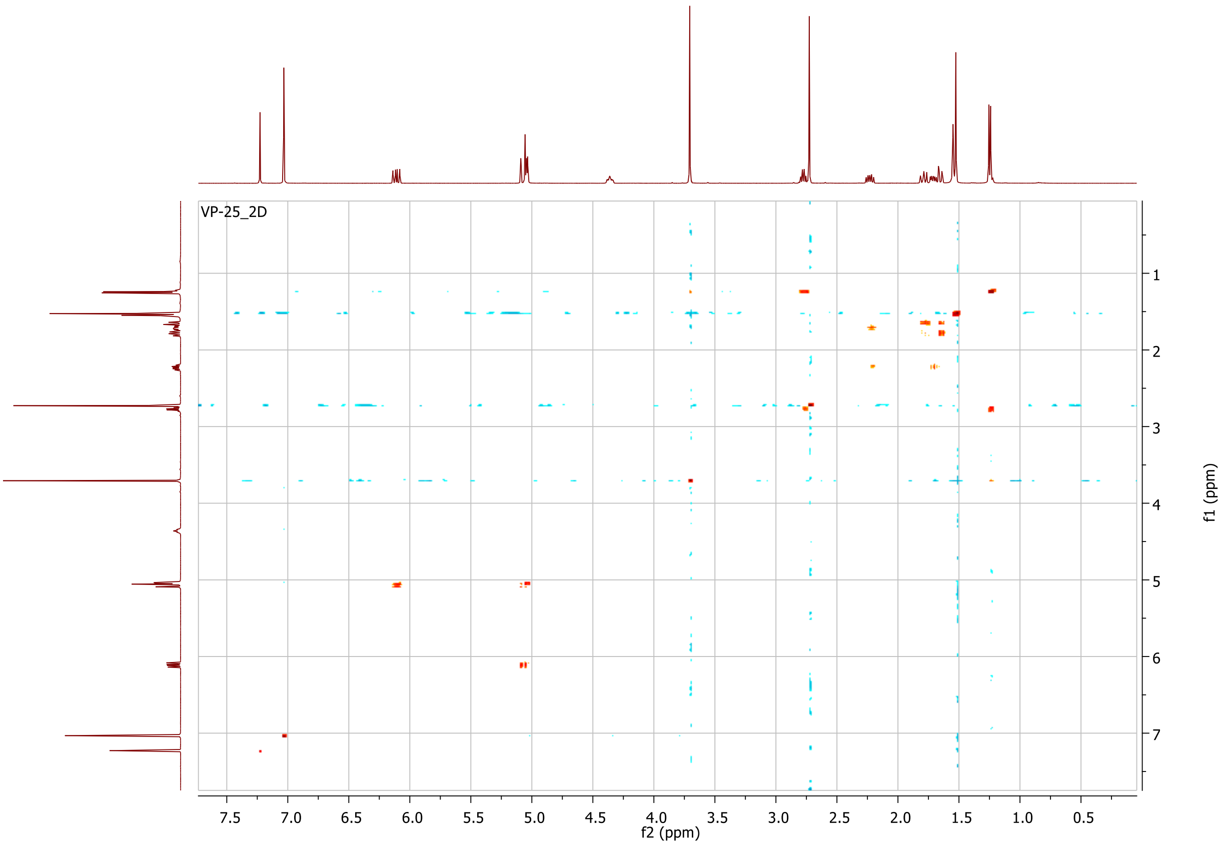

Figure S54. COSY spectrum of **8** ($CDCl_3$, 500 MHz)

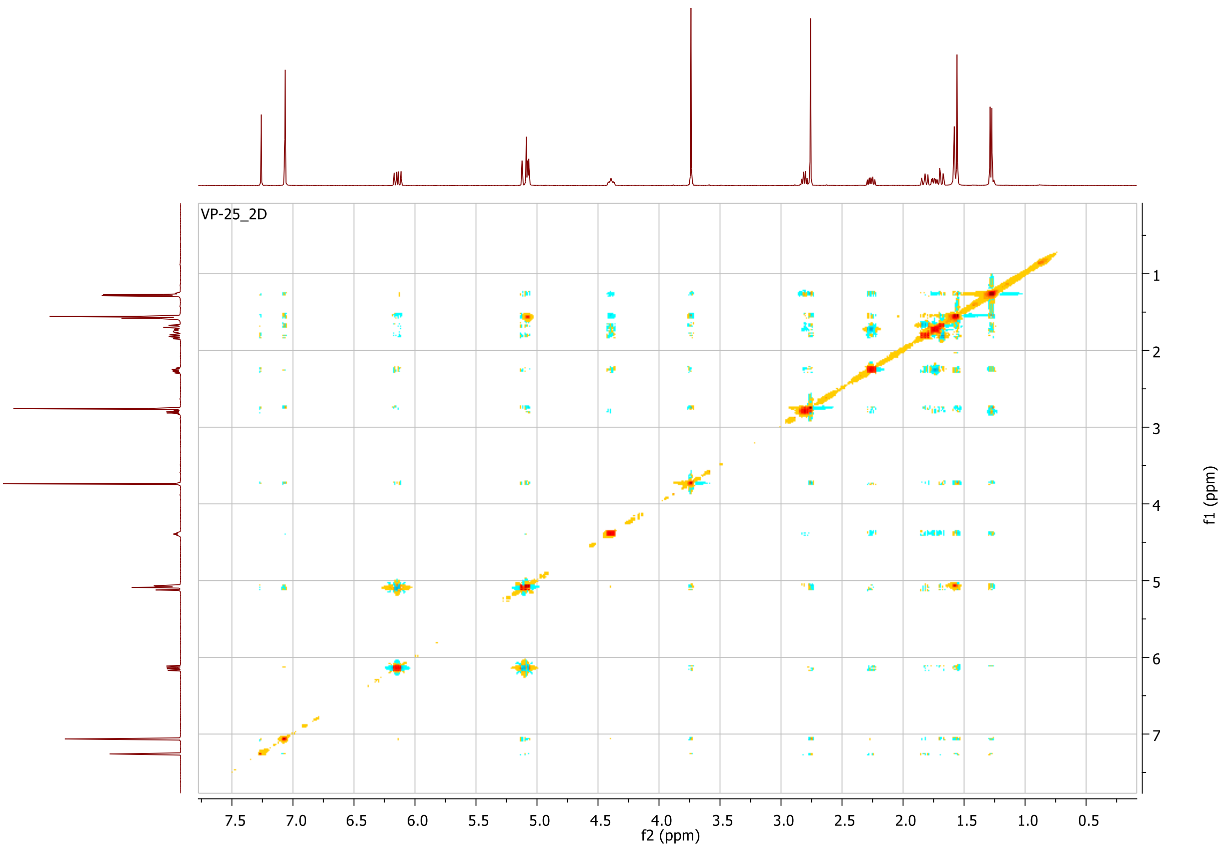

Figure S55. NOESY spectrum of **8** ($CDCl_3$, 500 MHz)

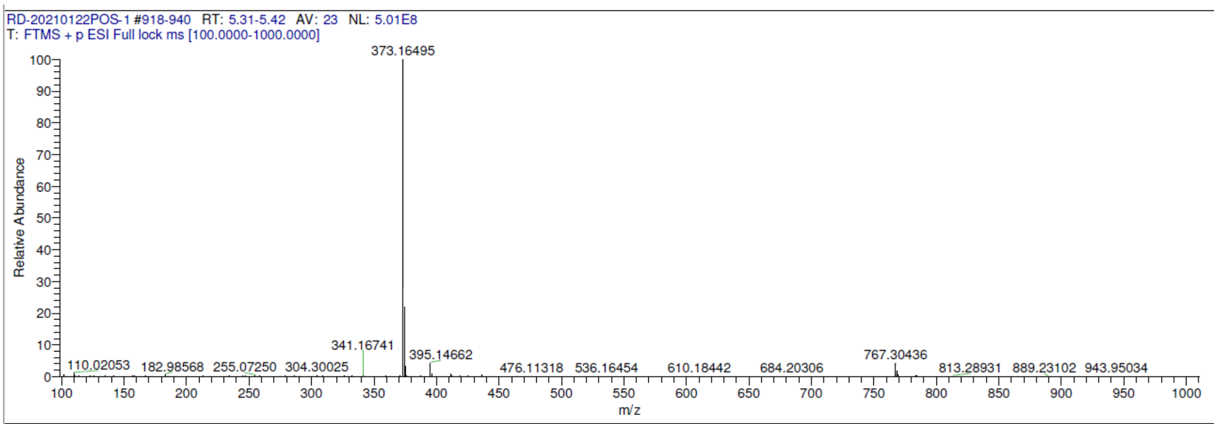

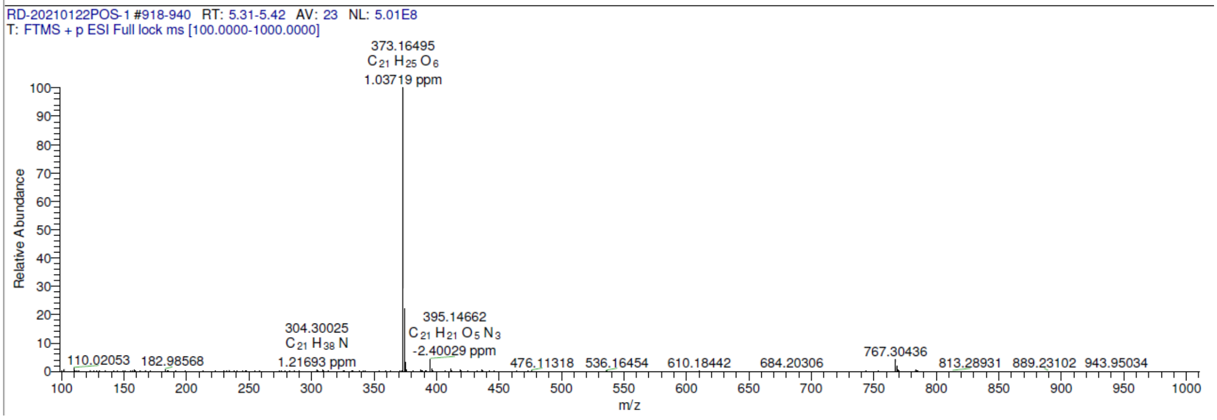

Figure S56. HRESIMS spectrum of **8**

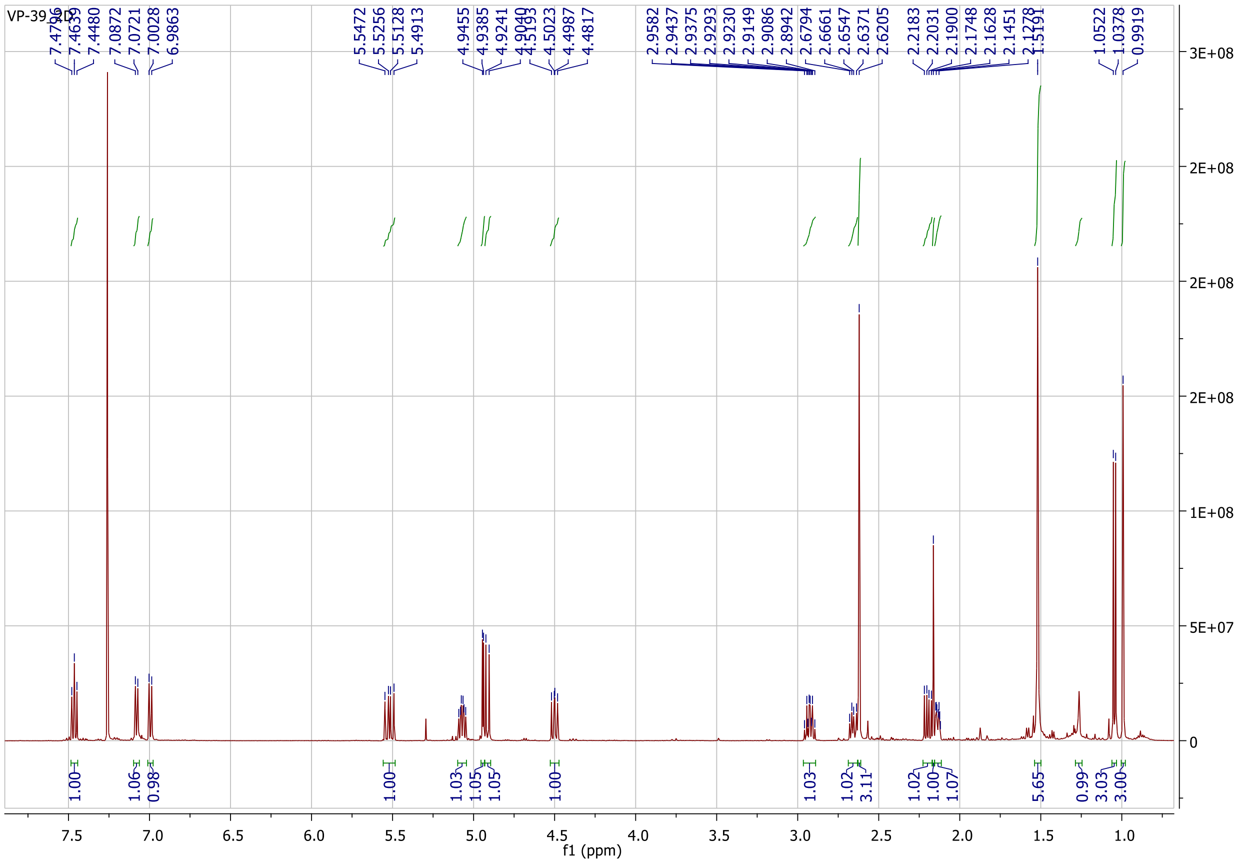

Figure S57. ^{1}H NMR spectrum of **9** ($CDCl_3$, 500 MHz)

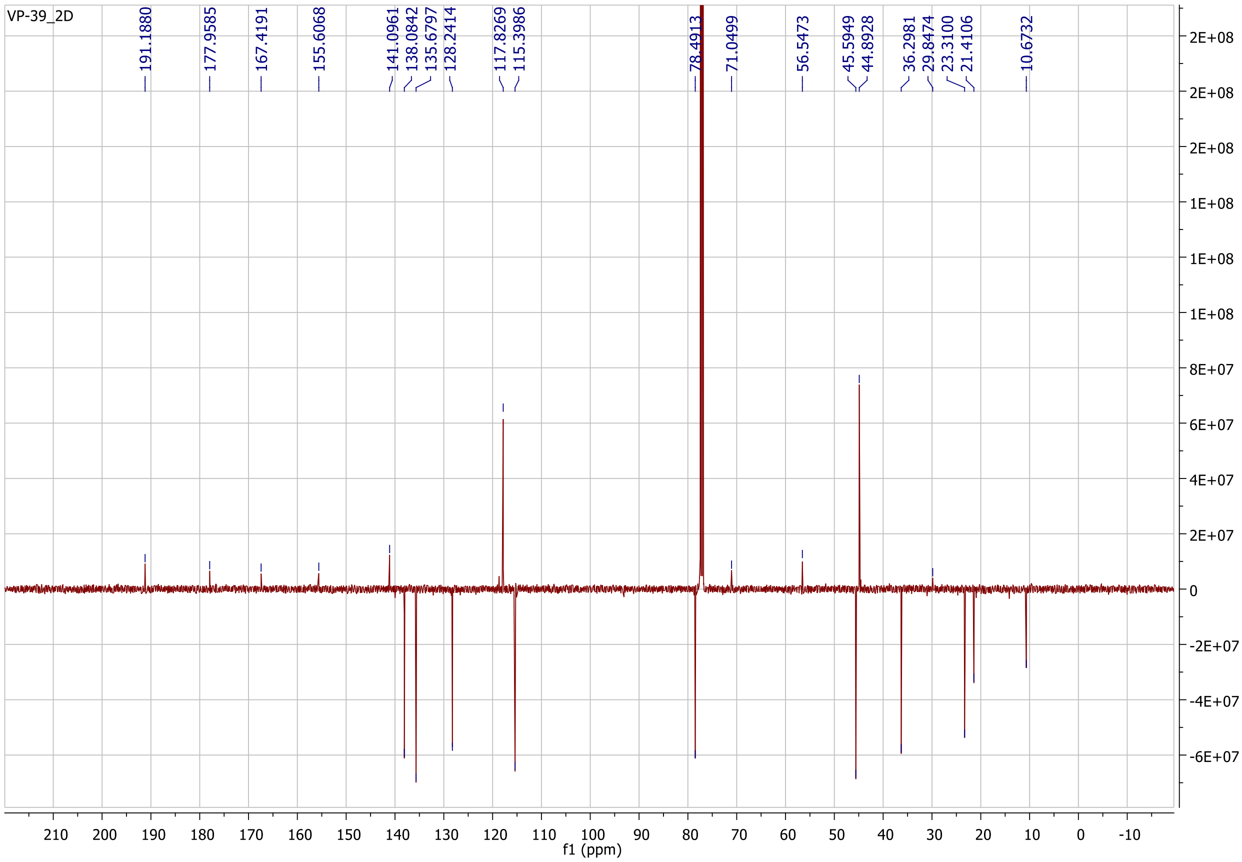

Figure S58. ^{13}C NMR JMOD spectrum of **9** ($CDCl_3$, 500 MHz)

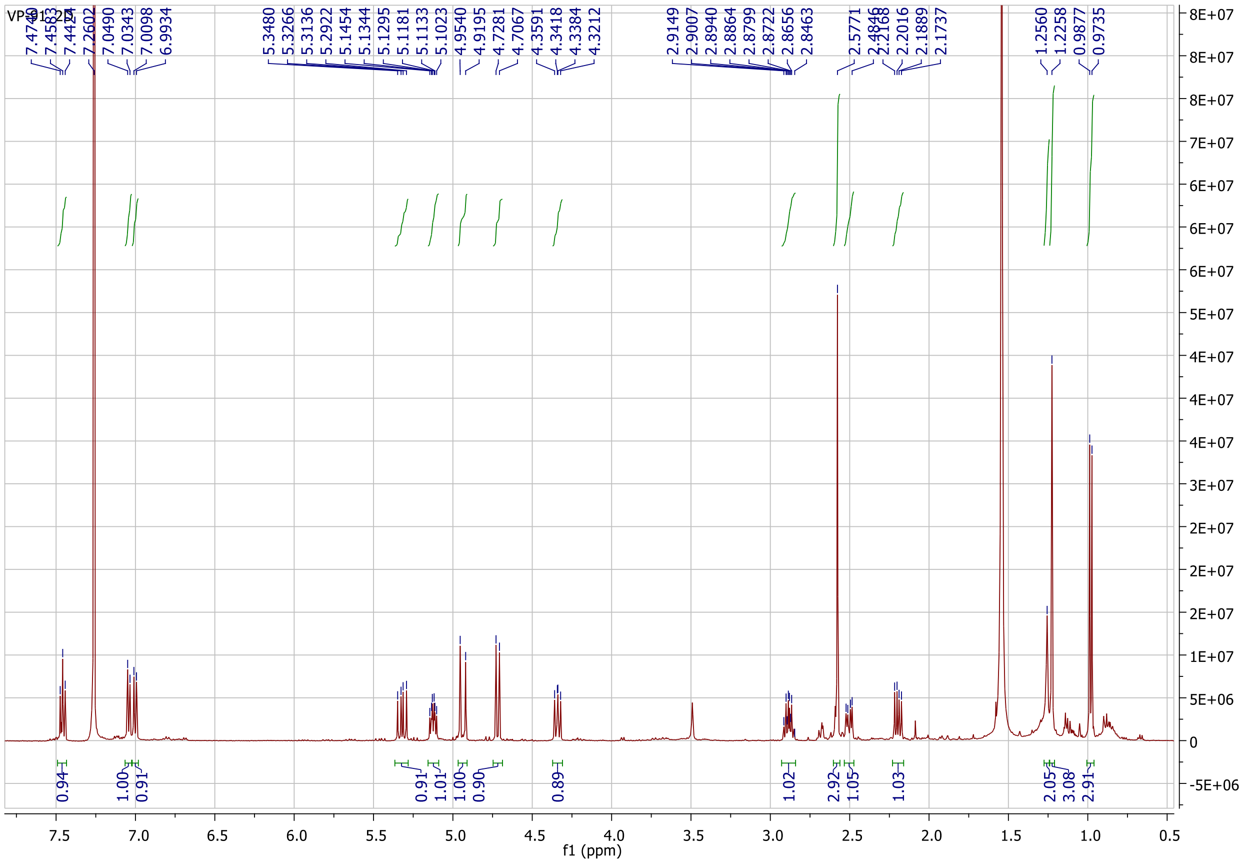

Figure S59. ^{1}H NMR spectrum of **10** ($CDCl_3$, 500 MHz)

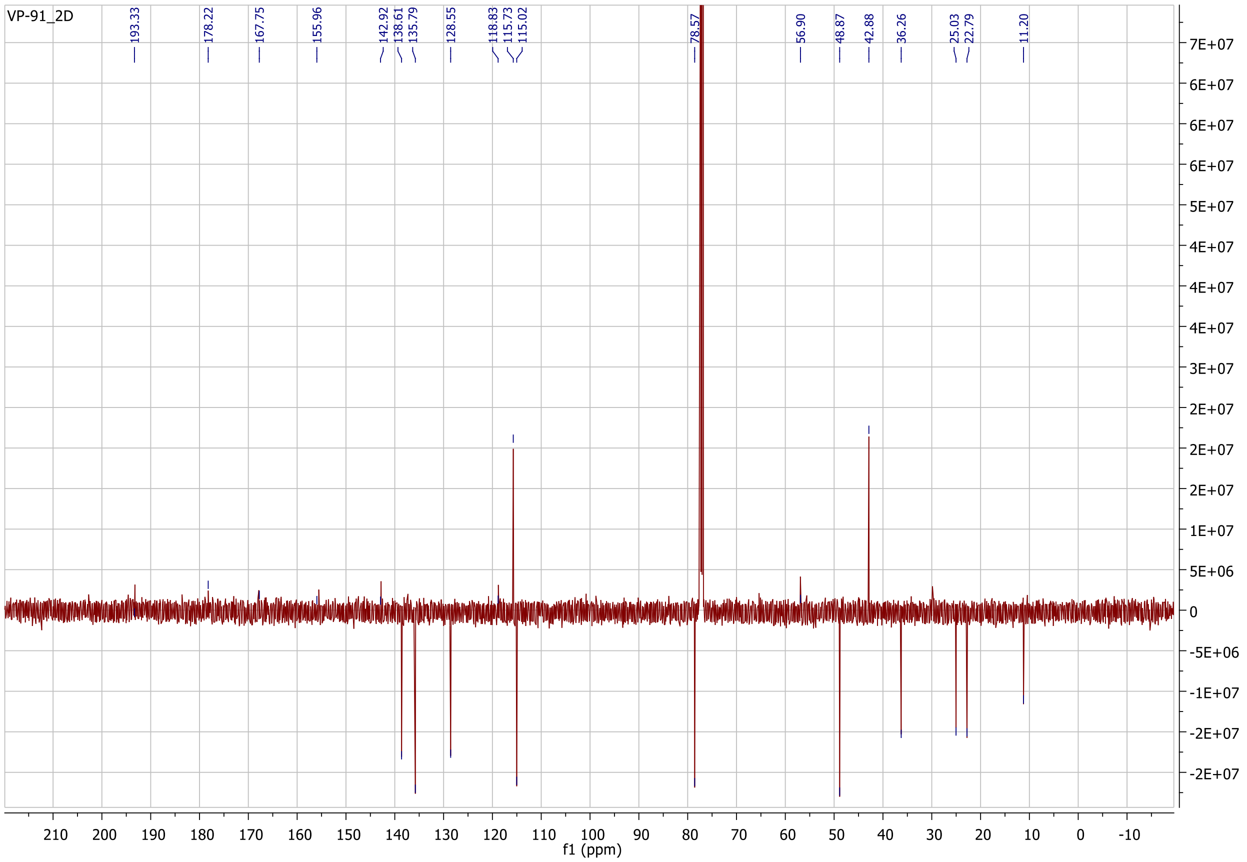

Figure S60. ^{13}C NMR JMOD spectrum of **10** ($CDCl_3$, 500 MHz)

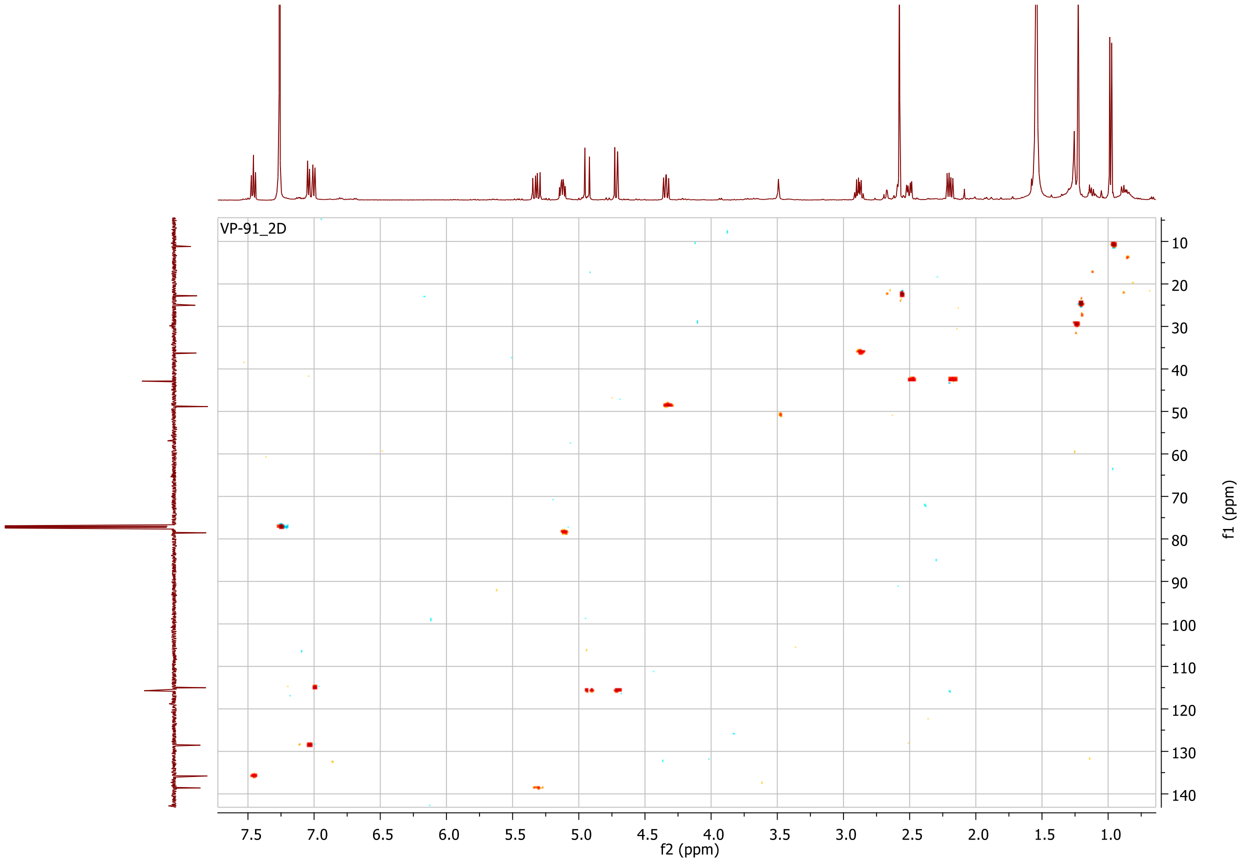

Figure S61. HSQC spectrum of **10** ($CDCl_3$, 125/500 MHz)

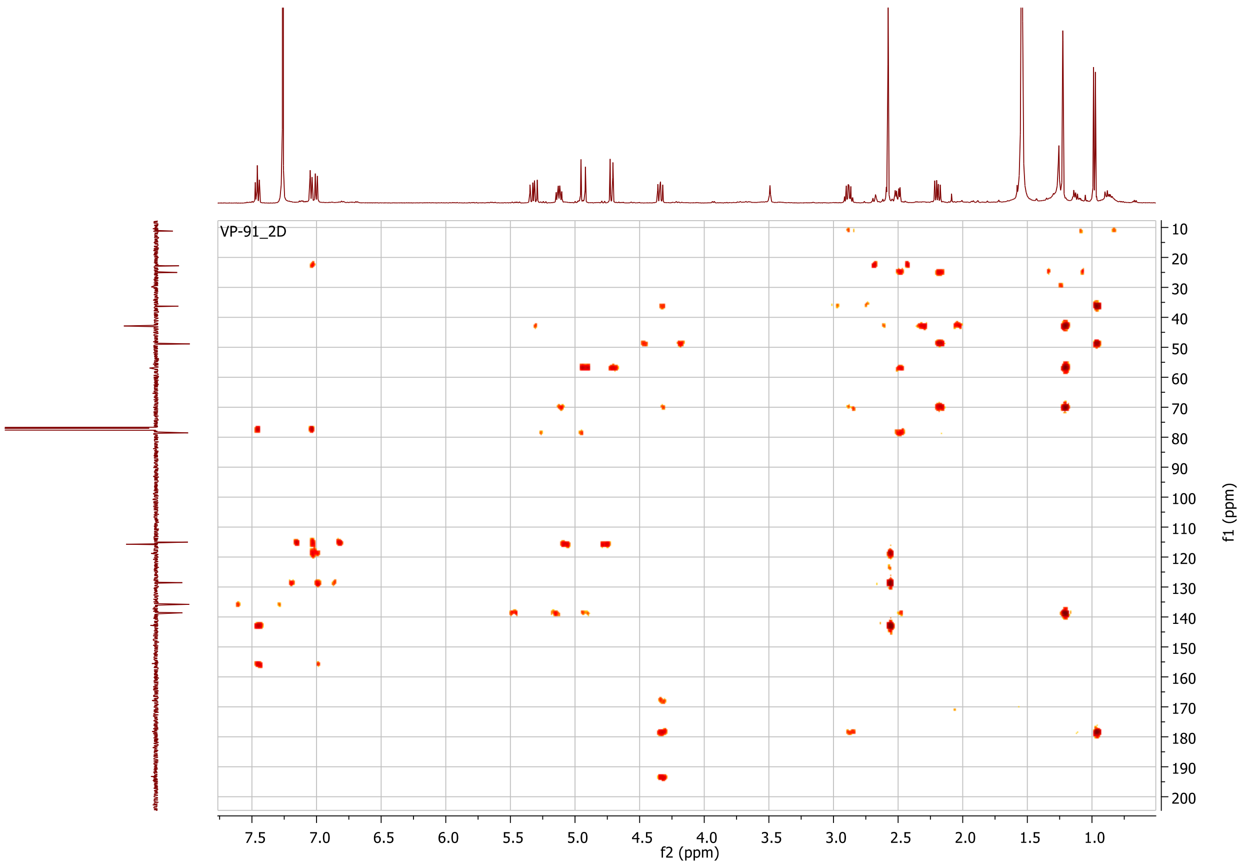

Figure S62. HMBC spectrum of **10** ($CDCl_3$, 125/500 MHz)

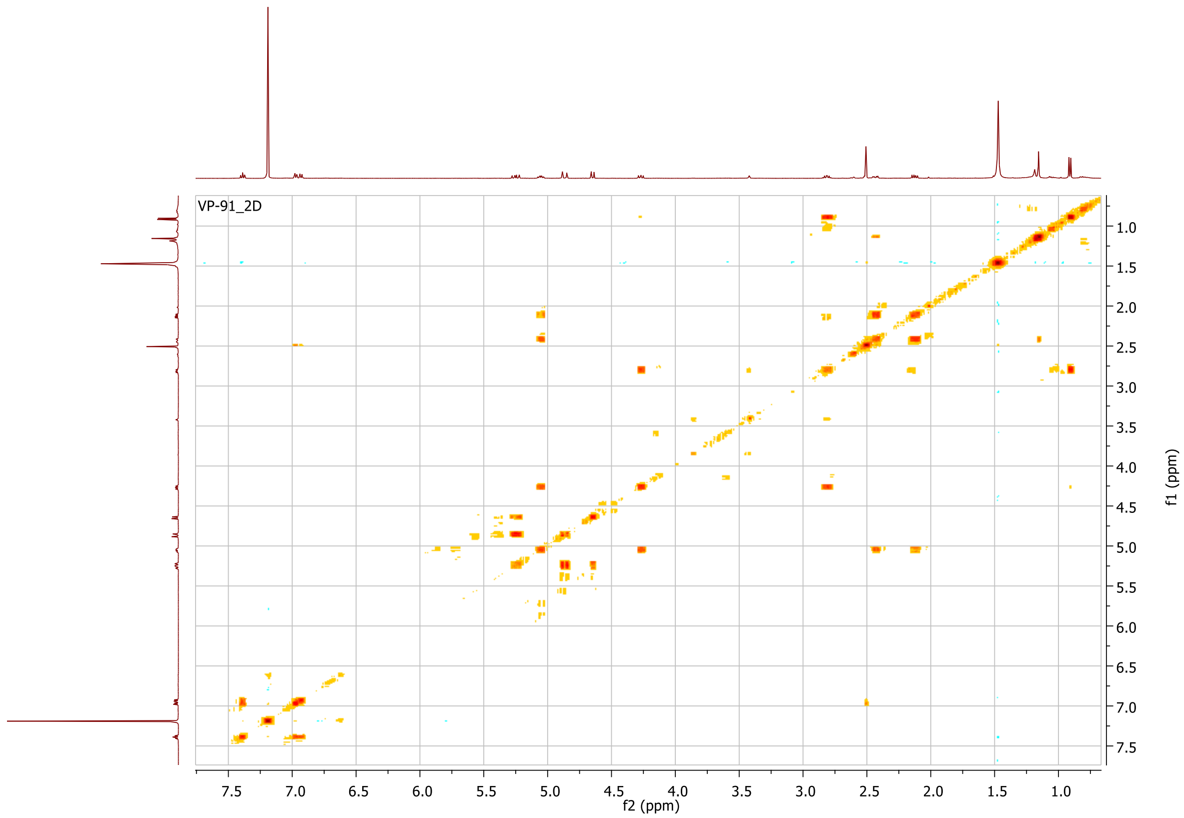

Figure S63. COSY spectrum of **10** ($CDCl_3$, 500 MHz)

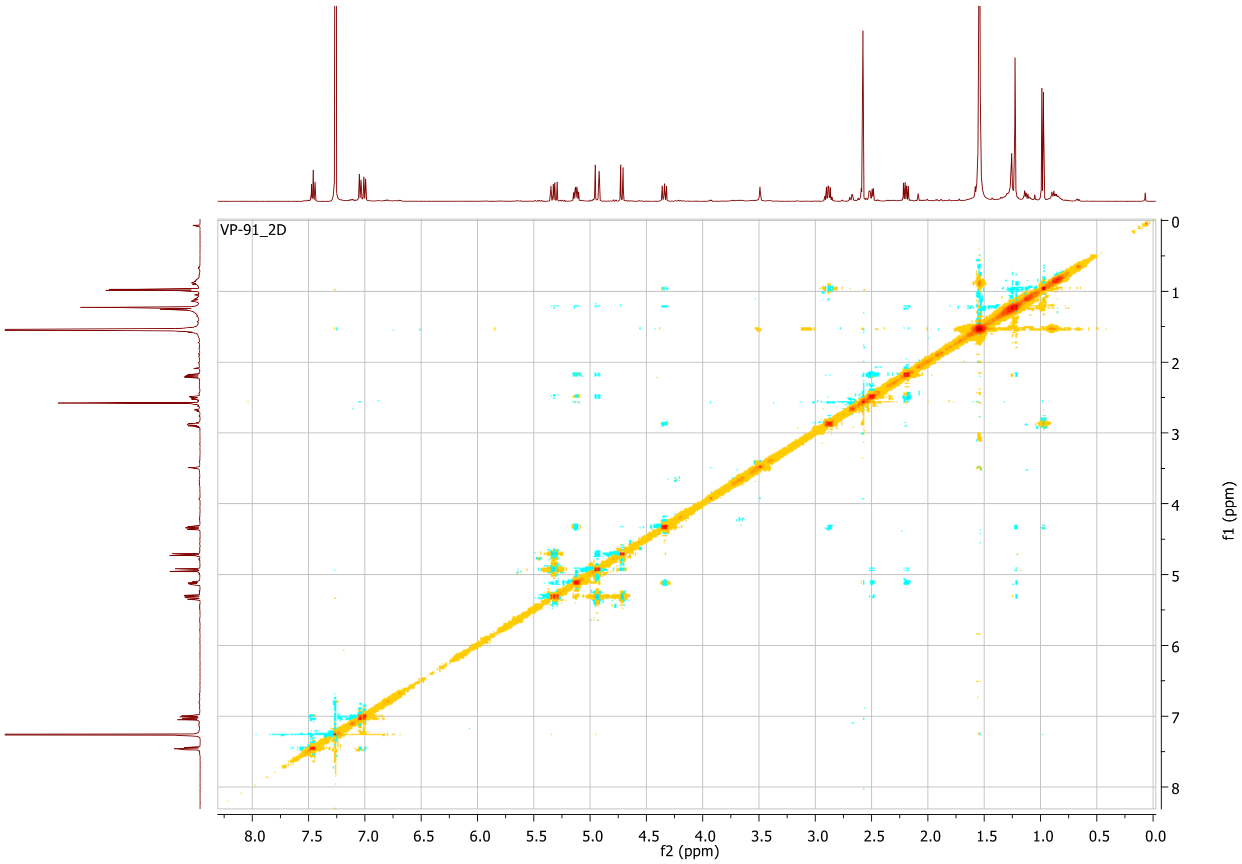

Figure S64. NOESY spectrum of **10** ($CDCl_3$, 500 MHz)

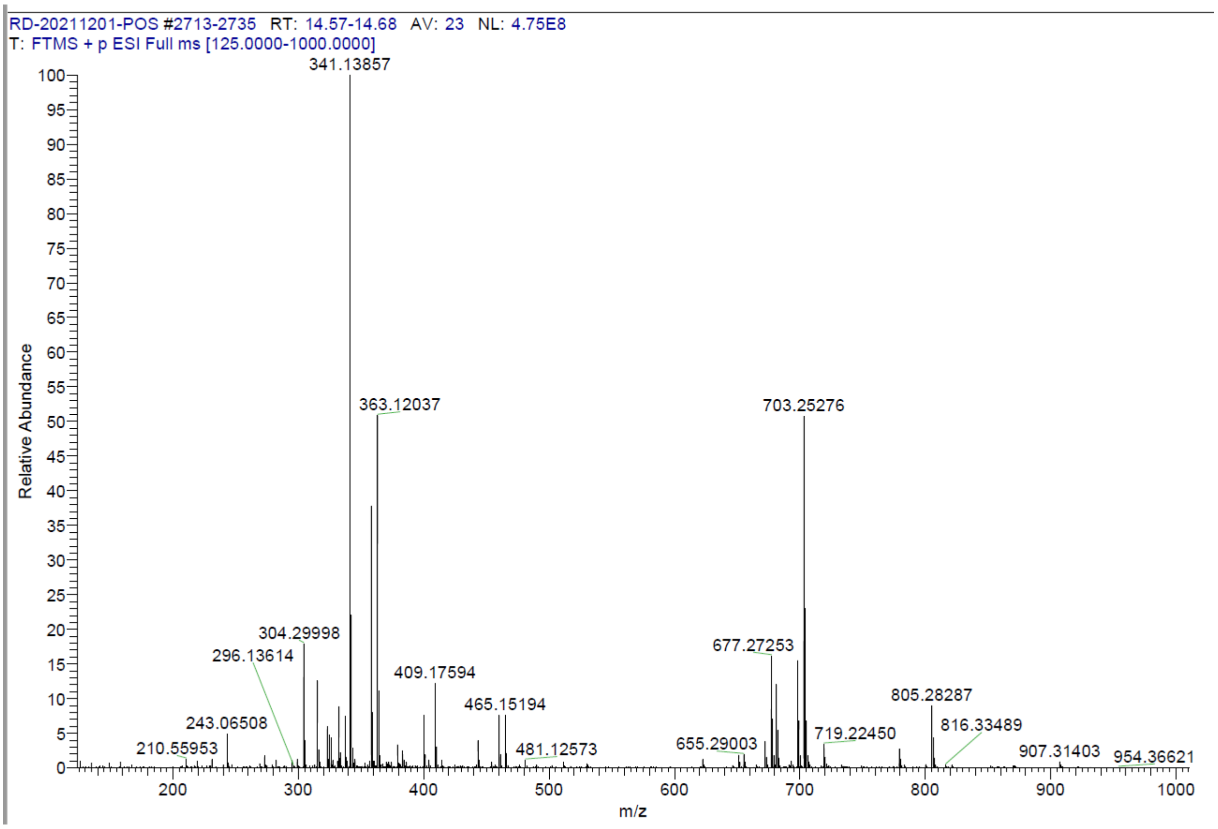

Figure S65. HRESIMS spectrum of **10**

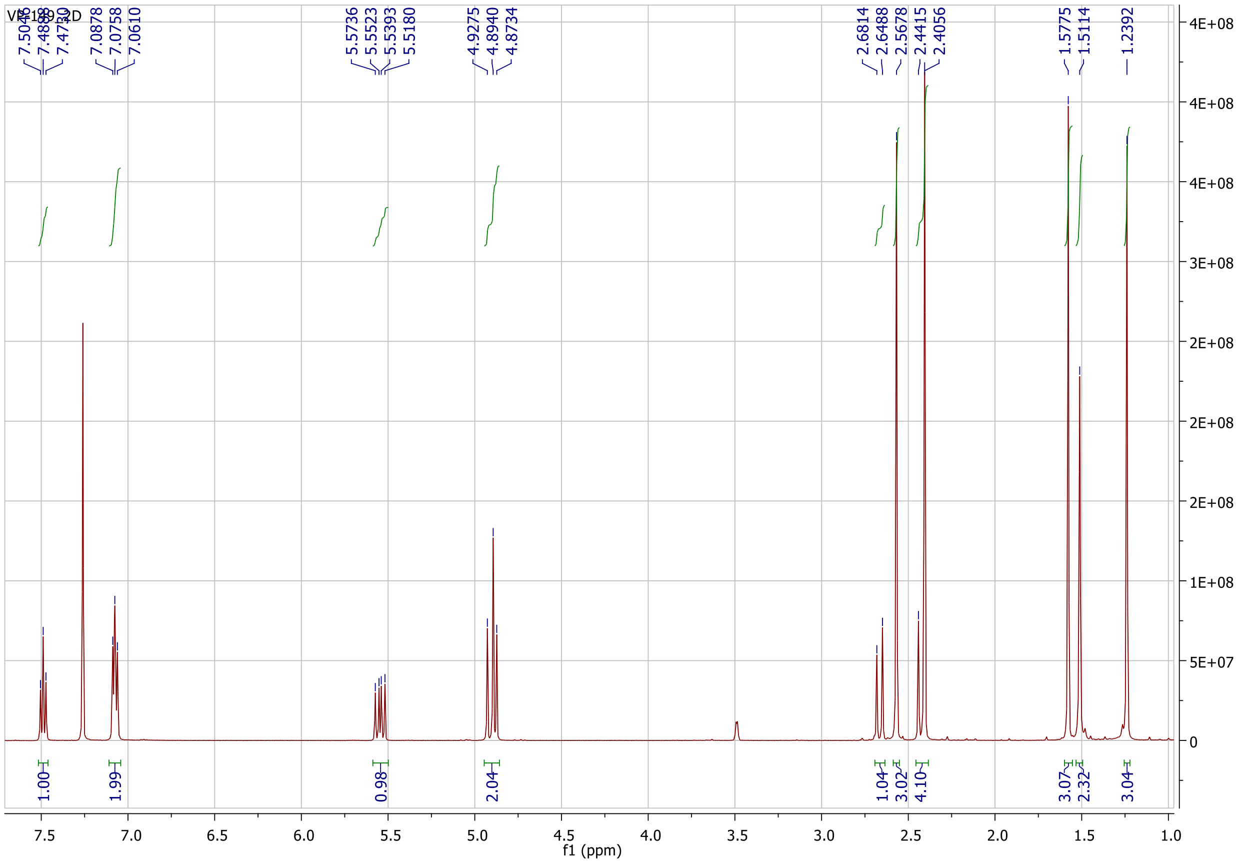

Figure S66. ^{1}H NMR spectrum of **11** ($CDCl_3$, 500 MHz)

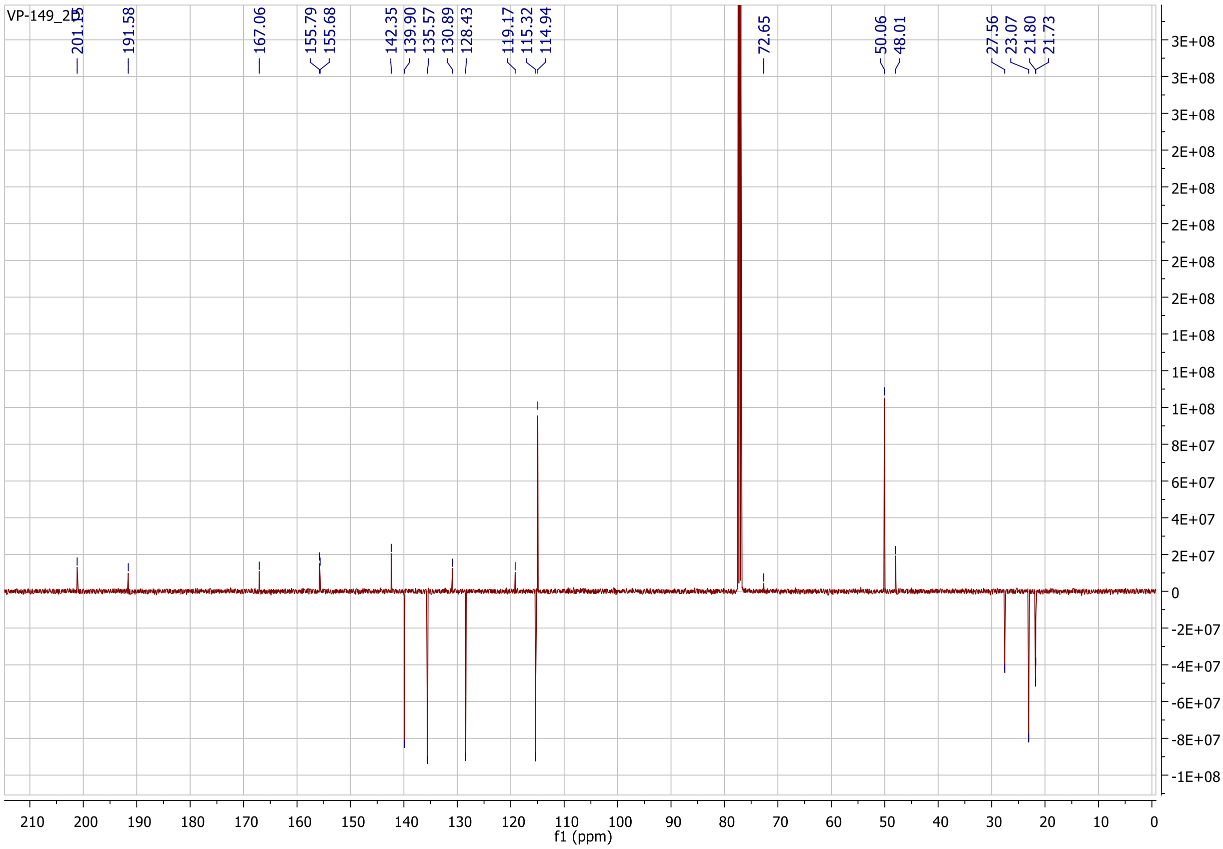

Figure S67. ^{13}C NMR JMOD spectrum of **11** ($CDCl_3$, 500 MHz)

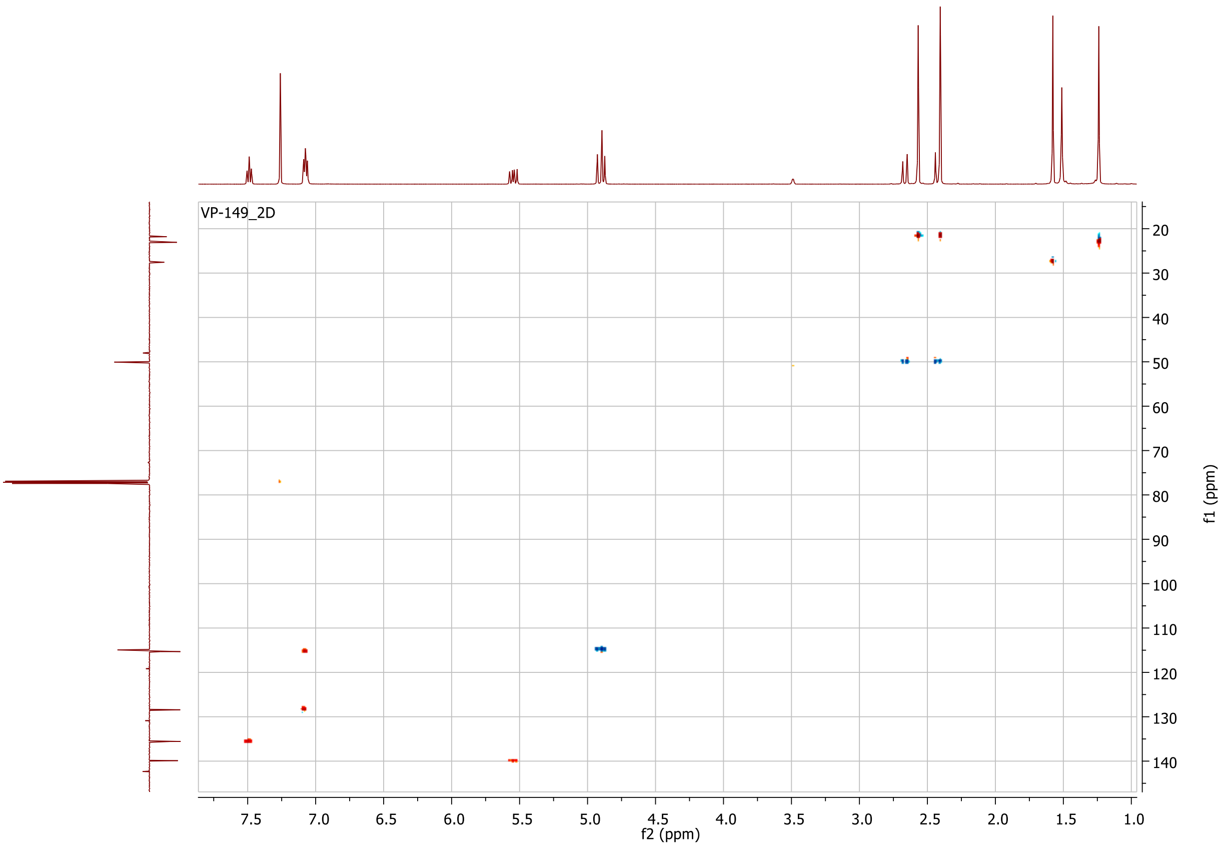

Figure S68. HSQC spectrum of **11** ($CDCl_3$, 125/500 MHz)

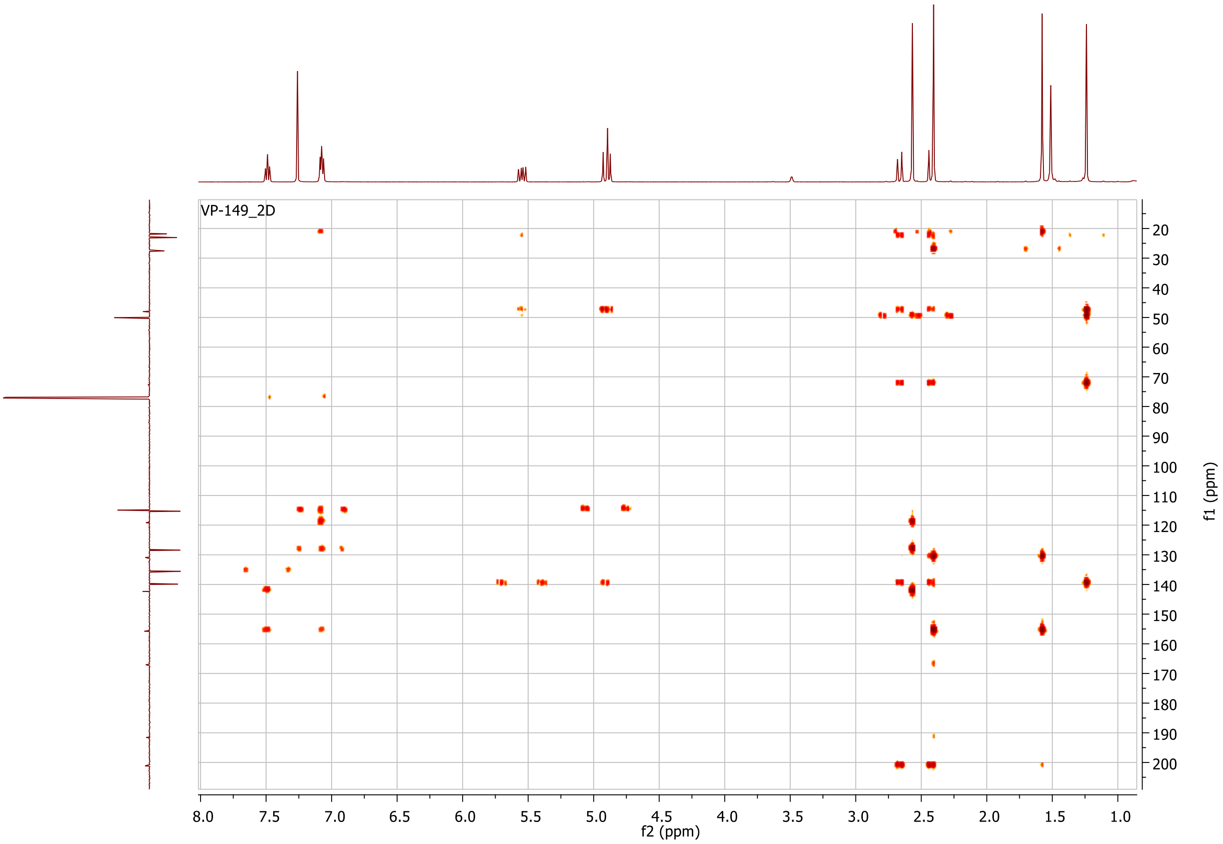

Figure S69. HMBC spectrum of **11** ($CDCl_3$, 125/500 MHz)

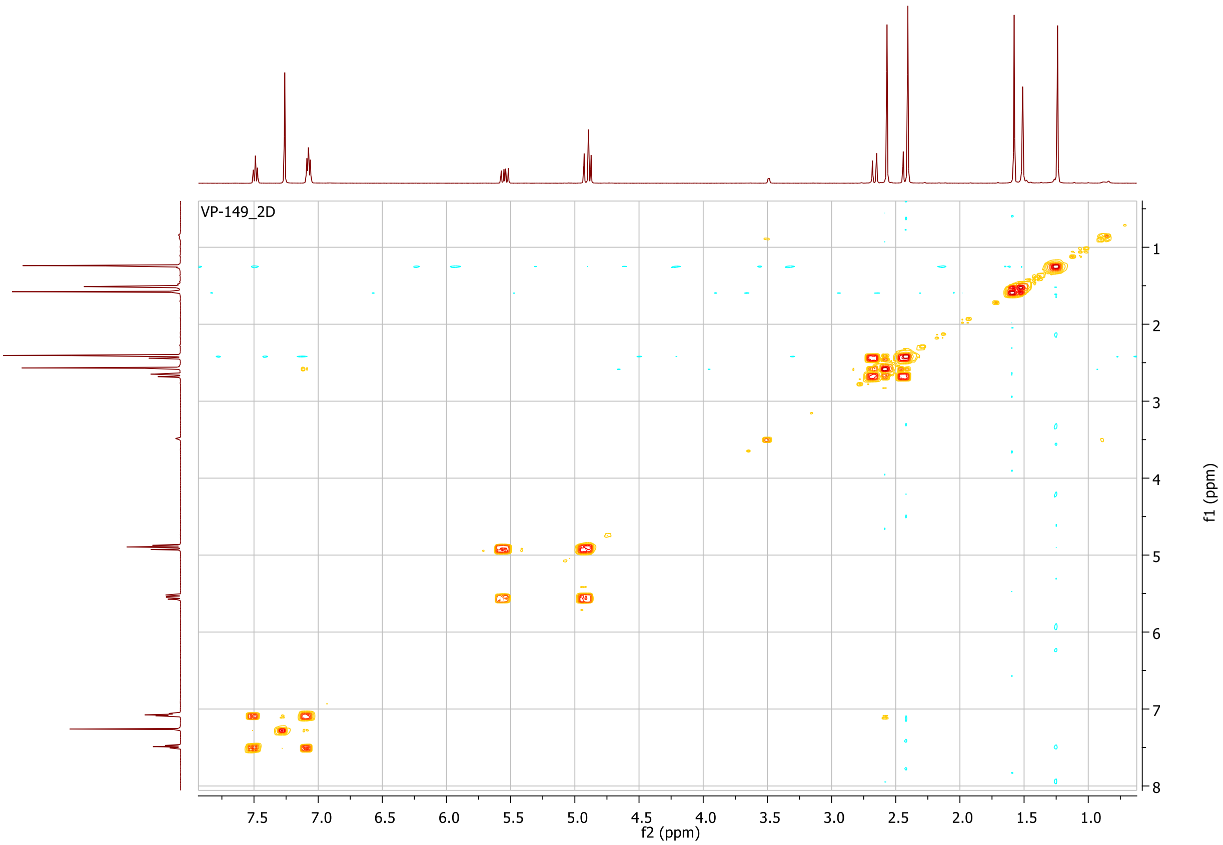

Figure S70. COSY spectrum of **11** ($CDCl_3$, 500 MHz)

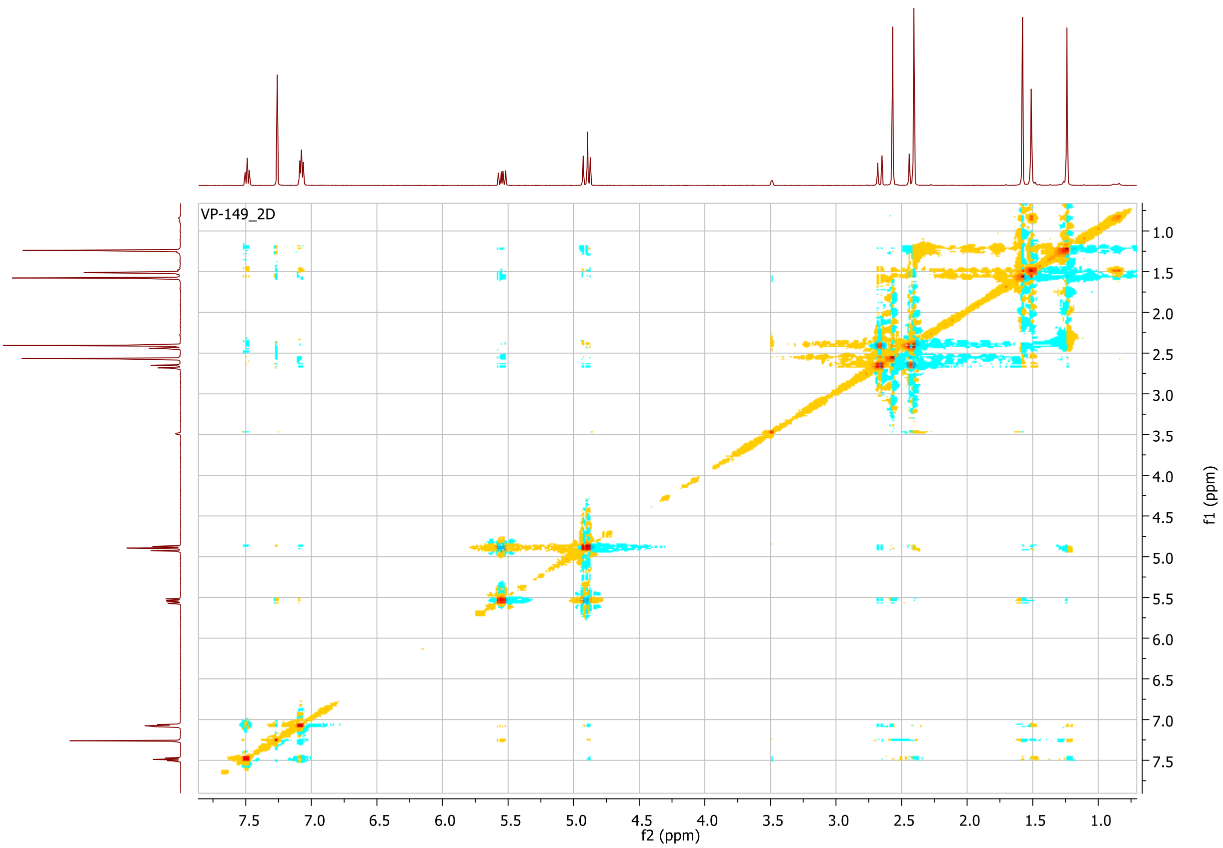

Figure S71. NOESY spectrum of **11** ($CDCl_3$, 500 MHz)

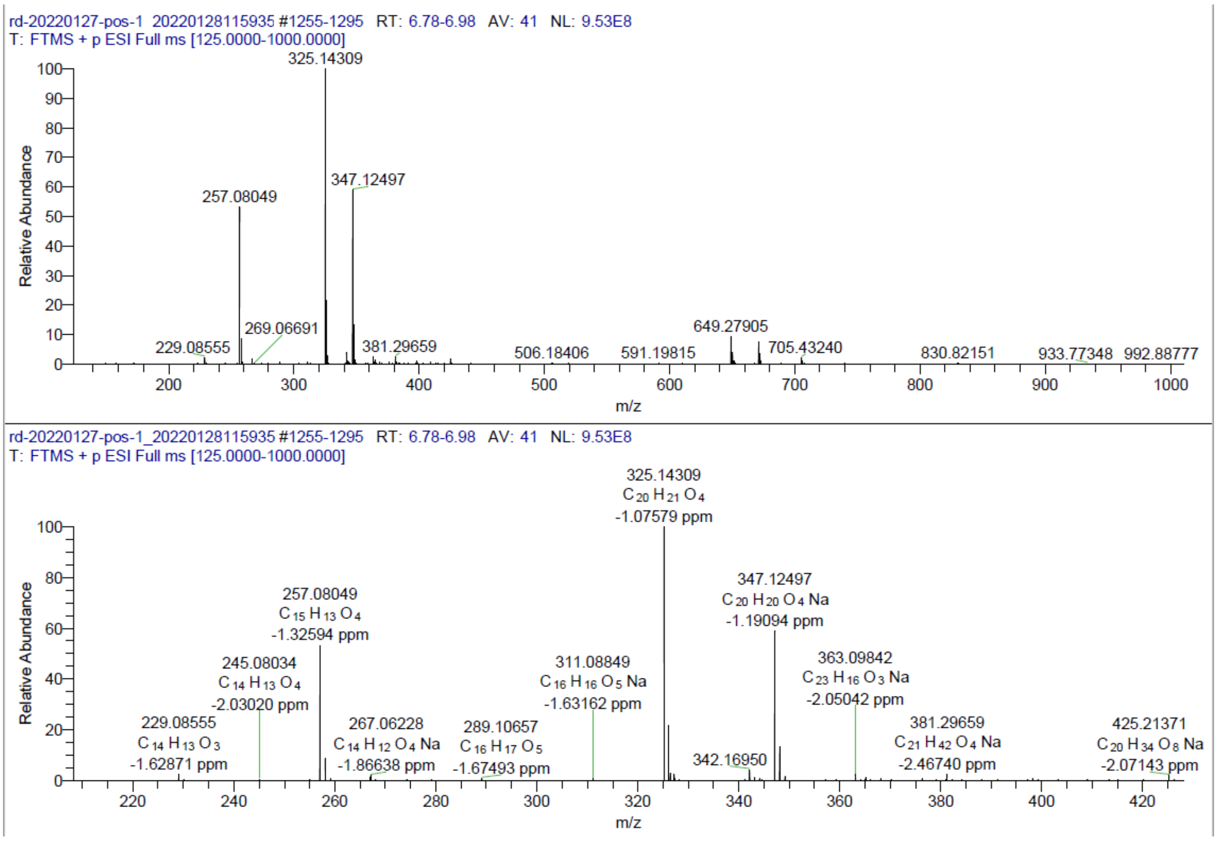

Figure S72. HRESIMS spectrum of **11**

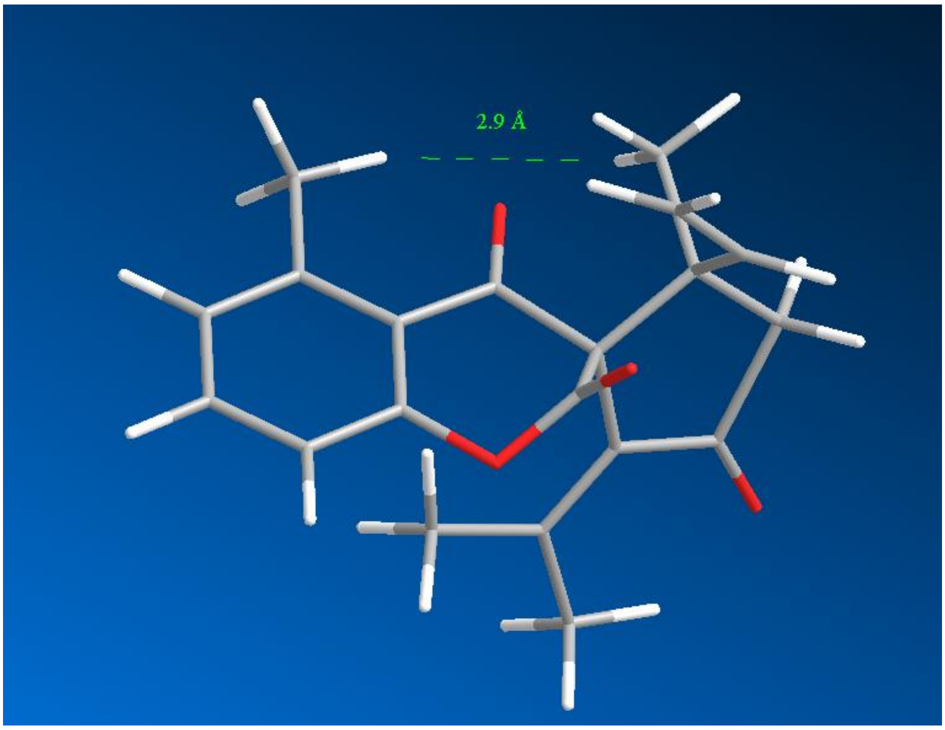

Figure S73. 3D structure of compound **10** (distance of H_3-9 and H_3-10' is 2.9 Å).
